# Sub-15 nm Nanoparticles for Drug Delivery: Emerging Frontiers and Therapeutic Potential

**DOI:** 10.3390/ijms262210842

**Published:** 2025-11-08

**Authors:** Tapas De, Vuong Trieu, Scott Myers, Sanjive Qazi, Saran Saund, Cynthia Lee

**Affiliations:** 1 Oncotelic Therapeutics, 29397 Agoura Road, Suite #107, Agoura Hills, CA 91301, USA; vtrieu@oncotelic.com (V.T.); scott.myers@oncotelic.com (S.M.); sanjive.qazi@autotelicinc.com (S.Q.); 2 Sapu Bioscience, 10840 Thornmint Road, Suite #118, San Diego, CA 92127, USA; saran.saund@sapubio.com (S.S.); cynthia.lee@sapubio.com (C.L.)

**Keywords:** ultrasmall nanoparticles, sub-15 nm nanoparticles, drug delivery, cancer therapy, nanomedicine, targeted therapy, controlled release, nanotoxicity, regulatory challenges

## Abstract

Nanoparticles (NPs) have significantly changed the field of drug delivery, offering control over pharmacokinetics, biodistribution, and targeted therapy. Among these, ultrasmall nanoparticles (USNPs) with sizes of approximately 5–15 nm have garnered significant interest due to their unique physicochemical properties, including enhanced cellular uptake, deeper tissue penetration, and prolonged systemic circulation. This review explores the fundamental principles governing sub-15 nm nanoparticles, their classification, and their distinctive advantages in pharmaceutical applications. Various types of nanoparticles, including polymeric, lipid-based, metallic, and carbon-based nanosystems, are examined in the context of drug delivery in cancer therapy. We detail how sub-15 nm polymeric nanoparticles (PNPs) are emerging as transformative drug delivery platforms for cancer therapy. The impact of nanoparticle size, surface modifications, and biocompatibility on therapeutic performance is critically analyzed. Furthermore, we discuss emerging applications of these ultrasmall nanoparticles in cancer therapy, neurological disorders, vaccine delivery, and imaging. Despite their promise, key challenges such as stability, aggregation, toxicity, and regulatory concerns remain significant hurdles for clinical translation. This review provides insights into the potential of 5–15 nm nanoparticles to reshape modern drug delivery and highlights future directions for research and development in this rapidly evolving field.

## 1. Introduction

Nanotechnology has revolutionized modern biomedical science [1], offering unprecedented tools for targeted drug delivery, diagnostics, and theranostics medicine. Nano-enabled healthcare is a large and rapidly expanding market. Recent estimates place global nanomedicine as being worth $242–294 B in 2024 (projected to rise to $571–779 B by ~2032–2033), with nanotechnology drug delivery accounting for ~$109 B in 2025 (CAGR ≈ 10% to 2030). The platforms dominated by small nanoparticles are notable growth drivers: lipid nanoparticles (LNPs) used for RNA therapeutics were worth ~$0.8–1.0 B in 2024 (forecast ~$3.5 B by 2034, ≈13% CAGR), and nanoparticle-based rapid diagnostics (e.g., gold-NP lateral-flow assays) were worth ~$9.85 B in 2024. At the product level, LNP-enabled mRNA vaccines recorded ~$36 B in revenue in 2021 for one product alone. This highlights the industrial significance of small-size nanoparticle technologies.

Among the various categories of nanomaterials, nanoparticles (NPs) have been extensively explored for their unique physicochemical properties and versatile applications in healthcare. Traditionally, nanoparticles employed in medicine have ranged between 50 and 200 nm in diameter, providing benefits such as extended circulation time and passive tumor targeting via the enhanced permeability and retention (EPR) effect [2,3,4]. However, in recent years, there has been a paradigm shift toward the development of ultrasmall nanoparticles, specifically those below 15 nanometers in size. This sub-15 nm class of nanomaterials exhibits fundamentally distinct behaviors in biological environments, opening new avenues for precision medicine.

Sub-15 nm nanoparticles possess a significantly larger surface-area-to-volume ratio, unique quantum properties, and distinct diffusion and clearance kinetics compared to their larger counterparts [5]. These features not only enhance their ability to penetrate biological barriers—such as the tumor extracellular matrix and blood–brain barrier—but also reduce their tendency to accumulate in the reticuloendothelial system (RES), thereby enabling faster renal clearance [6]. For instance, nanoparticles smaller than ~5.5 nm in hydrodynamic diameter have been shown to be efficiently filtered by the kidneys, minimizing long-term accumulation in off-target organs [7]. These traits are particularly desirable in clinical settings requiring repeated dosing, minimized systemic toxicity, and efficient clearance.

However, the same surface-area-to-volume advantage that enables rich interfacial chemistry below ~15 nm also elevates surface energy, making sub-15 nm nanoparticles more prone to aggregation. In biological buffers, van der Waals attraction, electrolyte screening of charge, and protein-bridging coronas can rapidly convert ultrasmall dispersions into larger agglomerates—negating penetration/clearance benefits and distorting biodistribution. Practically, aggregation risk rises at near-neutral zeta potential (~±10 mV) and high ionic strength. These problems are mitigated by steric/hydration-layer stabilization at adequate graft density, core–shell encapsulation (e.g., lipid/micelle or polymer shells), buffer/lyophilization optimization, and benign-corona management.

In oncology, where deep tumor penetration and precise targeting remain major hurdles, sub-15 nm nanoparticles show significant promise. Their small size facilitates transvascular transport and interstitial diffusion within solid tumors, including poorly vascularized and desmoplastic tumors that are typically resistant to conventional nanocarriers [8]. Furthermore, the enhanced mobility of sub-15 nm systems can be leveraged to reach micrometastases and anatomical niches inaccessible to larger particles, such as bone marrow and brain parenchyma [9]. In parallel, advances in materials chemistry have enabled the development of sub-15 nm polymeric micelles, dendrimers, inorganic clusters, and protein-based carriers with high drug-loading capacity, tailored release profiles, and multifunctionality [10].

Despite these promising features, the design and development of sub-15 nm nanoparticles come with substantial challenges. Controlling size, shape, and surface properties with nanoscale precision is technically demanding, and conventional characterization tools often lack the resolution or sensitivity to accurately evaluate ultrasmall particles. Additionally, the altered biodistribution and faster clearance of sub-15 nm systems necessitate new pharmacokinetic models and toxicological paradigms. Regulatory guidance remains underdeveloped for this specific size domain, creating ambiguity in the clinical translation pathway [11].

This review aims to provide a comprehensive overview of sub-15 nm nanoparticles, covering their design principles, fabrication strategies, physicochemical and biological properties, and emerging biomedical applications. Special emphasis is placed on recent innovations in engineering ultrasmall nanoparticles for drug delivery and theranostics, their interactions with biological systems, and the current landscape of regulatory and quality considerations. Future perspectives are also discussed, highlighting the potential of these nanosystems to shape the next generation of precision nanomedicine. As part of the Supplementary Materials (Tables S1–S3), comprehensive lists of 5–15 nm nanoparticles that have been investigated as pharmaceuticals, diagnostics, and theranostics have been compiled, and the lists are provided as appendices in tabular form.

Our manuscript is, to our knowledge, the first comprehensive review that systematically integrates and analyzes literature across polymeric, lipid-based, metallic, and carbon/quantum-dot nanoparticles strictly within the 5–15 nm size domain, highlighting their distinct diffusion, clearance, and therapeutic profiles compared with conventional nanocarriers. This analytical approach allows readers to view the sub-15 nm domain not merely as a size reduction of conventional nanoparticles, but as a distinct frontier in nanomedicine with its own opportunities and challenges.

## 2. Types of Sub-15 nm Nanoparticles 

### 2.1. Sub-15 nm Polymeric Nanoparticles and Dendrimers for Cancer Drug Delivery

Polymeric nanoparticles (PNPs) have long been utilized as platforms to deliver drugs to malignant cells. Such particles can be broadly categorized into (1) nanocapsules, (2) nanospheres, (3) micelles, (4) dendrimers, (5) polymersomes, and (6) polyplexes, depending on their structure and where and how the drug cargos are loaded (Table 1).

Polymeric Micelles (PM) are generally applied in systems where the hydrophilic part of the amphiphilic polymer is directed outwards, and the lipophilic part is directed to the core of the micelles. In the case of reverse micelles, the hydrophilic part of the amphiphilic polymer is directed towards the core, and the lipophilic part is directed outwards.

Polymeric nanoparticles (PNPs) have emerged as a versatile platform for cancer drug delivery owing to their tunable size, surface chemistry, biocompatibility, and ability to encapsulate both hydrophilic and hydrophobic therapeutic agents [13,14,15]. For the design of sub-15 nm PNPs, careful selection of polymer type and molecular architecture is crucial to ensure small particle size without compromising drug loading and stability. Among polymeric systems, linear amphiphilic block copolymers and dendritic polymers (dendrimers) have shown particular promise in achieving ultrasmall particle dimensions suitable for deep tumor penetration and improved pharmacokinetics.

#### 2.1.1. Polymeric Nanoparticles

PMs are made from mainly diblock polymers, triblock polymers, or graft polymers with hydrophilic and hydrophobic portions. They can be made up of ionic copolymers with ionic and hydrophilic parts. Based on the intermolecular forces controlling the segregation of micelles in aqueous surroundings, block copolymers are divided into three types. They are amphiphilic micelles (hydrophobic interactions), polyion complex micelles (electrostatic interactions), and micelles formed by metal complexations [16]. The drug can be encapsulated in different regions of the PMs according to the polarity. Nonpolar drugs on the core, polar drugs on the shell, and drugs with intermediate polarity encapsulated between the core and the shell [17].

Conventional polymers such as poly(lactic-co-glycolic acid) (PLGA), polyethylene glycol (PEG), poly(ε-caprolactone) (PCL), and polylactic acid (PLA) have been widely used for nanoparticle formulation. However, these often yield particles > 50 nm when used alone. To reduce particle size below 15 nm, researchers have turned to low molecular weight amphiphilic block copolymers such as PEG-b-PLA, PEG-b-PCL, and poly(2-oxazoline)-based systems, which self-assemble into micelle-like nanoparticles with hydrodynamic diameters as low as 10–20 nm [18,19]. PEG-ZW800 nanoparticles were fabricated with sizes around 10.9 nm. These nanoparticles demonstrated improved tumor-to-background ratios and longer circulation times when optimized at approximately 10 nm [20]. Polystyrene (PS) nanoparticles were prepared with a range of sizes, including 26 nm [21,22]. PEG-b-poly(glutamic acid) copolymers micelles had sizes as small as 30 nm. The 30 nm micelles demonstrated superior penetration into poorly permeable tumors [23]. The key parameters affecting size include polymer hydrophilic–hydrophobic balance, concentration, and the choice of solvent during nanoprecipitation or self-assembly.

Block copolymers can also be engineered to respond to pH, redox, or enzymatic triggers in the tumor microenvironment, enabling controlled drug release [24,25].

One example of nanoparticles in the range of 5–15 nm is the polymer nanocarrier system that was designed for prostate cancer targeting. In this work, researchers synthesized starPEG nanocarriers that are approximately 15 nm in size (40 kDa) and modified them with prostate-specific membrane antigen (PSMA)–targeting ligands (ACUPA) [26].

Single-chain polymer nanoparticles (SCNPs) are highlighted as an emerging platform for drug delivery due to their extremely small size (5–15 nm) and tunable properties. The study prepared a library of SCNPs with varying charge types (neutral, anionic, cationic, and zwitterionic) and demonstrated that surface properties play a critical role in cellular uptake [13]. It was found that cationic SCNPs are more likely to translocate into cells than neutral, anionic, or zwitterionic counterparts. For nanoparticles in the 5–15 nm range, engineering the surface charge becomes essential, as too high a positive charge (e.g., increasing the charge density to 20 mol%) can lead to deleterious effects such as excessive adhesion to the cell membrane and subsequent cell death, whereas a moderate positive charge can enhance uptake.

Nanoparticles in the 5–15 nm range, such as the ultrasmall Ru-based coordination polymer nanodots (CPNs), are discussed by Zhang et al. (2019) [27]. These nanoparticles in the 5–15 nm range can be designed for dual functions. In the work by Zhang et al. (2019) [27], the Ru-Phen CPNs are not only potent agents for photothermal therapy (PTT) but also serve as contrast agents for photoacoustic (PA) imaging. The strong near-infrared (NIR) absorption enables both deep-tissue imaging and effective light-to-heat conversion. This PA (808 nm) imaging-guided PTT (808 nm, 0.5 W/cm^2^) approach demonstrates how these nanoparticles integrate diagnostic imaging with treatment, embodying the concept of theranostics.

One example of nanoparticles with a size in the 5–15 nm range is the cerium oxide (CeO_2_) nanoparticles used in the antiviral filter study [28]. In this work, the authors report that the optimum formulation showed a particle size of 15.8 ± 1.91 nm and a zeta potential of –14 ± 0.14 mV. Nanoparticles of this size are particularly significant because their small dimensions lead to a very high surface-area-to-volume ratio. In the study, such properties were harnessed by incorporating the CeO_2_ nanoparticles into polyacrylonitrile nanofibers through an electrospinning process. The findings indicate that the nanoscale features, demonstrated by scanning electron microscopy SEM imaging, even after the incorporation of CeO_2_ nanoparticles, play an important role in the performance of the antiviral barrier. This substantial surface area can enhance interactions with viral particles, contributing to the filter’s ability to prevent viral entry into the host cells as well as prevent their replication inside the cells via adsorption and virucidal antiviral mechanisms.

#### 2.1.2. Dendrimers

Dendrimers represent a unique class of hyperbranched, monodisperse macromolecules that can inherently achieve sizes below 10 nm in diameter. Poly(amidoamine) (PAMAM) dendrimers are the most extensively studied and exhibit well-defined generations (G1–G10), where higher generations lead to increased size and surface functionality [29]. For drug delivery applications, lower-generation PAMAM dendrimers (G3–G5) can be employed to achieve total particle sizes in the 5–15 nm range, making them ideal for sub-15 nm formulation strategies [30].

The highly branched architecture of dendrimers allows for multivalent drug conjugation, surface PEGylation, or ligand attachment to enable tumor targeting. Both covalent and non-covalent drug loading strategies are used—hydrophobic drugs can be entrapped within the interior cavities, while hydrophilic drugs and biomolecules (e.g., siRNA, peptides) can be complexed or conjugated to the terminal groups [31]. Recent studies have demonstrated that doxorubicin-loaded PAMAM dendrimers functionalized with targeting ligands (e.g., folate, RGD peptides) exhibit enhanced tumor accumulation and deep penetration in 3D tumor spheroids compared to larger polymeric carriers [32].

Liu et al. (2021) [33] developed multifunctional nanoparticles with a mean size of 11.61 nm. The nanoparticles are constructed as core–shell tecto dendrimers (CSTDs) that incorporate gold nanoparticles (AuNPs) within a dendritic structure. The size (11.61 nm) and the multifunctionality of these nanoparticles enable precise tumor imaging through dual CT/MR modalities, which is crucial for accurate diagnosis of cancers that overexpress specific integrins such as αvβ3. The interdisciplinary approach—combining materials chemistry, supramolecular assembly, and biomedical imaging—demonstrates a promising strategy for constructing nanoscale contrast agents that are effective in clinical imaging applications.

One example of nanoparticles operating at a size near 15 nm is the enzyme-responsive nanogel carrier (NG-1) when loaded with doxorubicin (DOX) and exposed to elastase. Although the nanocarrier was originally constructed from PAMAM dendrimers as follows:

G4 PAMAM dendrimer molecules were functionalized (modified via covalently conjugating RGDC, RAADyC, and PEG chains on the periphery) and were then crosslinked by an oxidation reaction (using NaIO_4_ to initiate the chemical crosslink of the functional groups on the periphery) to form the nanogel carrier (NG-1) [34].

Initially, the sizes were measured by transmission electron microscopy(TEM), SEM, and dynamic light scattering (DLS) as 20 nm for Mac-1 (the modified dendrimer) and 50 nm for NG-1 (the crosslinked nanogel). However, after embedding DOX into NG-1 and testing in the presence of elastase, the size of NG-1 with embedded doxorubicin hydrochloride (DOX) decreased significantly to 15 nm in the presence of elastase. This reduction indicates that the enzyme triggers decomposition of the nanogel, which in turn leads to a sustained release of the drug [34].

An example of nanoparticles in the 5–15 nm range is the CD44-targeted dendrimer formulation described in the study by Kesarwani et al. [35]. In this work, the authors engineered a nanosystem using an amine-terminated fourth-generation PAMAM dendrimer that was conjugated with hyaluronic acid (HA) to yield a targeted construct for pancreatic cancer therapy. The resulting dendrimer nanosystem (referred to as HA-PAMAM-CDF) had a particle size and surface charge of 9.3 ± 1.5 nm and −7.02 ± 9.53 mV, respectively. This particle size, falling well within the 5–15 nm range, is significant because nanoscale dimensions can favor enhanced tissue penetration and cellular uptake.

Nanoparticles in the 5–15 nm range were designed to have molecular precision with well-defined particle sizes, bright and stable fluorescence, and the ability to be tailored for specific biomedical applications. In the study by Yang et al. (2022) [36], the authors report cyanine nanodots that exhibit these properties by employing a divergent synthesis of cyanine-dye-cored polylysine dendrimers. This synthesis approach leads to a single-molecule structure, well-defined particle size, customizable fluorescent spectrum, and bright and stable fluorescence [36].

According to Tomalia et al. (2007) [37], dendrimers exemplify nanoparticles that can be precisely engineered within the 5–15 nm range. They note that these are routinely synthesized as tunable nanostructures that may be designed and regulated as a function of their size, shape, surface chemistry, and interior void space. Such precise nanoscale scaffolding—comparable to traditional biomacromolecules like DNA/RNA or proteins—enables these particles to serve dual functions in nanomedicine.

One highlighted example is the STARBURST PAMAM dendrimer prototype. This lead candidate, described as [core: 1,4-diaminobutane; G(generation) = 4.5, dendri-PAMAM(CO_2_Na)], has an approximate diameter of 5.0 nm. It was selected for further study based on its very favorable biocompatibility profile as determined by the Nanotechnology Characterization Laboratory (an affiliate of the National Cancer Institute), which found the compound to be benign, non-immunogenic, and highly biocompatible. Such biocompatibility is a major asset for both diagnostic and therapeutic applications.

In terms of applications, nanoparticles engineered within this size range are particularly suited for:

Targeted diagnostic imaging: The design specifics of these dendrimers allow them to be modified as targeted, diagnostic MRI (magnetic resonance imaging)/NIR (near-IR) contrast agents. This precise formulation enhances imaging quality while helping to direct the contrast agent to specific tissues.

Drug delivery: Their nanocontainer properties and adjustable surface chemistries make them ideal carriers for therapeutic agents. This controlled delivery can improve the efficacy of cancer therapies by enabling targeted delivery and controlled release.

Physiological clearance: The expected desirable mammalian kidney excretion properties are crucial for reducing long-term toxicity. Nanoparticles in the lower end of the 5–15 nm range often have such advantages, ensuring that compounds do not accumulate in unintended organs.

The overall significance is that nanoparticles like these dendrimers, due to their tunable structural features, offer both diagnostic and therapeutic benefits. Their precise nanoscale size fosters improved targeting and bio-distribution, which is key to the emerging field of nanomedicine.

Despite their advantages, dendrimers face challenges such as potential cytotoxicity due to surface amines, limited drug loading capacity, and scalability of synthesis. Nevertheless, surface modification with PEG, acetyl groups, or zwitterionic moieties has been shown to reduce toxicity and improve circulation half-life [38].

#### 2.1.3. Comparative Considerations

While both polymeric micelles and dendrimers can achieve sub-15 nm sizes, their mechanisms of drug loading, release, and degradation differ substantially. Polymeric micelles typically disassemble under dilution or biological conditions, while dendrimers offer more stable architectures. The choice between linear polymer-based nanoparticles and dendrimers depends on the therapeutic payload, required release profile, and targeted delivery strategy. Notably, both systems are compatible with functionalization approaches for imaging, stimuli-responsiveness, and active targeting, making them attractive candidates for multifunctional cancer nanotherapeutics.

For hydrophobic small molecules, polymeric micelles generally offer higher effective loading at sub-15 nm via core solubilization and are ideal when rapid systemic distribution and deep tumor penetration are prioritized. Their liabilities are dilution-induced disassembly below the critical micellar concentration (CMC) and potential payload burst. The payload burst can be mitigated by core-crosslinking, π-π stacking motifs, or mixed micellization. Dendrimers handle small molecules via covalent conjugation or interior entrapment; while loading is typically lower, release is programmable via cleavable linkers with greater stability against dilution. For nucleic acids (siRNA/mRNA/ASO), dendrimers (amine-terminated) offer efficient electrostatic complexation and multivalent targeting but require careful surface masking/PEGylation to minimize amine-driven cytotoxicity.

Polymeric micelles benefit from self-assembly in scalable unit operations (microfluidics, ethanol injection) with familiar excipients; dendrimers require precision macromolecular synthesis and rigorous batch-to-batch architecture control.

### 2.2. Lipid-Based Nanoparticles Below 15 nm in Size for Cancer Therapy

Lipid-based nanoparticles have become prominent carriers in nanomedicine due to their high biocompatibility, ability to encapsulate a broad range of drugs, and versatility in structural design. Recent advances in formulation strategies and lipid chemistry have enabled the development of lipid-based nanoparticles with hydrodynamic sizes under 15 nm, offering improved tumor penetration, faster diffusion through dense extracellular matrices, and, in some cases, the potential for renal clearance to minimize off-target accumulation.

#### 2.2.1. Lipid Nanoparticles (LNPs)

LNPs are a class of colloidal carriers typically composed of ionizable lipids, phospholipids, cholesterol, and PEG-lipids. Originally developed for nucleic acid delivery, LNPs have recently gained major attention following their use in mRNA vaccines. Their tunable composition allows for size control, and particle sizes <15 nm have been achieved using microfluidic mixing, high-shear homogenization, and ethanol injection methods [39].

In cancer therapy, LNPs < 15 nm are being investigated for the delivery of small-interfering RNA (siRNA), mRNA, and small molecules. Their small size enables deep tumor penetration, especially in poorly vascularized solid tumors. Ionizable lipids facilitate endosomal escape, making these carriers highly effective for intracellular delivery [40]. However, sub-20 nm LNPs require careful balancing of lipid composition, as excessive PEGylation can reduce cellular uptake and endosomal release efficiency [41].

One type of nanoparticle in the 5–15 nm range is the nanolipoprotein particle (NLP) described by Scharadin et al. (2017) [42]. These NLPs are ~10 nm in diameter discoidal cell membrane mimics composed of apolipoproteins surrounding a lipid bilayer. This NLP of ~10 nm offers a highly promising avenue for research into membrane protein biology and pharmacological screening. Their ability to mimic cell membranes while preserving native protein function makes them a valuable tool for both structural biology and drug discovery.

One example of nanoparticles in the range of 5–15 nm is the sterically stabilized mixed phospholipid nanomicelle (SSMM) system used for paclitaxel delivery. In the study by Onyüksel et al. (2009) [43], the SSMM had a size of approximately 15 nm and was designed to be biocompatible and biodegradable. Nanoparticles in the range of 5–15 nm—exemplified by the SSMM used in this study—offer several advantages for targeted cancer therapy. Their small size facilitates enhanced tumor penetration, they can be engineered to be biocompatible and biodegradable, and their surface can be functionalized with targeting ligands to overcome drug resistance, thereby increasing the cytotoxic effect of the encapsulated drugs.

The nLDL particles were constructed by combining a synthetic peptide containing a lipid binding motif and the LDL receptor (LDLR) binding domain of apolipoprotein B-100 with a lipid emulsion consisting of phosphatidyl choline, triolein, and cholesteryl oleate. This composition was designed to mimic aspects of natural LDL particles while providing the structural and functional stability required for drug delivery purposes. The synthetic nLDL particles show promise as targeted drug delivery vehicles due to their reproducible size (approximately 10.5 nm), specific receptor-mediated uptake, and effective intracellular trafficking to lysosomes. These characteristics make them strong candidates for future therapeutic strategies in treating GBM tumors (Nikanjam et al., 2007) [44].

According to Choi et al. (2018) [45], one promising approach for designing nanoparticles in the 5–15 nm range is to use a biomimetic nano-surfactant that stabilizes very small phospholipid assemblies. In their work, the authors describe how the sterilized 18-amino-acid biomimetic of the amphipathic helical motif abundant in HDL-apolipoproteins plays a crucial role in inducing a nanoscale phase (glass) transition in the phospholipid monolayer. This transition allows the formation of 5–7 nm phospholipid micelles that are stable even at high volume fractions. The work highlights a method to create sub-15 nm nanoparticles that combine high drug-loading capacity with improved tumor penetration and favorable biological distribution, making them a strong candidate for next-generation nanomedicine strategies.

#### 2.2.2. Liposomes

Liposomes are spherical vesicles with one or more lipid bilayers surrounding an aqueous core. While conventional liposomes often range between 50 and 200 nm, downsizing to <20 nm has been achieved using techniques such as controlled extrusion, detergent dialysis, and ethanol injection with optimized lipid-to-drug ratios [46]. Small unilamellar vesicles (SUVs) with diameters of 15–20 nm have demonstrated superior penetration in tumor spheroids and dense stromal tissues compared to larger vesicles.

The reduced size facilitates enhanced diffusion through the extracellular matrix and better interstitial distribution. However, liposomes <15 nm may suffer from reduced drug loading due to smaller internal volumes and increased membrane curvature stress. Surface modifications with PEG, antibodies, or peptides allow active targeting and prolonged circulation [47].

#### 2.2.3. Nanoemulsions

Nanoemulsions are kinetically stable oil-in-water (O/W) or water-in-oil (W/O) emulsions stabilized by surfactants. Their droplet sizes typically range from 20 to 200 nm, but ultra-fine nanoemulsions (<15 nm) have been produced using high-pressure homogenization or spontaneous emulsification techniques [48].

For cancer therapy, sub-15 nm nanoemulsions offer an excellent platform for solubilizing poorly water-soluble anticancer drugs such as paclitaxel, docetaxel, and curcumin. These formulations benefit from high surface area, fast cellular uptake, and potential for intravenous administration without the use of harmful solvents like Cremophor EL. Incorporating tumor-targeting ligands and stimuli-responsive components further enhances therapeutic efficacy [49].

One example of nanoparticles in the 5–15 nm range is given by the transferrin-modified, multi-component paclitaxel (PTX)–loaded β-elemene nanoemulsion (Tf-PE-MEs) described by Chen et al. (2024) [50]. The Tf-PE-MEs were reported to have a particle size of 14.87 ± 1.84 nm. Such small sizes are often associated with enhanced tissue penetration and improved delivery to target sites. The nanoemulsion was modified with transferrin, a ligand that targets cells overexpressing transferrin receptors (TfR). Through transferrin modification, Tf-PE-MEs accumulated at the tumor site efficiently with overexpressed transferrin receptor (TfR) on the surface of A549 cells. This receptor-mediated targeting is particularly advantageous for cancer treatment because it can concentrate the therapeutic agents directly in tumor cells while potentially reducing systemic toxicity. The example provided in the context illustrates that nanoparticles in the 5–15 nm range can be engineered to combine multiple drugs with targeting ligands, resulting in a formulation that not only exhibits excellent physical characteristics (size and zeta potential) but also shows enhanced tumor accumulation, therapeutic efficacy, and reduced systemic toxicity. This strategy offers a promising approach for targeted anticancer treatment, particularly in challenging settings such as non-small-cell lung cancer.

A novel nanoemulsion formulation (PGPs-NE) [51] was developed using pomegranate polysaccharides (PGPs). One of the key physicochemical characteristics of the formulation was its average hydrodynamic particle size, which was reported to be 9.5 nm. The method used in preparing the PGPs-NE formulation resulted in a very high entrapment efficiency (92.82%). Not only does the small size facilitate enhanced drug encapsulation and delivery, but it also contributes to the nanoformulation’s potent antioxidant, anti-inflammatory, and antitumor properties compared to free PGPs. This nanoemulsion formulation offers distinct advantages in drug delivery systems, including improved stability, uniform size, high drug entrapment, and potent biological effects.

Azadi et al. (2023) [52] developed a nano-scaled emulsion that encapsulated Mentha pulegium essential oil and achieved a droplet size of 7.70 ± 1 nm. Their small size ensures enhanced penetration and cellular uptake, which is especially valuable when treating conditions such as melanoma. The study demonstrated that after applying different concentrations of the nano-scaled emulsion to human A375 melanoma cells, there was a 90 and 45% reduction in cell viability for nanogel and nano-scaled emulsion treatments, respectively, indicating significant cytotoxic effects. This efficacy was attributed to the induction of apoptosis, as confirmed by flow cytometry findings and gene expression analysis that showed an up-regulation of Bax and down-regulation of Bcl-2 genes (Azadi et al., 2023) [52].

One example of a nanoparticle formulation in the 5–15 nm range is the catechin nanoemulsion developed from oolong tea leaf waste. In this study, the authors report that the nanoemulsion was prepared using lecithin, Tween-80, and water, and it exhibited a particle size of 11.3 nm, which falls well within the range of interest (5–15 nm) (Lin et al., 2021) [53]. Functionally, these nanoparticles were evaluated against prostate cancer cells (DU-145), where they showed enhanced activity compared to extracts. Specifically, after 48 h of incubation, the nanoemulsion had a lower IC_50_ value (13.52 μg/mL) than the extracts (214.6 μg/mL), suggesting a significant improvement in efficacy when formulated at this nanoscale. In addition, both the nanoemulsions and the extracts raised the activities of caspase-8, caspase-9, and caspase-3, and induced cell cycle arrest at the S and G2/M phases—all key markers of apoptosis in cancer cells. In vivo, treatment with the nanoemulsion led to a marked reduction in tumor volume and weight, further linked to decreased serum levels of EGF and VEGF, which are critical factors in tumor growth and angiogenesis.

Resveratrol-gold nanoparticles (R-GNPs) and a resveratrol nanoemulsion were prepared from grape skin with mean particle sizes less than 15 nm. Specifically, the RGNPs were reported to have a mean particle size of 11.9 nm, and the resveratrol nanoemulsion had a mean particle size of 14.1 nm (Inbaraj et al., 2021) [54]. Both the R-GNPs and the resveratrol nanoemulsion were tested for their antiproliferative effects on pancreatic cancer cells (BxPC-3). The study reported that both can down-regulate expressions of cyclin A, cyclin B, CDK1, and CDK2 and up-regulate expressions of p53 and p21, which are key regulators of cell cycle arrest and apoptosis.

#### 2.2.4. Solid Lipid Nanoparticles (SLNs)

Solid lipid nanoparticles (SLNs) consist of solid lipids stabilized by surfactants, forming a solid lipid matrix at body temperature. While conventional SLNs are generally in the range of 50–200 nm, formulation advancements using ultrasonication, solvent evaporation, and microfluidics have enabled size reduction to ~15 nm or even lower in optimized conditions [55].

Sub-15 nm SLNs combine the advantages of liposomes and polymeric nanoparticles, offering controlled drug release, physical stability, and drug protection from degradation. In cancer applications, SLNs have been used for delivery of doxorubicin, tamoxifen, and etoposide. However, reduced particle size may lead to lower encapsulation efficiency, requiring careful optimization of lipid type, drug–lipid interactions, and surfactant concentration [56].

#### 2.2.5. Comparison of Lipid-Based Nanoparticles

Sub-15 nm LBNPs represent a promising frontier for cancer therapy, offering a balance of biocompatibility, tumor penetration, and functional flexibility. However, achieving and maintaining ultrasmall size without compromising therapeutic loading or stability remains a key formulation challenge. A comparison of lipid-based nanoparticles is shown below in Table 2.

At less than 15 nm, LNPs expel nucleic acids due to ionizable lipids and endosomal escape, but can trade uptake vs. stealth with PEG density. Liposomes at these sizes penetrate well but face curvature-limited loading; they favor small hydrophilic drugs or imaging agents and benefit from active targeting. Nanoemulsions deliver highly hydrophobic payloads with kinetic stability and simple manufacturing, while SLNs add solid-matrix protection. Selection should weigh payload polarity, required endosomal escape, dilution stability, infusion tolerability (surfactants), and desired clearance rate.

### 2.3. Metallic Nanoparticles Below 15 nm in Size for Cancer Therapy

Metallic nanoparticles (MNPs) are also a powerful tool in oncology due to their unique physicochemical properties, including tunable SPR, magnetic responsiveness, and ease of functionalization. When engineered to sub-15 nm sizes, these nanoparticles exhibit distinct biological interactions compared to their larger counterparts, including improved tumor penetration, faster cellular uptake, and altered clearance pathways. Among MNPs, gold, silver, and iron oxide nanoparticles are the most widely investigated for diagnostic and therapeutic applications in cancer.

#### 2.3.1. Gold Nanoparticles (AuNPs)

AuNPs are among the most studied metallic systems for cancer applications due to their chemical stability, biocompatibility, and surface plasmon resonance (SPR) properties. AuNPs < 15 nm have been shown to offer several advantages, including the following:

- Enhanced diffusion through tumor interstitium;
- Efficient renal clearance for ultrasmall AuNPs (<5–8 nm);
- High surface-area-to-volume ratio for functionalization with targeting ligands, drugs, and imaging agents.

The synthesis of sub-15 nm AuNPs is commonly achieved via the citrate reduction method, seed-mediated growth, or Brust–Schiffrin synthesis. Particles as small as 2–5 nm are attainable with precise control over size and monodispersity [57].

In cancer therapy, AuNPs serve as

- Drug carriers, via surface conjugation or thiol-linker attachment of doxorubicin, paclitaxel, etc.;
- Photothermal agents, where NIR laser exposure induces localized hyperthermia for tumor ablation;
- Radiosensitizers, enhancing the effect of radiation therapy by increasing local dose deposition [58].

Sub-15 nm AuNPs also demonstrate unique cellular internalization profiles, often via clathrin- or caveolae-mediated endocytosis, and can escape lysosomal degradation depending on surface modifications [59].

LSPR Tunability and NIR Advantages: Metallic nanoparticles’ optical behavior is governed by their localized surface plasmon resonance (LSPR), which can be precisely tuned by controlling particle size, geometry, and interparticle coupling. In sub-15 nm systems, the LSPR peak of spherical gold nanoparticles typically lies around 520 nm; however, increasing particle diameter or introducing anisotropic shapes (e.g., nanorods, nanoshells, or nanostars) induces a red-shift of the absorption band into the near-infrared (NIR) window (650–900 nm). This spectral shift is particularly valuable for biomedical applications because NIR light penetrates deeper through tissue with minimal scattering and absorption, allowing efficient in vivo photothermal conversion and photoacoustic imaging. For example, Au nanorods or ~10 nm AuNP clusters with elongated aspect ratios exhibit strong NIR absorption, enabling localized heating and enhanced tumor ablation efficiency under 808 nm laser irradiation. Thus, the ability to engineer LSPR wavelength via nanoparticle design provides a versatile handle to balance optical efficiency and tissue penetration in metal-based theranostic platforms.

Bao et al., 2016 [60] investigated AuNPs conjugated with 10-hydroxycamptothecin (HCPT) and prepared a series of particles with mean diameters of approximately 10, 25, and 50 nm. An in vitro drug release study of the HCPT conjugated to the AuNPs demonstrated that HCPT was continuously released for 120 h. This sustained release profile suggests that nanoparticles around 10 nm can serve as efficient drug delivery scaffolds, allowing for prolonged drug availability at the targeted site.

Steckiewicz et al. (2019) [61] evaluated AuNPs of different sizes and shapes. These AuNPs spheres have an approximate size of 6.3 nm, which places them well within the 5–15 nm range. The authors noted that AuNPs spheres are the safest ones compared to the larger rod-shaped (≈39 nm in length and 18 nm in width) and star-shaped (≈215 nm) nanoparticles, although they possessed a small anticancer potential.

In the study by Selim et al. [62], the AuNPs were engineered to have an optimal particle size of 9 nm diameter for AuNPs. This size range is particularly useful because it can enhance tumor targeting and improve biodistribution. The nanoparticles were produced using a chemical reduction method to formulate compound 3-citrateAuNPs. They were evaluated in tumor-bearing mice, and intravenous administration resulted in effective tumor accumulation. This is significant because nanoparticles of this size can overcome biological barriers and localize within target tissues, thereby enhancing their therapeutic potential.

One example of nanoparticles in the 5–15 nm range is represented by the spherical AuNPs prepared using a green synthesis approach. In this work, the authors prepared AuNPs stabilized and capped with a modified pullulan derivative (PABA-QP) that produced particles with a narrow size distribution of 13.7 ± 1.9 nm (Laksee et al., 2018) [63]. Their size in the 5–15 nm range is particularly relevant because nanoparticles of this scale typically offer a high surface-to-volume ratio. This characteristic facilitates efficient drug loading (in this case, uptake of doxorubicin (DOX) was achieved via intermolecular interactions with high drug loading). Additionally, such nanoparticles enhance cellular uptake by endocytosis. The authors reported that the DOX-loaded AuNPs (DOX-AuNPs@PABA-QP) demonstrated significantly higher intracellular uptake, which contributed to enhanced cytotoxicity against cancer cells. Notably, DOX-AuNPs@PABA-QP (IC_50_ = 0.39 μM) showed a 2.1-fold higher cytotoxicity against Chago cells than DOX alone (IC_50_ = 0.82 μM) while still exhibiting reduced toxicity against normal cells (Wi-38).

Venkatpurwar et al. (2011) [64] demonstrated the design and application of AuNPs with sizes that fall within the range of approximately 5–15 nm. In this study, the authors report that the prepared AuNPs showed SPR centered at 520 nm with an average particle size of 13 ± 5 nm. This indicates that by controlling the synthesis conditions, nanoparticles with sizes largely confined within the target range can be obtained.

The study further explores the potential of these nanoparticles as drug carriers by conjugating them with doxorubicin hydrochloride (DOX). The mechanism of drug conjugation is attributed to hydrogen bonding; as stated, spectroscopic examination revealed that DOX conjugated onto AuNPs via hydrogen bonding. Moreover, there is a significant pH-dependent release, where the release of DOX from DOX-loaded AuNPs was found to be sixfold higher in acetate buffer (pH 4.5) as compared to physiological buffer (pH 7.4). This property is particularly useful for targeted anticancer therapy since tumor microenvironments often have a lower pH.

In vitro studies demonstrated that these AuNPs exhibit enhanced cytotoxicity on the human glioma cell line (LN-229) compared to native porphyran. Furthermore, the DOX-loaded AuNPs demonstrated higher cytotoxicity on the LN-229 cell line as compared with an equal dose of native DOX solution. This enhancement likely stems from the efficient cellular uptake and controlled release properties imparted by the nanoparticle carrier system.

An example of noble metal nanoparticles of different sizes and shapes combined with conjugated functional polymers that give rise to advanced core–shell hybrids with interesting physical characteristics and potential applications in sensors or cancer therapy was described by Fratoddi et al. in 2011 [65]. Their paper specifies that the mean diameter of the metal core is about 10–30 nm, with a polymeric shell of about 2 nm. The nanoparticles in the lower portion of this size interval (approximately 10 nm) would fall within or close to a 5–15 nm range.

The paper directly reports metal cores with diameters of 10–30 nm; nanoparticles in the range of 5–15 nm—especially those near 10 nm—would share many of the beneficial properties described. They demonstrate the feasibility of forming core–shell hybrids with tunable surface properties via polymer coatings, and they possess nanoscale-induced physical characteristics that can be tailored for applications such as high-sensitivity sensors or targeted cancer therapy.

Jana et al. (2021) [66] described nanodrugs that include 2 nm core AuNPs covered completely with multivalent hydrocarbon chains to a final diameter of ~10 nm as single drug molecules. Nanoparticles sized in the 5–15 nm range, as exemplified by the system described by Jana et al., offer a promising platform for cancer therapy. They combine the benefits of nanoscale dimensions for optimal cellular interaction with engineered surface properties, leading to enhanced bioactivity such as increased ROS generation and induction of apoptosis, all while potentially circumventing traditional drug resistance mechanisms.

Based on Janic et al. (2021) [67], AuNPs in the size range approaching 14 nm have been shown to offer promising advantages for cancer radiotherapy and immunomodulation in triple-negative breast cancer (TNBC).

According to the study, AuNPs are able to enhance the local absorption of radiation energy within tumors while sparing surrounding healthy tissue. As stated in the paper, AuNPs have been shown to enhance cancer radiotherapy (RT) gain by localizing the absorption of radiation energy in the tumor while sparing surrounding normal tissue from radiation toxicity. This selective localization allows for an increase in radiation-induced DNA damage within the tumor.

The paper by Setyawati et al. [68] specifically emphasizes the 10–30 nm window for demonstrating nanoparticle-induced endothelial leakiness (NanoEL). In this study, the nanoparticle’s size was identified as a key determinant in their ability to induce micrometer-sized gaps between endothelial cells. This process, called NanoEL, facilitates access for systemically administered materials, including nanomedicine, to underlying tissues such as tumors, even in settings where the conventional endothelial permeability and retention (EPR) effect is weak or absent.

In the study by Mardina et al. [69], the authors used a green synthesis approach where aqueous extracts from the plant’s leaves were prepared by heating with distilled water at 60 °C for 30 min. This leaf extract was then mixed with HAuCl_4_ and further heated (also at 60 °C for 30 min) to produce AuNPs-ALSt. Notably, these AuNPs had an average particle diameter of 11.86 ± 3.37 nm. These AuNPs with sizes in the 5–15 nm range, as illustrated by the AuNPs-ALSt with an average of 11.86 nm, are produced via a sustainable synthesis route using aqueous plant extracts. These nanoparticles are characterized by their good stability, controlled size distribution, and promising biological activities, making them attractive for further research in biomedicine and related fields.

#### 2.3.2. Silver Nanoparticles (AgNPs)

AgNPs have potent cytotoxic and antimicrobial properties, making them attractive for anticancer applications. At sizes below 15 nm, AgNPs exhibit increased surface reactivity and ion release, contributing to their therapeutic efficacy. However, their application requires careful control due to potential systemic toxicity.

Mechanisms of anticancer action include:

- Induction of reactive oxygen species (ROS)
- DNA damage and mitochondrial disruption
- Cell cycle arrest and apoptosis in various cancer cell lines [70,71]

Green synthesis methods using plant extracts or biopolymers have enabled the preparation of AgNPs < 15 nm with reduced toxicity and increased biocompatibility. These particles are also being integrated with polymers or lipids to improve stability and reduce Ag^+^ release rate [72].

The AgNPs (referred to as WcAgNPs) were synthesized by reducing a silver nitrate solution using an in vitro grown leaf extract from the anti-diabetic medicinal plant *Withania coagulans*. This synthesis approach is described as green and cost-effective. The WcAgNPs were ~14 nm in size, having a spherical shape with a face-centered cubic structure (Tripathi et al., 2019) [73]. The WcAgNPs demonstrated enhanced antioxidative potential when compared with the *Withania coagulans* leaf extract. This suggests that reducing the size to the nanoscale range can improve the bioactivity of compounds. Regarding anticancer activity, the nanoparticles showed cytotoxicity against SiHa (cervical cancerous, hyper-triploid) cell lines. After a 48 h incubation, apoptotic effects were observed after 48 h incubation with 13.74 μg·mL^−1^ (IC_50_) concentration of WcAgNPs.

The study by Xia et al. [74] describes a green synthesis method in which plant constituents from *Taxus yunnanensis* callus extracts serve as both reducing and capping agents. These phytochemicals are suggested, by Fourier transform infrared spectroscopy (FTIR), to play important roles in the formation and stabilization of AgNPs. Such green approaches are noted for being both environmentally friendly and cost-effective. Furthermore, the biosynthesized AgNPs demonstrated stronger cytotoxic activity against human hepatoma SMMC-7721 cells and induced noticeable apoptosis in SMMC-7721 cells, while having lower cytotoxicity toward normal human liver cells (HL-7702). This selective cytotoxicity makes nanoparticles in this size range attractive candidates for potential anticancer therapeutics. Their small size ensures that they can interact efficiently at the cellular level, often leading to enhanced delivery of the active silver species and the induction of biological responses that facilitate both antibacterial and anticancer effects.

One example of nanoparticles in the 5–15 nm range is the green-synthesized AgNPs described by Majeed et al. (2022) [75]. As noted in the report, TEM revealed that AgNPs are spherical and highly scattered and vary in size from 7.18 nm to 13.24 nm. The AgNPs exhibited excellent antibacterial activity and a strong synergistic effect against methicillin-resistant bacteria, Staphylococcus aureus (MRSA) ATCC-4330 and Streptococcus epidermis (MRSE) ATCC-51625. This suggests that nanoparticles of this size can disrupt bacterial processes effectively, possibly due to their high surface-area-to-volume ratio.

For potential applications in diabetes management, the nanoparticles were assessed for antidiabetic activity. The assays revealed inhibitory effects on key enzymes with an IC_50_ for alpha-amylase of 428.60 μg/mL and an IC_50_ for alpha-glucosidase of 562.02 μg/mL. Furthermore, flow cytometry analysis on Hep-2 liver cancer cells treated with 40 μg/mL AgNPs showed increased expression of 2-NBDG, a glucose analog used to monitor glucose uptake, indicating enhanced cellular glucose absorption compared to control using metformin.

The nanoparticles in the range of 5–15 nm—such as the 10 nm AgNPs studied—exhibit several notable characteristics that make them attractive for biomedical applications. In the study cited, AgNPs were investigated for their effects on primary rat hepatic stellate cells (HSCs), a cell type critical to liver fibrosis and cirrhosis. As noted in the article, AgNPs inhibited the proliferation of HSCs and induced their apoptosis in a size- and dose-dependent manner (Sun et al., 2013) [76].

One study used mycelial filtrate of Aspergillus terreus BA6 to synthesize silver nanoparticles (AgNPs) whose size distribution was characterized among other properties. The study reports that the microscopic results showed particles with a narrow size distribution ranging from 7 to 23 nm (Lotfy et al., 2021) [77]. The AgNPs microfabricated by A. terreus demonstrated relevant biological activities. The study detailed potent minimum inhibitory concentration (MIC) and broad minimum bactericidal/fungicidal concentration (MBC/MFC) values against 12 reference microorganisms. Such results indicate that nanoparticles with dimensions in a more confined range, like 5–15 nm, can be very effective in antimicrobial applications; smaller nanoparticles generally offer improved interaction with microbial cells due to their increased surface area. Additionally, in vitro cytotoxicity assays showed significant antitumor activity; the AgNPs inhibited adenocarcinoma epithelial cells (Mcf-7 cell line) with an IC_50_ of 87.5 μg/mL.

Despite their promise, sub-15 nm AgNPs face challenges in clinical translation due to concerns over accumulation, oxidative stress, and non-specific toxicity. Hence, targeted delivery systems and surface passivation strategies are key to improving their safety profile.

#### 2.3.3. Iron Oxide Nanoparticles

Iron oxide nanoparticles, particularly superparamagnetic iron oxide nanoparticles (SPIONs), are widely used in cancer diagnosis and therapy. Particles < 15 nm in diameter, including ultrasmall SPIONs (USPIONs), show significantly improved circulation time and tumor accumulation due to their favorable pharmacokinetics and ability to avoid uptake by the reticuloendothelial system (RES) [78].

Moiseeva et al. 2025 [79] details the synthesis of ultrasmall maghemite nanoparticles as an MRI contrast agent and also describes the unique combination of aggregation stability, low toxicity, and tumor visualization of these ultrasmall iron oxide nanoparticles.

SPIONs < 15 nm are commonly synthesized via co-precipitation or thermal decomposition and coated with stabilizing agents such as dextran, PEG, citrate, or oleic acid. Their applications in oncology include:

- Magnetic resonance imaging (MRI) contrast agents, especially T1-weighted imaging, for early tumor detection;
- Hyperthermia therapy, using alternating magnetic fields to generate localized heat;
- Targeted drug delivery, when functionalized with anticancer drugs, peptides, or antibodies [80].

Ultrasmall iron oxide nanoparticles also exhibit enzyme-mimicking (nanozyme) activity, generating ROS in the tumor microenvironment, which can be harnessed for chemodynamic therapy (CDT) [81]. Biodegradation into iron ions further supports their biocompatibility and excretion through natural iron metabolic pathways.

In the study described by Tian X et al. (2022) [82], iron oxide nanoparticles were synthesized and evaluated with sizes spanning from 2 to 100 nm, and the results highlight some important points about nanoparticles that fall within the 5–15 nm range—especially those around 10 nm.

According to the report, ultrasmall nanoparticles (<~5 nm) could accumulate in the nucleus and were more efficient in triggering the generation of ^•^OH than larger nanoparticles due to the quicker release of Fe^2+^. This finding indicates that particle size directly affects both cellular uptake and the mechanism of cytotoxicity via ferroptosis. However, these very small nanoparticles may not always result in the best therapeutic outcome when considering overall in vivo performance.

In fact, the study notes that the 10 nm iron oxide nanoparticles group displayed the best antitumor effect in vivo. Nanoparticles in the 5–15 nm range, and particularly around 10 nm, seem to strike an optimal balance. They are likely large enough to achieve favorable biodistribution—and hence sufficient tumoral accumulation and intratumoral distribution—while still maintaining the ability to release Fe^2+^ in a manner that efficiently triggers ferroptosis. This balance is critical, considering that both too small and too large nanoparticles can have limitations relating to biodistribution, cellular uptake, and therapeutic efficacy.

Thus, nanoparticles in the range of 5–15 nm, as informed by the context, would emphasize that these particles:

- Benefit from an optimal size that supports effective tumor targeting and in situ distribution.
- Maintain a favorable balance between releasing therapeutic agents (Fe^2+^ for triggering ^•^OH generation) and achieving accumulation within the tumor.
- Exhibit a therapeutic profile that, at least in the case of the 10 nm particles, leads to the best antitumor effect in vivo compared to smaller (e.g., <~5 nm) or larger nanoparticles.

This information suggests that when designing nanoparticles for cancer therapy via ferroptosis, selecting a particle size within the 5–15 nm range—especially around 10 nm—might offer the most promising clinical benefits by harnessing both efficient intracellular effects and optimal biodistribution characteristics.

In one study, researchers synthesized gelatin-stabilized iron oxide nanoclusters having a primary crystallite size of ~10 nm (Yadav et al., 2022) [83]. These nanoparticles were further functionalized on the surface with indocyanine green (ICG)–bound albumin-stabilized gold nanoclusters to form what were termed Prot-IONs.

- The nanoparticles display a superparamagnetic nature which imparts a high relaxivity of ~225 mM^−1^·s^−1^ for T_2_-weighted magnetic resonance imaging.
- They are also amenable as contrast agents in photoacoustic and NIR imaging, enhancing their utility as theranostic agents.

Thus, nanoparticles in the 5–15 nm range—as exemplified by the Prot-IONs—can be comprehensively engineered to combine therapeutic modalities such as photothermal therapy and radiotherapy with diagnostic imaging. Their size enables not only effective cellular interactions and enhanced imaging contrast but also ensures that they are bioeliminable, reducing potential long-term toxicity. This multifunctional design addresses both treatment and imaging challenges in oncology, making them a promising platform for future clinical applications.

The study by Khaniabadi et al. (2020) [84] investigated theranostic nanoparticles that specifically fall within a narrow size range. Although the paper focuses on a novel agent— trastuzumab conjugated porphyrin-superparamagnetic iron oxide nanoparticle (ION-PP-TZ)—the characterization data reveals key aspects of nanoparticles in the 5–15 nm size range. The report states that the sizes of monodispersed nanoparticles were measured in the range of 5.74–7.17 nm. The synthesis method employed allowed for tight control over the nanoparticle size distribution. Nanoparticles in this range are often preferred in biomedical applications due to their favorable surface-to-volume ratio and ability to navigate biological environments.

The theranostic potential of these nanoparticles lies in their dual functionality:

As magnetic resonance imaging (MRI) contrast agents: The study measured a transverse relaxation (r_2_) value of 52.32 mM^−1^·s^−1^ for the protoporphyrin-conjugated IONs, which is a critical parameter for effective T2 MRI contrast enhancement.

For use in photothermal therapy (PTT): Upon in vitro laser irradiation (808 nm, 200 mW), the ION-PP-TZ nanoparticles achieved a 74% MCF 7 cell reduction after 10 min at the highest Fe concentration (1.00 mg Fe/mL). This demonstrates that nanoparticles in this size range can reach the necessary thermal thresholds to induce cell ablation in targeted cancer cells while remaining non-toxic under given conditions.

An important consideration highlighted is that no cytotoxicity was observed after incubating MCF-7 cells under various Fe concentrations of nanoparticles and theranostic agents. Nanoparticles in the 5–15 nm range often have enhanced surface reactivity, but they must be carefully designed and coated (as with oleylamine and subsequent ligands in this case) to ensure biocompatibility for in vivo applications.

One example of nanoparticles in the 5–15 nm range is the Fe_3_O_4_-based system developed by Song et al., 2019 [85] for drug delivery in ovarian cancer cells. In this study, the as-synthesized NPs@carboplatin nanoparticles exhibit an average diameter of 7.88 nm. The Fe_3_O_4_ nanoparticles are used as a carrier for carboplatin, thereby forming a system (NPs@carboplatin) that enhances the cytotoxic effect compared to carboplatin alone. According to the study, this nanoformulation was effective in vitro, demonstrating higher cytotoxicity in both cisplatin-sensitive (A2780) and cisplatin-resistant (A2780DDP) ovarian cancer cell lines. The enhanced delivery is explained by the nanoparticles’ ability to be effectively taken up by ovarian cancer cell lines through an endocytosis process.

The magnetic properties of the Fe_3_O_4_ core provide an additional advantage. Under an external magnetic field, the nanocarrier can be directed to the tumor site, which aids in visualizing the tumor location and promoting subsequent antitumor activity. In vivo studies further confirmed that NPs@carboplatin can distribute into major organs, yet they demonstrated a relatively high tumor inhibition rate without obvious toxicity to normal organs.

The work emphasizes that the nanoparticle system not only enhances drug delivery but also helps to overcome platinum-based drug resistance. The improved intracellular accumulation of the drug due to the nanoparticle carrier results in enhanced cytotoxic responses, even in cancer cells that display resistance to conventional platinum chemotherapy.

#### 2.3.4. Comparative Considerations Between Metallic Nanoparticles

Hybrid Metal–Polymer Nanoconstructs for Multimodal Therapy: Beyond monocomponent metal nanoparticles, increasing attention has been devoted to hybrid architectures that integrate a metallic core with polymeric or supramolecular coatings to achieve multitherapeutic synergy. Among these, cyclodextrin (CD)-decorated metal nanoparticles represent a particularly versatile platform. The CD corona provides guest–host cavities that can encapsulate hydrophobic chemotherapeutics (e.g., doxorubicin, paclitaxel) while simultaneously allowing surface conjugation of targeting ligands such as RGD peptides or folic acid. The hybrid design enables stimuli-responsive release—for example, pH-triggered drug desorption in the acidic tumor microenvironment or NIR-induced release through localized photothermal heating of the metal core. As demonstrated in recent studies [86], Au@CD constructs can seamlessly combine photothermal therapy (PTT) with chemotherapy or photodynamic therapy (PDT) on a single nanoscale scaffold. The polymeric CD shell further improves colloidal stability, biocompatibility, and circulation half-life, while the metal core provides tunable optical absorption for deep-tissue NIR activation. Such hybrid sub-15 nm nanoconstructs exemplify how rational integration of metallic and organic components can maximize therapeutic versatility without compromising nanoscale precision. Table 3 compares different metallic nanoparticles of less than 15 nm in size.

AuNPs (1–15 nm) best fit photochemical/radiosensitization and ligand-dense theranostics. Fe_3_O_4_ (5–15 nm) nanoparticles are preferred for MRI/hyperthermia and magnetically guided delivery/chemodynamic therapy with biodegradable iron pathways. Ag (2–15 nm) provides direct cytotoxic/antimicrobial effects but demands stringent control of ion release.

Sub-15 nm metallic nanoparticles hold potential for multimodal cancer therapy. Their small size facilitates passive and active targeting, yet clinical translation demands careful control over their physicochemical properties, long-term biodistribution, and clearance mechanisms. With ongoing advancements in surface engineering and hybrid designs, metallic NPs are expected to play a critical role in next-generation cancer nanomedicine.

A comparative analysis of physicochemical and biological trade-offs between sub-15 nm and 15–30 nm nanoparticles is provided in Table 4. The smaller domain (<15 nm) favors rapid diffusion and clearance, whereas the 15–30 nm range optimizes drug loading and systemic retention.

### 2.4. Quantum Dots and Carbon-Based Nanoparticles Below 15 nm in Size for Cancer Therapy

Quantum dots (QDs) and carbon-based nanoparticles represent a unique class of nanomaterials that leverage quantum confinement, optical tunability, and high surface-to-volume ratios for multifunctional applications in cancer diagnosis and therapy. Their ultrasmall sizes—typically below 10 nm for QDs and <15 nm for certain carbon allotropes—enable rapid cellular internalization, precise tumor imaging, and, in some cases, efficient therapeutic payload delivery. Among these, graphene quantum dots (GQDs) and carbon nanotubes (CNTs) have shown significant potential due to their distinctive physicochemical and biological properties.

Nanoparticles such as semiconductor quantum dots (QDs) that fall in or near the 5–15 nm range possess several unique and useful properties that arise mainly from quantum confinement effects. A key point noted is that quantum dots (QDs) are semiconductor nanocrystals 2 to 10 nm in diameter (Zhang, et al., 2008) [87]. Although the cited size range is 2–10 nm, many nanoparticles used in biological applications are engineered within the broader range of 5–15 nm. In this size regime, the following features are characteristic:

Quantum Confinement and Tunable Optical Properties:

When the dimensions of a nanoparticle approach its Bohr radius (typically 1–10 nm for many semiconductors), discrete energy levels appear much like in a particle-in-the-box system. This quantization causes the band gap—the energy difference between the valence and conduction bands—to depend strongly on the nanoparticle size.

For example, the emission wavelength is 550 nm for 3 nm CdSe QDs and 650 nm for 7 nm CdSe QDs. In a range of 5–15 nm, nanoparticles can be tuned in size to yield desired optical emissions, an attribute that is exploited for imaging applications.

(a) High Surface-Area-to-Volume Ratio:

Nanoparticles in this range have a large proportion of atoms at or near the surface. This high surface-area-to-volume ratio allows for extensive surface modification and conjugation with biological molecules. In the provided context, the nanoparticles are conjugated with a targeting aptamer and a therapeutic drug, forming the QD-Apt(Dox) conjugate.

(b) Multifunctionality for Imaging and Therapeutics:

The QD-Apt(Dox) conjugate serves as an example of how particles can be used not only for imaging but also for targeted therapy. In this system, the quantum dot (a nanoparticle) is a carrier of A10 and Dox and serves as the second optical sensor. The optical signal is generated through a fluorescence resonance energy transfer (FRET) mechanism between the QD and the drug Dox, where the fluorescence is “OFF” when the drug is conjugated and restored (“ON”) upon its release inside target cells.

The ease of functionalization is highlighted: for biological applications, QDs are generally encapsulated with biocompatible polymers, functionalized for various bioconjugations.

(c) Applications in Molecular Imaging and Targeted Drug Delivery:

Nanoparticles in this size range can be used as vehicles for drug delivery. In the example given, a QD is combined with an A10 RNA aptamer that selectively targets the prostate-specific membrane antigen (PSMA), a marker overexpressed in prostate cancer cells. The successful conjugation and subsequent drug release of doxorubicin (Dox) provides a real-world demonstration of the theranostic (therapeutic + diagnostic) potential of nanoparticles engineered in such size ranges.

The controlled release and signal restoration allow real-time tracking of drug delivery, an important feature in developing precision medicine approaches.

Nanoparticles in the 5–15 nm range by the small GSH-Capped CuInS2 quantum dots have been developed by Zhao et al., [88]. In this work, the researchers achieved aqueous phase transfer of initial hydrophobic quantum dots using a ligand exchange strategy employing glutathione (GSH) and mercaptopropionic acid (MPA). The resulting quantum dots are described as having a small size (hydrodynamic diameter < 10 nm). The synthesis process involves an efficient ligand exchange that converts hydrophobic CuInS_2_/ZnS quantum dots into their aqueous-soluble form. This is achieved through a rapid procedure (the whole process takes less than 20 min) that is also scalable to gram amounts.

The GSH-capped quantum dots exhibit moderate fluorescent properties (up to 34%). This fluorescence makes them suitable for bioimaging applications, as they can serve as reliable fluorescence markers. Their strong and stable fluorescence is critical not only for imaging cells but also for potential applications in photocatalysis and solar energy conversion. Beyond their inherent fluorescence, the quantum dots have demonstrated the ability to penetrate cell membrane and image the cells, highlighting their potential as bioimaging markers.

One study by Rodzik et al. [89] describes the synthesis and complete characterization of fluorescent CdTe quantum dots conjugated with thymine. The studied particles were measured at 4–6 nm in size, the work provides insights into many properties relevant to nanoparticles in that general nanoscale regime of 5–15 nm. One key property reported was the optical behavior: a plasmon resonance fluorescence band at 540 nm on excitation at 351 nm was observed. Furthermore, the study showed that the intensity of this fluorescence band increased with the increasing amount of conjugated thymine without a shift in its position. This indicates that the optical properties of such nanoparticles are highly tunable by modifying their surface chemistry—a feature that is often exploited in nanoparticles within the 5–15 nm range. Another important finding was the selective interaction of the CdTe-thymine conjugates. The conjugates interacted efficiently and selectively with adenine as well as with adenine-containing modified nucleosides such as 5′-deoxy-5′-(methylthio)adenosine and 2′-O-methyladenosine. This selectivity underscores the potential of these nanoparticles to serve as part of biosensors, for example, in cancer diagnosis through monitoring urinary tumor markers.

In the work by Egloff et al. (2022) [90], the small size of the dye-loaded polymeric nanoparticles is noted as essential for their access to the intracellular mRNA targets in fixed permeabilized cells. in the general size range below 20 nm—are key to the technique described. This implies that nanoparticles in the 5–15 nm range would likely share the same advantage of being small enough to penetrate cellular compartments and enable effective in situ hybridization.

The study further details that these nanoparticles are small and ultrabright, which is achieved by encapsulating cyanine and rhodamine dyes with bulky counterions. As a result, the nanoparticles emit in green, red, and far-red channels and are 2–100-fold brighter than corresponding quantum dots. Their brightness and small size together allow for a simplified method—referred to as AmpliFISH—that can stain cells in only about 1 h, a significant reduction in time compared to conventional FISH techniques. Moreover, the optimized design minimized nonspecific intracellular interactions by an appropriate choice of the polymer matrix.

The nanoparticles are functionalized with DNA strands so that they provide sequence-specific imaging of different mRNA targets (for example, survivin, actin, and polyA tails). This design enables multiplexed detection, meaning that multiple RNA species can be simultaneously visualized in single cells. The high brightness and small size of the nanoparticles contribute to a signal that, based on image analysis, is essentially of single-particle nature, suggesting near single-molecule sensitivity. Their performance was also shown to be semiquantitative, with good correlation to RT-qPCR measurements. In comparison, the technique provides an 8–200-fold stronger signal relative to commercial locked nucleic acid (LNA)-based FISH, while involving far fewer protocol steps.

In summary, while the article does not specifically focus on a 5–15 nm particle size range, it clearly demonstrates that nanoparticles in the ultrasmall size regime (which would include particles sized 5–15 nm) are highly effective for intracellular applications. Their small size facilitates cellular access and interaction with mRNA targets, while their optimized brightness and functionalization allow for rapid, multiplexed, and sensitive RNA imaging essential for both cellular research and clinical diagnostics.

One relevant example of nanoparticles in the 5–15 nm range is the Ag_2_S quantum dots (QDs) described in the study by Zhang et al. (2018) [91]. These nanoparticles are water-soluble and carboxylic acid group-coated Ag_2_S QDs with an ultrasmall size (~8 nm). The nanoparticles exhibit a SPR wavelength of ~800 nm. This wavelength is especially favorable for photoacoustic imaging (PAI), a modality that benefits from contrast agents absorbing in the near-infrared region. Nanoparticles such as these Ag_2_S QDs offer a compelling combination of small size, favorable optical properties, targeted modification, and biocompatibility. These attributes make them promising candidates for use in advanced biomedical imaging techniques like photoacoustic imaging.

Ahmad et al. (2020) [92] prepared and characterized ZnO-engineered nanostructures, including quantum dots (QDs), that were 6 nm in size. Although their study investigated a range of sizes (6–100 nm), the 6 nm QDs clearly fall within the 5–15 nm range. The following points summarize their characteristics and effects:

When exposed to human HepG2 cells, at a concentration of 25–200 μg/mL, NPs induced dose-dependent cytotoxicity. Although this dose dependence was noted for the various sizes studied, it implies that even the smallest nanostructures in the 5–15 nm range (i.e., the 6 nm QDs) could contribute to cellular toxicity when used at similar concentrations. The study showed that the engineered NPs increased oxidative stress in a dose- and size-dependent manner, as evidenced by an increase in ROS production, lipid peroxidation, and glutathione reduction. This indicates that small nanoparticles in the 5–15 nm range are capable of initiating oxidative stress mechanisms that may lead to cellular damage.

When HepG2 cells were analyzed after a 24 h exposure, cell-cycle analysis revealed an increase in the apoptotic peak. At the molecular level, quantitative real-time PCR data showed that the mRNA levels of apoptotic marker genes such as p53, bax, and caspase-3 were upregulated, whereas bcl-2, an anti-apoptotic gene, was downregulated.

This pattern suggests that apoptosis mediated by nanoparticles—potentially including those in the 5–15 nm range—is executed through pathways involving p53, bax, caspase-3, and bcl-2.

Illustrated in the article Photoinduced Antibacterial Activity and Cytotoxicity of CdS Stabilized on Mesoporous Aluminosilicates and Silicates (Pharmaceutics, 2022) [93], nanoparticles within the 5–15 nm range were engineered to have potent photocatalytic and antibacterial activities while their cytotoxicity were modulated by the choice of carrier material. The study clearly demonstrates the potential of using such engineered nanoparticles to inactivate antibiotic-resistant bacteria under visible light irradiation while maintaining a favorable safety profile for eukaryotic cells.

#### 2.4.1. Graphene Quantum Dots (GQDs)

Graphene quantum dots are zero-dimensional derivatives of graphene, typically less than 10 nm in diameter, composed of single to few layers of graphene with quantum confinement and edge effects that impart size-dependent optical and electronic properties. Unlike traditional semiconductor QDs (e.g., CdSe, PbS), GQDs are metal-free, biocompatible, and exhibit high aqueous solubility, making them attractive candidates for clinical translation [94].

Key properties of GQDs:

- Strong and tunable photoluminescence (PL);
- High photostability and low photobleaching;
- Intrinsic biocompatibility and low toxicity;
- Surface functional groups (–OH, –COOH, –NH_2_) for conjugation.

Applications for cancer therapy include:

- Fluorescence imaging: GQDs offer bright and stable emission for cellular and in vivo tumor imaging, often in the near-infrared (NIR) range for deep tissue penetration. Challenges (GQDs)—Although metal-free and generally biocompatible, GQDs can still generate ROS under photoexcitation; batch-to-batch control surface groups and size dispersion < 10 nm should be verified with orthogonal methods to ensure consistent brightness and safety.
- Drug delivery: GQDs can be loaded or conjugated with chemotherapeutic agents (e.g., doxorubicin, paclitaxel) via π-π stacking and hydrogen bonding.
- Photodynamic and photothermal therapy (PDT/PTT): GQDs generate reactive oxygen species (ROS) upon light excitation, inducing apoptosis in tumor cells. Their photothermal conversion efficiency also allows heat-induced cancer cell ablation [95]. Challenges (QGDs—PDT/PTT)—Phototoxicity windows, heat generation thresholds, and tumor-to-normal selectivity require careful dose and light-dose control; CQAs should include size distribution and surface chemistry that modulate ROS and heat generation.

Recent studies have also explored GQD-based nanohybrids, such as GQDs embedded in liposomes or polymeric matrices, to combine imaging and therapy (theranostics). Additionally, the low toxicity of GQDs compared to heavy-metal QDs gives them an edge in biomedicine [96].

The following are some examples of Graphene quantum dots (GQDs):

In the research, the GQDs were enhanced for imaging purposes by radiolabeling them with Technetium-99m (99mTc) using SnCl_2_·2H_2_O as a reducing agent. This preparation resulted in a high radiochemical yield of >97% (Mazaheri Tehrani et al., 2025) [97], underscoring the efficiency with which nanoparticles of this size can be engineered for specific biomedical roles. The study also demonstrated that once the nanoparticles were radiolabeled, they showed promising biodistribution characteristics. Not only were they taken up by the tumor, but they were also distributed in other organs, such as the kidneys and intestines, as observed via scintigraphy imaging. This is important for evaluating both the targeting specificity and the potential off-target effects when using nanoparticles in clinical settings. While these nanoparticles exhibit many useful characteristics, stability remains a critical factor. In the study, it was noted that almost 15% of 99mTc-labeled GQDs degraded after 6 h when incubated in human serum. Although this is a moderate degradation rate, it emphasizes the ongoing need for optimizing nanoparticle formulations to enhance stability without compromising their imaging capabilities.

In the work by Wang et al., the authors state that the detached GQDs can take advantage of their extremely small size (5–10 nm) to penetrate deeply into tumor tissues (Wang et al., 2024) [98]. The extremely small dimensions of these GQDs (5–10 nm) allow them to overcome one of the limitations of traditional nanoparticles, namely low tumor permeability. Their small size enables them to penetrate deeply into tumor tissues, which is a valuable property for targeted drug delivery. In the reported study, the GQDs are part of a larger drug delivery system. They are gated on the mesopores via disulfide bonds of a mesoporous carbon nanoparticle (MCN) framework that itself contributes to photothermal conversion. This design allows dual functionality—drug delivery and photothermal therapy—by integrating the benefits of both the MCN and the GQDs.

The system is designed so that in the presence of a high glutathione concentration, the disulfide bonds are ruptured under high glutathione concentration in the tumor microenvironment. This responsive mechanism triggers the release of the drug doxorubicin (DOX) and facilitates the detachment of GQDs. When they detach, their small size is exploited to enhance the accumulation and deep penetration of therapeutic agents within tumor tissues.

In the study by Vahedi et al. (2022) [99], nanoparticles in the range of 5–15 nm were exemplified by the graphene quantum dots (GQD) and their modified form with hyaluronic acid. The study reports that TEM images revealed particle sizes of ~5.67 and ~8.69 nm, respectively, for the GQD and the hyaluronic acid-conjugated GQD, placing them well within this size range (Vahedi et al., 2022) [99].

The small size of these nanoparticles has several implications:

A model drug (curcumin) was loaded onto the nanocarrier with a high loading percentage of 98.02%. This high efficiency is partly attributed to the large surface-area-to-volume ratio that nanoparticles in this size range offer, which is ideal for maximizing drug payload. Besides drug delivery, nanoparticles serve a dual purpose by acting as imaging agents. The inherent photoluminescence of the graphene quantum dots allows for fluorescent imaging of cancer cells. The study confirms that fluorescent microscopy showed that these nanocarriers were adsorbed on HeLa cells, unlike L929 cells, indicating selective accumulation in target cells. The nanocarrier exhibited complete biocompatibility in vitro assays. The viability studies on HeLa and L929 cells, assessed via the MTT assay, showed that while the nanocomposite is safe for normal cells, it significantly reduces the viability of cancer cells due to its targeted delivery mechanism.

#### 2.4.2. Carbon Nanotubes (CNTs)

CNTs are carbon-based well-ordered cylindrical molecules that consist of a single layer of carbon atoms (grapheme sheet) rolled into a cylinder. The configuration of CNTs includes multi-walled or single-walled configurations, or nanotubes interlinked concentrically that are composed of C_60_ fullerenes [100]. Carbon nanotubes have high loading capacities as non-polymeric drug carriers, imaging contrast agents, and biological sensors [101,102]. Fullerenes, due to their increased number of conjugated double bonds, change in the graphite cylinder arrangement, and are most often used in the delivery of therapeutic agents such as anticancer agents, antibiotics, and antiviral agents [103,104]. Single-walled carbon nanotubes (SWCNTs) have specific characteristics, such as a diameter ranging from 0.7 to 3 nm and thin flexible shapes, whereas multi-walled carbon nanotubes consist of several concentric SWCNT layers with diameters ranging from 1.5 nm to 220 nm [105]. Functionalization allows the CNTs to conjugate a variety of biomolecules such as carbohydrates, proteins, peptides, and therapeutic and diagnostic devices to generate economic, vastly functional therapeutic and diagnostic strategies which are also accurate [106]. CNTs with hollow tube-like structures hold molecules via capillary action and adsorption and have a crucial role in drug delivery applications [107].

Distinctive features of CNTs (<15 nm in diameter):

- High mechanical strength and thermal conductivity;
- Exceptional near-infrared (NIR) absorbance;
- Large surface area for drug or gene attachment;
- Intrinsic fluorescence and Raman scattering for imaging.

Applications for cancer include:

- Drug and gene delivery: CNTs are excellent vehicles for loading chemotherapeutic agents (e.g., cisplatin, doxorubicin), siRNA, or DNA through covalent or non-covalent interactions. Despite strong loading and NIR absorption, sub-15 nm CNTs face biopersistence-linked inflammation risks and typically require robust surface functionalization to improve dispersibility and biocompatibility; these constraints should be considered alongside the efficacy data. Functionalization with PEG, folic acid, or peptides improves dispersibility and targeting [108].
- Photothermal therapy (PTT): SWCNTs efficiently convert NIR light into heat, achieving localized tumor ablation.

Imaging and biosensing: CNTs serve as contrast agents in photoacoustic imaging and fluorescence-guided surgery [109]. In this section, we further discuss the significance of CNTs in diseases.

A notable example of nanoparticles in the 5–15 nm range is represented by the AgNPs used to decorate multi-walled carbon nanotubes (MWCNTs) in the study. The authors report that these AgNPs attained a diameter of 11 nm ± 2 nm (Gamiño-Barocio et al., 2024) [110]. The functionalization of MWCNTs with these AgNPs played a significant role in promoting reactive species formation in an aqueous medium, especially when assisted by UV irradiation. UV-Vis spectroscopy in the study demonstrated that the reactive species density increased by 4.07 times after the MWCNTs were functionalized with the nanoparticles. These properties allow them to be effectively used in applications where reactive species generation is beneficial, such as in the context of cancer cell interactions, as indicated by their operational mechanism in an aqueous medium under UV light.

Despite their promising features, CNTs face challenges in biomedical applications due to concerns about:

- Biopersistence and slow clearance;
- Inflammatory responses and cytotoxicity;
- Need for extensive surface functionalization to enhance solubility and biocompatibility.

Recent advancements in ultra-short CNTs (USCNTs) and oxidized CNTs have addressed many of these issues, making sub-20 nm CNTs safer and more effective for clinical translation [111].

#### 2.4.3. Comparative Insight and Outlook Between GQDs and CNTs

The comparative insight and outlook between GQDs and CNTs are summarized in Table 5 below.

GQDs (<10 nm) are metal-free, bright, and readily functionalized, making them strong for fluorescence imaging, PDT, and light-guided chemo with lower persistence risk; drug loading is moderate (π-π/H-bonding). CNTs (diameter often < 15 nm) offer exceptional loading (interior/exterior), NIR photothermal potency, and biosensing/PA imaging, but require oxidation/PEGylation to mitigate biopersistence and inflammation. GQDs are useful when biocompatibility and imaging are primary requirements. CNTs are used when high payload density and PTT are required.

The combination of small size, tunable surface chemistry, and multifunctionality makes sub-15 nm QDs and carbon-based nanostructures ideal candidates for next-generation cancer theranostics. Their integration with other nanomaterials (e.g., metals, polymers, lipids) and surface ligands offers exciting opportunities to develop smart, responsive, and personalized nanomedicines for cancer therapy.

#### 2.4.4. Synthesis of Sub-15 nm Nanoparticles

Graphene quantum dots (GQDs) are synthesized by top-down oxidative cutting of graphene/GO (acid oxidation, hydro/solvothermal) process or bottom-up carbonization (e.g., citric-acid/biomass precursors) and subsequently passivation/doping for PL tuning. To maintain the particle size ≤ 15 nm, reaction time, temperature, oxidant strength, and precursor dilution are controlled. Finally, arrowed down the particle sizes with post-fractionation. The production constraints are batch heterogeneity in sizes and PL loss on aqueous ligand exchange. There is a need for benign corona or stealth for stability. The particles are very useful for NIR imaging, PDT/PTT, and theranostics [112,113,114,115,116].

Carbon nanotubes (CNTs) are produced via CVD growth (SWCNT/MWCNT) and then cutting or oxidation to ultrashort CNTs. The CNTs are then dispersed via surfactants or covalent PEG or ligands. The diameter ≤ 15 nm or ultrashort length is achieved by oxidative shortening, rigorous debundling, narrow dispersity by centrifugation, and SEC. The constraints of this production process include the removal of metal catalysts, biopersistence, and toxicity. If poorly functionalized, there will be aggregation without robust coatings. The CNTs are best for PTT, high-loading delivery, photoacoustic, and fluorescence imaging [117,118,119].

Dendrimers of 5–12 nm size are synthesized by divergent iterative growth (e.g., PAMAM), intramolecular folding, optional crosslinking, and ligand/drug conjugation. To keep particle sizes less than ≤15 nm generation G3–G5, low-MW scaffolds are chosen. The major problems for this production process are cost/throughput of iterative steps; defect control; precise ligand-density quantification; and avoiding inter-particle crosslinking. The particles are useful for multivalent targeting, precision drug conjugates, dual-mode imaging [120].

Lipidic micelles (~5–15 nm) are self-assembly of amphiphiles (PEG-lipids, biomimetic surfactants) and produced by solvent exchange or microfluidic mixing. To keep ≤15 nm, low aggregation number (short blocks/peptides), optimized solvent switch, high graft-density stealth are used. The production constraints are dilution-induced destabilization and leakage of payload; payload–curvature mismatch; and narrow PDI control during scale-up. The particles are suitable for solubilizing hydrophobes, rapid penetration, and ligand-targeted delivery [121,122,123].

Nanolipoprotein particles (NLPs)/or HDL-mimetics (~9–12 nm) of discoidal bilayer stabilized by ApoA-I (or mimetic peptides) are produced via cholate-dialysis or microfluidic reconstitution. The membrane proteins can also be inserted. To maintain particle size ≤15 nm, peptide: lipid stoichiometry, lipid composition, and ionic strength are important. The production challenges are protein source and quality, batch reproducibility, and ligand display. These particles are suitable for membrane–protein display, imaging, and receptor-mediated uptake (LDLR/SRB1) [124].

Nanoemulsions (≤15 nm) are produced by high-energy (microfluidization/ultrasonication) or spontaneous emulsification (low oil, mixed surfactants). The particle sizes are kept lower than 15 nm by low dispersed-phase fraction, ensuring the right HLB blends, and using co-solvents, resulting in immediate quenching of droplet growth. The particles are kinetically (not thermodynamically) stabilized. Regarding Ostwald ripening, surfactant load vs. biocompatibility are the constraints for these particles. They are best for hydrophobic actives, combo-payloads, and transferrin/other ligand targeting [125,126].

Metallic nanoparticles (Au, Ag) (2–15 nm) are produced by thiolate (Brust-type) reductions for ~1–5 nm monolayer-protected clusters; citrate/borohydride reductions for ~5–15 nm; seed-mediated growth for shape control. The passivation is performed by PEG/zwitterion. The ≤15 nm particle sizes are achieved via the thiol/metal ratio, strong reducing conditions, rapid quenching, and by preventing post-growth ripening. The constraint for this process is salt/protein-induced aggregation; ligand exchange swelling; core–shell uniformity for Ag@Au. These particles are best for PTT, SERS/SPR sensing, radio sensitization, and hybrid theranostics [127,128].

Iron oxide (Fe_3_O_4_/γ-Fe_2_O_3_) (4–12 nm) nanoparticles are produced by thermal decomposition of Fe precursors (monodisperse cores) or co-precipitation (simpler, broader PDI); surface ligand exchange to hydrophilic shells. The particle sizes are kept ultrasmall by hot-injection time and ligand selection. The production constraints are oxidation, magnetization loss, aqueous transfer reduces stability and coating integrity for MRI/CDT. They are best use for MRI contrast, magnetically assisted therapy and targeting [129,130,131].

Semiconductor QDs (Ag_2_S, CuInS_2_/ZnS) (3–10 nm) are prepared by hot-injection or heat-up syntheses; optional ZnS shell; aqueous transfer via MUA/DHLA. Their sizes are kept ≤15 nm by maintaining short growth time and low temp; tight nucleation burst; minimal shell thickening. The main constraints for this production process are PL drop after ligand exchange; shell defects; keeping hydrodynamic size ≤ 15 nm with bioligands. They are best for bright and long-lived imaging, intraoperative guidance [88,132]. The comparison of different synthesis methods of less than 15 nm particles are shown in Table 6.

### 2.5. Magnetic Nanomaterials and Metal-Organic Frameworks (MOFs) for Quantum Technologies

Magnetic nanoparticles and MOFs are rapidly emerging as solid-state platforms for spin-based quantum technologies (qubits, quantum sensors, and spin-photon interfaces). In particular, porphyrinic 2D MOF nanosheets and vanadyl/lanthanide coordination architectures have shown promising spin-lattice relaxation and coherence characteristics compatible with coherent control, including integration with superconducting resonators and operation at elevated temperatures. These systems leverage molecular precision and nanoscale confinement to suppress environmental noise while preserving addressable spins—an approach that aligns naturally with the design philosophy of sub-15 nm nanostructures [133].

#### 2.5.1. Why Size Matters in Nanoscale Confinement

Quantum coherence in molecular and solid-state spins is limited by phonons, dipolar spin–spin interactions, and conformational motion. Tailoring size and morphology—shrinking particle/domain size, thinning to few-layer/nanosheet architectures, and using short-period, rigid linkers—can (i) reduce the phonon density of states that couples to spins, (ii) dilute spin ensembles to weaken dipolar broadening, and (iii) restrict chromophore or ligand motion, thereby extending coherence times (T_2_) and enabling entangled states. Recent porphyrinic MOFs illustrate these principles: vanadyl-porphyrin 2D frameworks and Cu(II) phthalocyanine nanosheets exhibit optimized spin-lattice relaxation and improved spin dynamics due to their ordered, low-dimensional architecture and controlled inter-spin spacing.

#### 2.5.2. Room-Temperature Entanglement in a MOF

Room-temperature quantum coherence of an entangled quintet multiexciton (^5^TT) generated by singlet fission in a chromophore-integrated MOF, with coherence exceeding 100 ns, is described in a 2024 study [134]. Most importantly, ordered domains within MOF nanocrystals suppress chromophore motion just enough to support ^5^TT generation while avoiding decoherence, directly linking nanoscale structural control to coherence and entanglement at ambient conditions—an important milestone for scalable quantum sensing/processing in biological environments. These results demonstrate how size and domain engineering in MOFs can boost coherence and enable entanglement at practical temperatures.

#### 2.5.3. Relevance to Sub-15 nm Bionanotechnology

MOF crystals and 2D sheets exceed 15 nm laterally, but their active quantum subunits (porphyrins, vanadyl centers, chromophores) and pore-confined domains are intrinsically sub-nanometer to few-nanometer building blocks. Utilizing sub-15 nm nanoclusters/ultra-thin domains or embedding these quantum-active motifs into sub-15 nm carriers (e.g., dendrimers, micelles, or lipoprotein-like nanoparticles) could enable deep-tissue access and renal-clearable pharmacokinetics while preserving quantum functionality for in vivo magnetometry, microenvironment sensing, and theranostics—synergizing with the pharmacological advantages emphasized throughout this review.

## 3. Unique Physicochemical Properties of Sub-15 nm Nanoparticles

The size of nanomedicine critically affects its accumulation, penetration, and efficacy in tumors [8,135,136,137,138,139]. Nanocarrier-based drugs with sizes below 15 nm have been demonstrated to possess much improved in vivo pharmacokinetics with superior penetration and retention behaviors, decreased reticuloendothelial system (RES) uptakes, and enhanced blood circulation [140,141,142].

### 3.1. Size-Dependent Surface Area and Reactivity

Nanoparticles under 15 nm in size exhibit distinct physicochemical behaviors compared to their larger counterparts, driven primarily by their ultrasmall dimensions, high surface-area-to-volume ratios, and quantum-scale phenomena. These properties significantly influence their interactions with biological systems and therapeutic performance in cancer applications. One of the most critical aspects is the size-dependent increase in surface area and surface reactivity, which governs drug loading, cellular interactions, catalytic behavior, and clearance kinetics.

#### 3.1.1. Surface-Area-to-Volume Ratio: A Key Driver of Nano-Bio Interactions

As particle size decreases, the proportion of atoms or molecules located on the surface increases dramatically. For example, a spherical nanoparticle of 10 nm diameter has approximately 10× more surface area per unit volume than a 100 nm particle. This increased surface area allows:

- Greater surface functionalization (e.g., with targeting ligands, PEG chains, drugs, fluorophores)

However, in the sub-15 nm regime, the reduced corona thickness can also present challenges. A thinner steric barrier means that grafted moieties (e.g., PEG chains, targeting ligands, fluorophores) may experience steric crowding and reduced conformational freedom, which in turn can impair their accessibility to target receptors. It is reported that functionalized ultrasmall gold nanoparticles (<15 nm) retain high binding efficiency when corona composition is optimized, confirming that this trade-off can be managed through rational design [143]. The paper by Andrian, T. et al. [144] quantitatively showed how dense or long PEG chains hinder ligand availability (steric masking), and how mixed/cocktail PEG architectures can mitigate that.

- Enhanced dispersion in aqueous media due to higher surface energy
- More rapid interactions with the cellular membrane and proteins

The surface-area-to-volume ratio (SA: V) is mathematically defined as:

SA: V = 1/(d6)

where d is the particle diameter (assuming a spherical shape). As d decreases to below 15 nm, SA: V increases very fast, which directly enhances reactivity and bioavailability. This is especially beneficial for applications such as:

- Drug delivery (higher loading per unit mass of carrier)
- Catalytic cancer therapy (e.g., Fenton-like reactions in chemodynamic therapy)
- Diagnostic signal amplification (e.g., in fluorescence or photoacoustic imaging)

#### 3.1.2. Surface Reactivity and Chemical Functionality

In sub-15 nm nanoparticles, the high curvature and unsaturated surface atoms contribute to unique surface chemistry:

- Higher chemical reactivity: The atoms on the surface are less coordinated than those in the bulk, leading to more available sites for reactions or interactions with biomolecules.
- Enhanced redox activity: For metal or metal oxide nanoparticles, this translates to increased ROS generation, which is useful in therapies like photodynamic or chemodynamic treatment of tumors.
- Increased adsorption capacity: Smaller nanoparticles can adsorb a higher quantity of drugs or proteins due to their large surface area and increased surface free energy.

For example:

- Gold nanoparticles (AuNPs) < 10 nm show dramatically higher binding affinities for thiolated ligands than larger AuNPs, enabling denser and more stable functional coatings [145].
- Graphene quantum dots (GQDs) < 5 nm possess abundant edge sites and oxygen-containing functional groups, offering superior reactivity for drug conjugation and ROS generation under light irradiation [95].

We further discuss the significance of surface area for particles with size lower than 15 nm in different applications.

Hamida et al. (2023) [146] provide an excellent example of biofabricated silver nanoparticles (AgNPs) that fall within the 5–15 nm range. This feature of high surface-to-volume ratio is associated with enhanced chemical reactivity and improved interaction with biological targets. In the study, the small size and stability translated into potent biological activities: the Nos@AgNPs exhibited significant inhibitory activity against lung, colon, and breast cancer cells as well as considerable biocidal activity against Staphylococcus aureus, Escherichia coli, Klebsiella pneumonia, and Pseudomonas aeruginosa (Hamida et al., 2023) [146]. Significant anticancer and antimicrobial activities, which are partly attributed to their small size and high surface reactivity.

In the study by Sumathi et al. (2023) [147], zirconia nanoflakes (ZrNFs) with sizes in the 8–15 nm range were synthesized using a green, bottom-up approach. The study also examined electrophysiological behavior using cyclic voltammetry (CV). It was observed that the slower rate of electron transfer was correlated with the interaction of the ZrNFs with biological systems, suggesting potential for controlled redox reactions in situ. This controlled electron transfer is a key characteristic of metal oxide nanoparticles in this size range that could be beneficial for biomedical and biosensing applications. In summary, the work by Sumathi et al. (2023) [147] illustrates that nanoparticles in the nanoscale range—specifically the zirconia nanoflakes of 8–15 nm—demonstrate significant potential in biomedical applications owing to their unique physicochemical properties, including enhanced surface reactivity, unique optical and electrical behaviors, and effective interaction with biological cells.

AuNPs in the 5–15 nm range, as exemplified by the 10 nm particles in the study by Abdelhalim [148], have distinctive properties including a spherical morphology, enhanced surface reactivity due to a high surface-to-volume ratio, and significant effects on fluorescence signals used to track organ-specific uptake. Their reactivity and biological interaction profiles underline both the potential utility in medical applications and the need to carefully consider toxicity and clearance profiles.

Nanoparticles in the 5–15 nm range, exemplified by the CuO-C nanoparticles with a crystallite size of approximately 14.65 nm and a grain size of 14.09 nm, exhibit distinct beneficial properties. Their small dimension contributes to a high surface reactivity and facilitates biological interactions, making them promising candidates for a variety of biomedical applications such as antioxidant, antimicrobial, and anticancer treatments (Kimta et al., 2025) [149].

The study by Huang RH, et al. (2020) [150] focuses on the surface modification of barium titanate nanoparticles to enhance their stability and dispersibility in aqueous environments. The authors explore how these modifications influence nanoparticles’ surface activity, which is critical for applications in various fields, including drug delivery and biocompatibility. The findings demonstrate that tailored surface properties can significantly affect the performance of nanoparticles in biological settings.

#### 3.1.3. Quantum Effects and Size-Dependent Optical Properties

Below 10–20 nm, many nanoparticles begin to exhibit quantum confinement effects, especially semiconductor quantum dots and carbon-based nanostructures. This affects:

- Optical absorbance and fluorescence: Smaller particles exhibit blue-shifted emission and tunable photoluminescence, enabling size-controlled imaging probes.
- Photothermal and photodynamic conversion efficiency: Higher surface reactivity improves energy transfer for cancer cell ablation.

These effects are size-dependent and enable the tailoring of nanoparticle properties to specific therapeutic or diagnostic goals.

The following are some examples of quantum effects and size-dependent optical properties of nanoparticles in the size range 5–15 nm.

The study by Hassanien et al. [151] reports that the biosynthesized copper nanoparticles (Cu-NPLs) have diameters ranging from 4.7 to 17.4 nm. The nanoparticles exhibit a characteristic SPR as demonstrated by UV–vis spectroscopy—it shows an SPR peak at 563 nm. This optical feature is related to the particles’ ability to interact with light, and the measured energy bandgap was 2.1 eV. In addition, the electrical conductivity of these Cu-NPLs was determined to be 1.04 × 10^−6^ S·cm^−1^ at 120 K, suggesting potential utility in electronic devices.

AgNPs in the range of 5–15 nm were synthesized from Bacillus sp. KFU36 by Almalki et al. in 2020 [152]; they were comprehensively characterized and evaluated for their biological properties. Optically, the AgNPs showed a band of SPR at 430 nm as revealed by UV–vis spectrophotometry, which is indicative of their size-dependent optical characteristics. In addition, structural characterization through X-ray diffraction confirmed the nanoparticles’ crystalline structure, and Energy Dispersive Spectroscopy substantiated their metallic nature.

The article by Wan et al., 2019 [153] highlights that nanomaterials with localized surface plasmon resonance (LSPR) have exquisite optical properties, a feature that is particularly pronounced in non-stoichiometric copper sulfides. These materials exhibit active LSPR with a peak in the near-infrared (NIR) region, making them suitable for deep bioimaging applications and photothermal therapy (PTT).

#### 3.1.4. Implications for Drug Delivery and Cancer Therapy [59]

The increased surface area and reactivity of sub-15 nm nanoparticles provide several therapeutic advantages:

- Efficient cellular uptake: Their small size and reactive surfaces allow better interaction with cell membranes, promoting endocytosis.
- Improved tumor penetration: Smaller nanoparticles navigate through the dense extracellular matrix more effectively than larger systems, achieving more uniform drug distribution within tumors.
- Targeted delivery: The abundance of surface sites allows for multivalent conjugation of targeting moieties, enhancing specificity to tumor cells or receptors (e.g., folate, RGD peptides).
- Stimuli-responsiveness: Reactive surfaces can be engineered to respond to tumor-specific cues such as pH, redox, or enzymatic activity.

However, high surface reactivity can also pose challenges, including:

- Potential toxicity or oxidative stress
- Instability due to agglomeration
- Non-specific protein adsorption (opsonization)

Thus, surface passivation or functionalization (e.g., PEGylation, zwitterionic coatings) is essential for achieving optimal therapeutic outcomes.

In summary, the increased surface area and reactivity of nanoparticles under 15 nm are fundamental to their enhanced performance in cancer nanomedicine. These features underpin their ability to load more therapeutic agents, interact dynamically with tumor cells, and respond to microenvironmental stimuli. Harnessing and controlling these properties are crucial in designing safe, effective, and intelligent nanotherapeutics for clinical translation.

### 3.2. Enhanced Cellular Uptake and Biodistribution

The in vivo performance of nanoparticles in cancer therapy is closely tied to their ability to reach the tumor site, distribute within tissues, and enter cells efficiently. Nanoparticles with sizes less than 20 nm exhibit unique biological behaviors compared to larger counterparts, particularly in terms of cellular internalization pathways, tumor penetration, and systemic biodistribution. These differences stem from their small size, high surface curvature, and increased diffusivity, which collectively influence how these particles interact with biological barriers.

For example, in the study on different particle sizes of DOX-loaded mixed micelles for cancer therapy by Li et al. (2020) [154], doxorubicin (DOX) was loaded into lipid/glycocholic acid mixed micelles (LGs) that were prepared in two distinct sizes, one of which was around 10 nm. Although the study compared 10 nm particles to larger ones (~100 nm), the results for the 10 nm particles provide insight into the benefits of nanoparticles within the 5–15 nm range. The DOX-loaded LGs at 10 nm exhibited higher cellular uptake capacity compared with their 100 nm counterparts. This suggests that nanoparticles in the 5–15 nm range may more efficiently enter cancer cells, which is crucial for delivering chemotherapeutic agents effectively. In vivo near-infrared fluorescence (NIFR) imaging demonstrated that DOX-LGs at 10 nm had more accumulation in the tumor site. Enhanced tumor accumulation is a critical factor in cancer therapy, as it increases the local concentration of the drug at the target site while potentially reducing systemic side effects.

Glutathione (GSH)-coated, folic acid (FA)-modified spherical AuNPs with a diameter of 5.6 nm were synthesized by Yücel et al. in 2020 [155]. These nanoparticles were further loaded with methotrexate (MTX) to yield a drug complex (MTX/Au-GSH-FA) of about 11 nm. According to the study, DLS and TEM results showed that MTX/AuNPs possess spherical morphology, nanoscale particle size, narrow size distribution, and good stability. The size range of 5–15 nm is especially important because it allows the nanoparticles to navigate the biological milieu effectively. Their small size enables enhanced permeability and retention, characteristics that are crucial for reaching target cells, and they offer a high surface-to-volume ratio that facilitates the conjugation of multiple functional molecules—such as drugs and targeting ligands.

A nanoparticle formulation in the 5–15 nm range is a lipid-based nanomicellar system developed for vinorelbine. In this formulation, phospholipid nanomicelles with a mean size of approximately 15 nm were used (Bahadori et al., 2014) [156]. The nanomicellar formulation was shown to be ~6.7-fold more potent than vinorelbine dissolved in DMSO when tested on the MCF-7 cell line. This enhanced potency is likely due to the improved delivery and biodistribution provided by the nanoscale carrier.

Nanoparticles in this size range demonstrate favorable biodistribution and cellular uptake properties. The document by Dziawer et al., 2019 [157] reports that in vitro biological studies indicated that 211At-AuNP-PEG-trastuzumab exhibited higher affinity and cytotoxicity towards the HER2-overexpressing human ovarian SKOV-3 cell line than unmodified nanoparticles. Moreover, confocal and dark field microscopy studies revealed that 211At-AuNP-PEG-trastuzumab was effectively internalized and deposited near the nucleus, suggesting that the small size facilitates penetration and accumulation in key subcellular locations.

#### 3.2.1. Size as a Determinant of Cellular Uptake Pathways

Cellular uptake of nanoparticles occurs primarily via endocytosis, including clathrin-mediated, caveolae-mediated, macropinocytosis, and phagocytosis. The mechanism and efficiency of uptake are highly size-dependent. Nanoparticles under 15 nm have been shown to:

- Enter cells more rapidly than larger particles
- Preferentially undergo caveolae- or clathrin-mediated endocytosis, which allows escape from lysosomal degradation
- Access subcellular compartments such as the nucleus, mitochondria, or endoplasmic reticulum more readily [158]

A study by Chithrani et al. (2006) demonstrated that 14–20 nm AuNPs exhibited maximum uptake efficiency in HeLa cells compared to both smaller and larger particles, highlighting the importance of optimizing particle size for internalization [159].

As illustrated by the work of Silva et al. (2018) [160], nanoparticles in the 5–15 nm range combine favorable size-dependent properties such as superparamagnetism, enhanced cellular uptake, and colloidal stability, making them ideal for advanced biomedical applications like targeted cancer therapy. The detailed synthesis and characterization described in the study serve as a model for designing nanoparticles that not only operate effectively at the nanoscale but also exhibit modified surfaces to improve stability and functionality in biological environments.

#### 3.2.2. Tumor Penetration and Interstitial Diffusion

**Figure 1:**
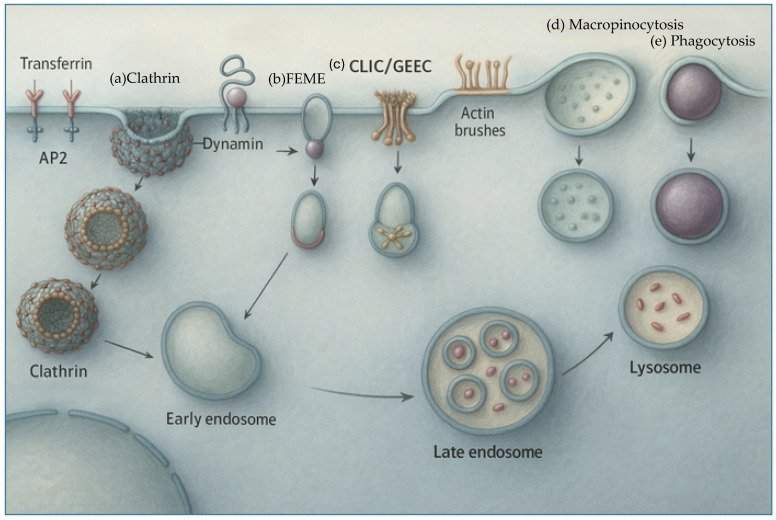
Endocytic pathways: (a) clathrin-mediated endocytosis (CME), (b) fast endophilin-mediated endocytosis (FEME), (c) clathrin-independent carrier endocytosis (CLIC/GEEC), (d) macropinocytosis, and (e) phagocytosis. Adapted from [161]. Copyright@ 2021, Springer Nature.

A major challenge in solid tumor therapy is achieving deep and uniform distribution of therapeutic agents throughout the tumor mass. Larger nanoparticles (>50 nm) often accumulate near leaky tumor vasculature (perivascular localization) but fail to penetrate deeper due to high interstitial fluid pressure, dense extracellular matrix (ECM), and tight cell–cell junctions (Figure 1).

Sub-15 nm nanoparticles demonstrate:

- Superior interstitial diffusion in 3D tumor spheroids and dense tumor stroma
- Ability to overcome size-exclusion effects posed by collagen networks and ECM pores (~20–50 nm)
- Enhanced paracellular transport and transcytosis across endothelial barriers [136]

These properties result in more homogeneous intratumoral distribution, increasing therapeutic efficacy and reducing the risk of drug resistance caused by poorly reached tumor cells.

**Figure 2:**
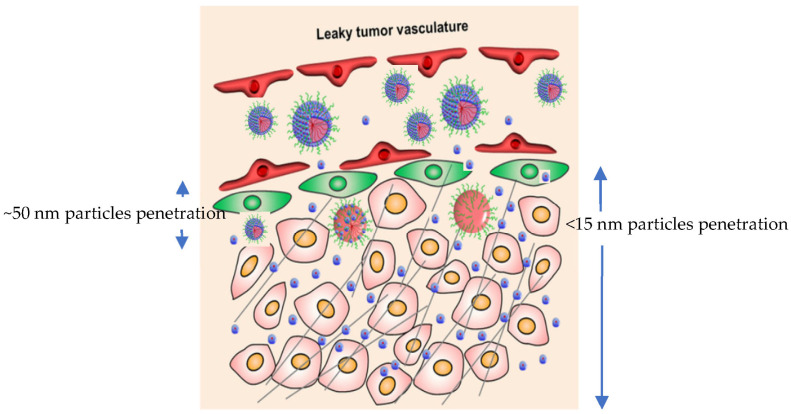
Sub-15 nm carriers achieve more homogeneous intratumoral distribution by crossing endothelial barriers (paracellular/transcytosis) and diffusing through ECM meshes whose effective pores are ~20–50 nm. In contrast, ≥50 nm systems pool perivascularly. Evidence summarized in Section 3.2.2, including size-dependent penetration data for ultrasmall dendrimers and multistage systems that release ~10 nm sub-particles to improve interstitial transport.

Superior tumor penetration of sub-15 nm nanoparticles is shown diagrammatically in the following Figure 2.

One of the key observations by Bungo et al., 2019 [162] was that nanoparticle size plays a crucial role in tumor penetration. The authors compared PAMAM dendrimers of different generations and sizes and reported that smaller generation 2 dendrimers (G2-NH_2_, 2.9 nm diameter) penetrated 2.5-fold deeper than larger G7-NH_2_ (8.1 nm). Generally, as particle size increases, the ability to penetrate deeply into tumor tissue diminishes. Thus, nanoparticles on the lower end of the 5–15 nm range may be expected to exhibit better tissue penetration compared to those closer to or above 10 nm. In addition to size, the study examined the role of nanoparticle rigidity, finding that “smaller rigid AuNP (2 nm) penetrated significantly more than larger AuNP (4 nm) (3-fold, *p* = 0.014; G2-NH_2_ vs. G4-NH_2_, 2.8-fold, *p* = 0.033). This finding implies that while rigidity does have a measurable effect, it may be less critical than particle size when designing nanoparticles for enhanced tumor penetration. For nanoparticles in the 5–15 nm range, ensuring an optimal balance between size and rigidity could be key to maximizing delivery efficiency.

As described in the multistage nanoparticle delivery system, a larger 100 nm particle shrinks to release smaller nanoparticles that are around 10 nm in diameter once they encounter specific proteases in the tumor microenvironment (Wong et al., 2011) [163]. The smaller 10 nm nanoparticles are able to more readily diffuse throughout the tumor’s interstitial space because their reduced dimensions ease movement through the dense collagen matrix that can otherwise hinder larger nanoparticles. Although the study specifically mentions 10 nm particles, this falls within the broader 5–15 nm range of interest.

In summary, nanoparticles in the 5–15 nm size range (exemplified by the 10 nm quantum dots described) offer a strategic advantage in tumor-targeted therapy. Their small size facilitates deeper penetration into the tumor interstitial space, overcoming the physiological barriers present in the tumor microenvironment. This improved penetration is achieved via a protease-triggered mechanism that converts a circulating larger nanoparticle into an effective, smaller therapeutic agent.

#### 3.2.3. Biodistribution and Pharmacokinetics

The pharmacokinetics of sub-15 nm nanoparticles differ significantly from larger nanoparticles:

- Circulation time: Depending on surface properties (e.g., PEGylation), sub-15 nm particles may circulate long enough to exploit the enhanced permeability and retention (EPR) effect, though ultrasmall particles (<5 nm) may undergo rapid renal clearance.
- Renal clearance: Nanoparticles below ~6 nm are rapidly filtered by the kidneys and excreted in urine, which may reduce systemic toxicity and off-target accumulation.
- RES evasion: Sub-15 nm particles can partially evade RES capture by combining hydration-layer stealth (PEG, polysarcosine, poly(2-oxazoline), zwitterions) with corona-quality control (dysopsonin-favoring interfaces), which together extend circulation and improve tumor exposure.
- Liver and spleen accumulation: Although liver uptake is common for many nanoparticles, the reduced opsonization of smaller particles improves their biodistribution to peripheral and tumor tissues.

Thus, engineering particle size to remain just above the renal threshold (~6–8 nm) yet small enough to diffuse effectively (~10–20 nm) offers an optimal balance between tumor penetration and systemic retention.

The superparamagnetic iron oxide nanoparticles (SPIONs) studied are engineered for medical applications such as diagnostic imaging and targeted cancer therapy. In this study, two types of SPIONs were investigated in anesthetized pigs: one was a DMSA-coated nanoparticle labeled MF66 with a core nominal diameter of 12 nm, and the other was called OD15 with a 15 nm overall diameter (Edge et al., 2016) [164].

Pharmacokinetics: When administered intravenously into pigs, the OD15 (at a dose of 2.0 mg/kg) was shown to have a plasma half-life of approximately 15 min. This relatively short half-life suggests rapid clearance from the bloodstream, which can be an important consideration in balancing imaging timing with therapeutic window and potential toxicity.

Biodistribution: Magnetic resonance imaging (MRI) at 1.5 T and subsequent histology confirmed that, regardless of the initial detection challenges with the MF66 nanoparticles (which were below detection limits in blood samples), both nanoparticle types accumulated mainly in the liver and spleen. MRI also revealed that both particles were present in the lungs. This pattern of biodistribution indicates that after intravenous injection, organs of the reticuloendothelial system (like the liver and spleen) are primary sites of nanoparticle accumulation—which is typical for particles in this size range—and also emphasizes the need to study potential pulmonary implications.

In summary, nanoparticles in the 5–15 nm range, exemplified by the MF66 (12 nm) and OD15 (15 nm) SPIONs in this study, are promising for medical imaging and therapy due to their ability to rapidly distribute in the bloodstream, their specific organ localization (particularly in the liver, spleen, and lungs), and their predictable physiological responses. However, the short plasma half-life and the necessity for potential modifications to enhance biocompatibility are important considerations in their design and clinical application.

Nanoparticles in this size range of 5–15 nm offer advantages in terms of cellular uptake and biodistribution, which are critical parameters in cancer therapy. The study underscores that modification of the nanoparticles’ surfaces (for instance, with FA) improves target selectivity by favoring cellular uptake in specific cancer cell lines (i.e., HeLa cells) while potentially providing a protective role against excessive ROS formation, thereby reducing side effects (Guerrero-Florez et al., 2020) [165]. AuNPs in the 5–15 nm range exhibit size-dependent photothermal properties and can be engineered (in terms of both shape and surface chemistry) to optimize their therapeutic performance in PPT. Their small size facilitates effective heat generation and cellular interaction while allowing for additional functionalization (such as FA conjugation) to improve targeting and modulate ROS production. This makes them promising agents for cancer therapy where precise control over thermal effects and ROS generation is paramount.

#### 3.2.4. Enhanced Endosomal Escape and Intracellular Targeting

Another unique advantage of ultrasmall nanoparticles is their potential for efficient endosomal escape and organelle targeting. Sub-15 nm carriers:

- Can disrupt endosomal membranes more effectively due to high local surface energy;
- Have been observed to traffic into the nucleus (especially for particles < 10 nm) without requiring nuclear localization signals;
- Are suitable for mitochondrial or lysosomal targeting when functionalized appropriately.

These properties are especially beneficial for gene delivery, photodynamic therapy, and organelle-targeted chemotherapy, where intracellular localization is critical.

The enhanced cellular uptake and biodistribution of sub-15 nm nanoparticles are central to their effectiveness in cancer therapy. Their small size facilitates efficient internalization, deeper tumor penetration, and favorable pharmacokinetics. However, achieving this balance requires precise control over particle size, shape, and surface chemistry to avoid premature clearance while maximizing therapeutic exposure within tumors.

Huang et al. 2012 [166] provide a complete and comprehensive write-up on synthesis and structural characterization for nanoparticles, with an emphasis on metal and alloy nanomaterials in the size range of approximately 5–15 nm. They employed a size-tunable method to prepare Au@tiopronin nanoparticles across a 2–15 nm range. In particular, these Au@tiopronin nanoparticles have tunable sizes from 2 to 15 nm with identical surface coatings of tiopronin and charge. For gold nanoparticles (AuNPs), achieving ultrasmall sizes (<10 nm) has been linked to enhanced cellular uptake and catalytic performance. Both 2 and 6 nm AuNPs showed high levels of accumulation in tumor tissue in mice after a single intravenous injection, while 15 nm AuNPs accumulated to a lesser extent. Notably, 2 and 6 nm Au@tiopronin nanoparticles were distributed throughout the cytoplasm and nucleus of cancer cells in vitro and in vivo, whereas 15 nm Au@tiopronin nanoparticles were found only in the cytoplasm and formed aggregates.

### 3.3. Stability, Aggregation, and Surface Charge Considerations of Sub-15 nm Nanoparticles

Nanoparticles with diameters below 20 nm exhibit unique surface-dominated behaviors, which present both opportunities and challenges for their use in cancer therapy. While their ultrasmall size offers advantages in tumor penetration and cellular uptake, it also renders them more susceptible to instability, aggregation, and undesired surface interactions in physiological environments. The key physicochemical parameters governing these behaviors are surface energy, zeta potential, and the nature of surface functionalization.

The study by Sierpe et al. (2023) [167] presents a system where AgNPs averaging 15 ± 3 nm are integrated with a βCD-Mel complex. The unique synthesis method and stabilization through exposed functional groups not only ensure the nanoparticles remain within the desired size range but also enhance the effective permeability and potential efficacy of the drug delivery system for cancer therapy.

#### 3.3.1. Colloidal Stability and Thermodynamic Considerations

Colloidal stability is critical for maintaining the dispersion of nanoparticles in aqueous or biological fluids. Sub-15 nm particles, due to their high surface-area-to-volume ratio and surface energy, are inherently thermodynamically unstable and tend to aggregate to minimize surface energy. This is especially pronounced in ionic or protein-rich environments like blood plasma.

Key destabilizing factors include:

- Van der Waals attractions that dominate at the nanoscale;
- Electrolyte-induced screening of surface charges in saline solutions;
- Protein corona formation, leading to bridging and aggregation.

To maintain dispersion, steric and electrostatic stabilization mechanisms are employed:

- Electrostatic stabilization involves generating a high surface charge (zeta potential > |±30| mV) to repel nearby particles.
- Steric stabilization relies on hydrophilic polymers (e.g., PEG, Pluronic, polysaccharides) that form a hydration shell to prevent particle-particle contact.

The researchers reported a one-minute microwave-assisted synthesis in which a fucoidan-enriched fraction extracted from Fucus vesiculosus was used as both the reducing and capping agent. By varying the concentration of fucoidan, they were able to control the nanoparticle size; specifically, monodispersed and spherical NPs exhibit tiny diameters between 5.8 and 13.4 nm for concentrations of fucoidan between 0.5 and 0.05% (*w*/*v*), respectively (Pinto et al., 2020) [168].

Colloidal Stability: The nanoparticles were designed to possess excellent colloidal stability in distinct solutions and culture media, ensuring that the particles remain dispersed and maintain their functional properties under various conditions. Overall, this work illustrates an environmentally friendly (green) and very fast method to produce AuNPs within the 5–15 nm range that are not only size-controlled but also possess promising antitumoral properties coupled with excellent colloidal stability. This synthesis approach could be significant for future biomedical applications, particularly in cancer therapy and imaging (Pinto et al., 2020) [168].

In the study described by Koutsiouki et al., 2017 [169], organophilic iron oxide nanocrystals averaging 7 nm in size (which falls within the 5–15 nm range) were used as a magnetic component. These nanocrystals were co-encapsulated with paclitaxel (PTX) within the oily core of poly(lactide)–poly(ethyleneglycol) (PLA–PEG) nanocapsules. As stated in the article, Paclitaxel (PTX) and organophilic iron oxide nanocrystals of 7 nm average size were co-encapsulated in the oily core.

Colloidal Stability and Controlled Release: The PLA–PEG nanocapsules showed high colloidal stability and sustained drug release properties. This sustained release was further shown to be responsive to an external alternating magnetic field. The responsiveness of these systems can be particularly beneficial when fine control over the drug release profile is desired.

Enhanced Cellular Uptake: The study further improved delivery efficiency by conjugating a cysteine-modified TAT peptide to the particle surface. The TAT peptide, with the sequence HCys-Tyr-Gly-Arg-Lys-LysArg-Arg-Gln-Arg-Arg-Arg-NH_2_, was shown to greatly enhance the cellular uptake of the nanoparticles by cancer cells. This modification translated into a profound improvement of their cytotoxicity against the cancer cells compared to free paclitaxel.

#### 3.3.2. Aggregation Behavior and Its Implications

Aggregation of sub-20 nm nanoparticles results in:

- Loss of size-specific advantages, such as enhanced tumor penetration and renal clearance
- Inaccurate biodistribution, as aggregated particles behave like larger ones (>100 nm)
- Reduced targeting efficacy, due to shielding or loss of surface ligands
- Increased immunogenicity and toxicity, as aggregates can be rapidly recognized by the reticuloendothelial system (RES)

For instance, AuNPs < 5 nm are known to rapidly agglomerate in salt-rich media unless protected by stabilizing agents such as citrate, PEG, or zwitterionic ligands [170]. Similarly, carbon-based nanoparticles like graphene quantum dots and CNTs tend to aggregate due to π-π interactions and hydrophobic forces, unless properly functionalized.

#### 3.3.3. Surface Charge and Zeta Potential

Surface charge, often measured as zeta potential, plays a pivotal role in nanoparticle stability, interaction with cells, and biodistribution. For sub-15 nm nanoparticles:

- Highly positive zeta potentials (>+30 mV) may enhance cellular uptake via electrostatic attraction to negatively charged cell membranes but also increase serum protein adsorption and cytotoxicity.
- Highly negative zeta potentials (<–30 mV) contribute to colloidal stability but may limit membrane interaction and uptake.
- Near-neutral zeta potentials (~±10 mV) often indicate poor colloidal stability and increased risk of aggregation unless sterically stabilized.

In biological media, however, zeta potential may shift due to protein corona formation and ionic screening. Thus, surface functionalization with PEG, zwitterionic groups, or polysaccharides is essential to preserve stability and stealth properties. In the ultrasmall regime, stealth arises from two complementary routes: (i) hydration-layer engineering (e.g., PEG, polysarcosine, poly(2-oxazoline), and zwitterionic polybetaines) that reduces nonspecific protein adsorption; and (ii) pseudo-stealth strategies that intentionally cultivate dysopsonin-rich coronas (e.g., albumin/clusterin/apolipoproteins) to dampen RES recognition. Because complete non-fouling is unrealistic in vivo, tuning the quality of the corona—rather than eliminating it—often yields the most reliable long-circulation behavior for ≤15 nm carriers [171].

Surface Properties:

The nanoparticles possess a zeta potential of 8.11 mV. This moderately positive surface charge likely contributes to colloidal stability and facilitates cellular internalization via endocytosis. Stable surface properties are crucial in ensuring that the nanoparticles remain well-dispersed in biological buffers, thereby enhancing their reliability in drug delivery applications.

#### 3.3.4. Strategies to Improve Stability of Sub-15 nm Nanoparticles

To maintain the desirable size and function of sub-15 nm nanoparticles in complex biological environments, several approaches are employed:

(i) Surface functionalization:
  ○ PEGylation for steric repulsion and protein resistance;
  ○ Ligand grafting (e.g., citrate, polypeptides, poloxamers) to introduce charge or hydrophilicity;
  ○ Zwitterionic coatings to achieve charge neutrality and reduce opsonization.
(ii) Core–shell architectures:
  ○ Encapsulation within liposomes, micelles, or polymeric shells to isolate core particles and enhance dispersion;
  ○ Examples: Lipid-coated quantum dots, micelle-encapsulated AuNPs.
(iii) Buffer optimization:
  ○ Use of low-ionic-strength buffers during storage and formulation;
  ○ Addition of stabilizers like trehalose, sucrose, or PVP during freeze-drying.
(iv) Protein corona control:
  ○ Pre-coating with specific proteins (e.g., albumin) to form a benign corona;
  ○ Engineering anti-fouling surfaces to resist nonspecific binding [172,173].
(v) Stealth by corona design:
  ○ Pre-adsorb benign proteins or use of biomimetic interfaces (e.g., albumin, HDL-mimetics) to favor dysopsonin coronas that reduce RES uptake without sacrificing targeting [171].

The stability of sub-15 nm nanoparticles is intrinsically linked to their surface charge, surface energy, and interaction with the biological environment. Uncontrolled aggregation can negate the benefits of small size, leading to loss of function and increased toxicity. Careful engineering of surface chemistry, charge, and steric stabilization is therefore essential to harness the full therapeutic potential of ultrasmall nanoparticles in cancer therapy.

The paper by Chen et al. (2020) [174] reports magnetic nanoparticles with core sizes of 8–18 nm; many of the properties and techniques described are relevant for nanoparticles in the 5–15 nm range.

To improve stability and biocompatibility, the nanoparticles were coated with polystyrene sulfonic acid, yielding PSS-MNPs. The presence of the coating was confirmed by Fourier-transform infrared spectroscopy, where the detection of SO_3_—groups verified the successful PSS coating. Such surface functionalization is common practice in nanoparticles of this range because it not only prevents agglomeration but also adds functionality for further biomedical applications. Their surface can be appropriately modified (for example, with PSS) to improve stability, dispersibility, and biocompatibility.

In the study by Li et al. (2022) [175], the authors developed polyethylene glycol (PEG)-coated Ag@Au core–shell nanoparticles with an average size of 11 nm, which falls well within this size range of 5–15 nm.

One example of nanoparticles in the 5–15 nm range is the Fe_3_O_4_-based system developed for drug delivery in ovarian cancer cells. In this study, the as-synthesized NPs@carboplatin nanoparticles exhibit an average diameter of 7.88 nm, which falls squarely within the specified range (Song et al., 2019) [85].

Surface Modification: The nanoparticles were PEGylated, meaning they were coated with polyethylene glycol. This modification can improve stability and biocompatibility and reduce nonspecific interactions in biological environments. Additionally, these nanoparticles were further modified with a targeting molecule (the GMT8 aptamer) for specific binding to glioma cells. The study confirmed that the ultraviolet-visible absorption spectra and Fourier transform infrared spectra displayed that GMT8 was successfully conjugated to PSGNPs (Li et al., 2022) [175].

### 3.4. Drug Delivery and Controlled Release from Sub-15 nm Nanoparticles

Sub-15 nm nanoparticles are a powerful platform in pharmaceutical sciences for precise drug delivery and spatiotemporal control of drug release, particularly in oncology. Their ultrasmall size enables enhanced tumor penetration, improved pharmacokinetics, and versatile surface modification for targeted delivery. The combination of passive targeting via enhanced permeability and retention (EPR) effect and active targeting via ligand-receptor interactions allows for precise delivery of therapeutics to tumor tissues while minimizing systemic toxicity.

#### 3.4.1. Passive Targeting via the EPR Effect

Passive targeting exploits the leaky vasculature and poor lymphatic drainage of solid tumors to promote nanoparticle accumulation. Sub-15 nm nanoparticles possess distinct advantages in this context:

- Enhanced tumor penetration: Their small size enables deep diffusion into the tumor interstitium, reaching hypoxic and poorly vascularized regions that are typically inaccessible to larger carriers (>50 nm) [176].
  While EPR remains a central concept, extensive literature shows that its magnitude is heterogeneous across tumor types, sites, and models, and median tumor delivery can be very low in preclinical meta-analyses, which helps explain variable clinical translation. Readers are referred to detailed reviews/meta-analyses for the current state of the field and strategies to enhance delivery (vascular normalization, flow restoration, microenvironment modulation, and active transport). These syntheses collectively emphasize that passive extravasation alone rarely suffices, and that active endothelial processes (transcytosis) likely contribute substantially to tumor entry for many nanocarriers [177,178,179,180].
- Reduced off-target accumulation: Compared to larger particles that accumulate in the liver and spleen, ultrasmall particles can achieve more favorable biodistribution profiles with proper surface functionalization (e.g., PEGylation).
- Prolonged circulation: When coated with stealth polymers or zwitterionic/PEG-alternative brushes, sub-15 nm nanoparticles reduce opsonization and RES clearance, improving effective EPR capture.
  Liposomes and polymeric micelles (~10–20 nm) have demonstrated deeper tumor penetration and enhanced therapeutic efficacy in breast and pancreatic cancer models [8].

Graphene quantum dots and dendrimers under 10 nm have been shown to accumulate efficiently in tumors while clearing renally, offering both efficacy and safety [181].

One of the studies aimed to develop a companion diagnostic for a therapeutic oncology drug (PLX038) by using PEG conjugates that mimic the size and charge of the therapeutic nanocarrier. In this work, the researchers prepared PEG conjugates of the zirconium ligand desferroxamine B (DFB) of similar size and charge to PLX038 (Beckford Vera et al., 2020) [182]. For nanoparticles in the 5–15 nm range, maintaining a defined size range is critical because size strongly influences biodistribution, extravasation, and tumor penetration via the EPR effect. One of the most promising aspects of these PEG nanocarriers is their ability to passively target tumors. The study notes that the small PEG40kDa nanocarriers studied here show properties for passive targeting of tumors that are superior to most nanoparticles (Beckford Vera et al., 2020) [182]. Nanoparticles in the 5–15 nm range are small enough to take advantage of the leaky vasculature in tumors (the EPR effect) and yet large enough to avoid rapid renal clearance, thus increasing tumor exposure.

Combining the experimental and simulated results, several conclusions were made by Chauhan et al. (2012) [140]. The experiments suggest that vascular normalization with anti-angiogenic therapies will only enhance delivery and effectiveness for relatively small therapeutics—including small molecule chemotherapeutics, biologics, and small nanoparticles. Our findings emphasize the importance of size in nanomedicine design by demonstrating that 12 nm particles penetrate tumors better than larger particles. Physical principles dictate that both diffusive and convective penetration—transvascular and interstitial—are faster for smaller particles. Importantly, most normal organs feature non-sinusoid continuous epithelium that may be either fenestrated or non-fenestrated with pore cutoff sizes of up to 6–12 nm, suggesting that 12 nm particles are the smallest that can take advantage of the enhanced permeability and retention (EPR) effect that leads to favorable toxicity profiles for nanomedicine. Indeed, the smallest probe demonstrating selective delivery—a plurality of the injected dose reaching the tumor—through passive EPR is the ~11 nm IgG. While vascular-targeted tumor-penetrating ligands can enhance nanoparticle penetration30 and tumor cell-targeting can improve uptake and retention19, targeting ligands cannot fully overcome tumor penetration barriers made worse by large sizes.

Considering the superior mass flux into tumors and long circulation times for small nanoparticles, along with the large number of patients receiving normalizing anti-angiogenics, small size may represent an important new design constraint for anticancer nanomedicine.

#### 3.4.2. Active Targeting via Surface Ligand Functionalization

Active targeting involves the attachment of tumor-specific ligands to the nanoparticle surface, enabling receptor-mediated uptake by cancer cells. The high surface-to-volume ratio of sub-15 nm nanoparticles facilitates dense functionalization with ligands such as:

- Small molecules (e.g., folic acid);
- Peptides (e.g., RGD, TAT);
- Antibodies or antibody fragments;
- Aptamers or nucleic acids.

This enhances:

- Selective accumulation in tumor cells overexpressing specific receptors (e.g., folate receptor, integrins, EGFR);
- Receptor-mediated endocytosis, improving cellular uptake and intracellular drug delivery;
- Minimized off-target toxicity through selective targeting;
- Transcytosis-enabled targeting (endothelial crossing): Beyond receptor-mediated cellular uptake, receptor-mediated transcytosis across endothelial cells is increasingly recognized as a dominant entry route for nanoparticles into tumors. In mouse and human samples, quantitative imaging and modeling indicate that the vast majority (often > 90%) of nanoparticles enter tumors via active trans-endothelial transport, rather than through static inter-endothelial gaps alone. For sub-15 nm carriers, size, curvature, and ligand display can favor caveolae-/vesicle-mediated uptake and vesiculo-vacuolar organelle (VVO) transport, aligning with classic observations of VEGF-induced hyperpermeability. Design levers include ligands to receptors involved in endothelial trafficking (e.g., transferrin receptor, ICAM-1, albumin/GP60 pathways, integrins), zwitterionic/stealth coronas that reduce nonspecific adhesion yet preserve receptor binding, and compact particle sizes that fit vesicular pathways [183,184].

Examples:

- 10–15 nm AuNPs functionalized with RGD peptides showed increased uptake in integrin-positive tumors and improved photothermal therapy outcomes [185]. The challenges associated with targeting ligands are the target expression heterogeneity, off-target binding, and immunogenicity of larger ligands. These necessitate quantitative control of surface ligand density and size distribution as CQAs, confirmed with orthogonal analytics.

As noted by Wang et al. (2007) [186] nanometer-sized particles have functional or structural properties that are not available from either molecular or macroscopic agents. In this size range, several key advantages become evident, like enhanced targetability. When these nanoparticles are linked with biotargeting ligands such as monoclonal antibodies, peptides, or small molecules, they can selectively accumulate in malignant tumors. This targeting is critical for achieving high affinity and specificity in cancer therapy. The small size (5–15 nm) assists in tissue penetration and in evading some biological barriers, further improving their effectiveness as targeted delivery vehicles.

Folate-conjugated dendrimers < 10 nm delivered doxorubicin with high efficiency to folate receptor-positive ovarian cancer cells [187].

In the study by Lee et al. (2022) [188], one of the synthesized nanoparticles—the folic acid and chitosan-functionalized gold nanorods (FACS-R)—had a transverse length of 13.1 ± 1.8 nm, which lies within the upper portion of the 5–15 nm range. A notable application of these nanoparticles is their ability to encapsulate anticancer agents. In the context provided, various agents such as docetaxel, paclitaxel, and diallyl disulfide were loaded into both FACS-R and the larger FACS-T. The efficiency of drug encapsulation can be influenced by the nanoparticle surface area available for drug loading—a characteristic that is enhanced in the sub-15 nm range nanoparticles. In particular, paclitaxel-encapsulated FACS-R and FACS-T showed the highest percentages of early and late apoptosis on HeLa cells (Lee et al., 2022) [188]. Nanoparticles of this size can more easily traverse biological barriers and may provide enhanced biodistribution and tumor targeting. The use of targeting ligands (folic acid in this case) along with chitosan functionalization improves cellular uptake, particularly in cancer cells that overexpress folate receptors. The authors conclude, Collectively, the triangular silver nanoplates were more effective than the gold nanorods for PTT. These as-prepared nanoparticles have remarkable features and will become promising future nanomedicine, emphasizing the growing role of well-sized nanoparticles in cancer therapy.

Wu et al. (2018) [189] illustrate several important features of nanoparticles in the 5–15 nm range by considering their magnetic core properties. In the study, the authors synthesized ultrasmall superparamagnetic iron oxide (USPIO) nanoparticles, where they reported that the average diameter of Fe_3_O_4_ nanoparticles was 8–10 nm. This size range is important because nanoparticles with diameters from 5 to 15 nm typically exhibit superparamagnetism—a property that is critical for applications in magnetic resonance imaging (MRI).

Superparamagnetic nanoparticles do not retain residual magnetization after removal of an external magnetic field, making them suitable as contrast agents for MRI because they are less likely to cause long-term magnetic aggregation.

Wu et al. developed core–shell silica NPs (Cornell dots, or C’ dots) composed of encapsulated Cy5 near-infrared (NIR) dyes, a protective PEG layer, the drug exatecan conjugated via a cathepsin-B cleavable linker, and folic acid targeting molecules [190]. The multifunctional C’ dot particles contained on average 21 exatecan and 13 folic acid molecules, while maintaining a compact HD of about 6.4 nm. As a result of their ultrasmall size and protein-resistant surface chemistry, the C’ dots showed efficient renal clearance and no significant retention in any critical organ. A competitive cell-binding study was conducted to assess the binding affinity of the targeted C’ dots toward corresponding folic acid receptors. The findings revealed a strong multivalency effect, leading to a 40-fold enhancement in binding affinity relative to free folic acid (IC_50_ values of 0.4 nM vs. 16.4 nM). Systematic studies were conducted to compare the performance of the targeted C’ dot formulation with an antibody drug conjugate using both 3D tumor spheroid models and xenograft animal tumor models. The results indicated that the C’ dots exhibited significantly deeper penetration within 3D cell-line-derived spheroids. Additionally, the particles demonstrated enhanced efficacy in both cell-line-derived and patient-derived in vivo tumor xenograft models.

#### 3.4.3. Controlled Drug Release Mechanisms

Controlled release from sub-15 nm carriers ensures that therapeutics are:

- Protected during circulation
- Released specifically at the tumor site or within cancer cells
- Responsive to internal or external stimuli

Mechanisms of drug release include:

- pH-responsive systems: Exploit the acidic tumor microenvironment or endosomal compartments to trigger release (e.g., acid-labile bonds, protonation of polymer backbones).
- Redox-responsive systems: Use disulfide linkages that are cleaved in the presence of elevated intracellular glutathione.
- Enzyme-sensitive carriers: Release payload in response to matrix metalloproteinases (MMPs) or cathepsins overexpressed in tumors.
- Photothermal and photodynamic triggers: Light-responsive nanoparticles (e.g., AuNPs, GQDs) release drugs upon NIR irradiation [191].

Examples:

- Iron oxide nanoparticles < 15 nm functionalized with cathepsin-cleavable linkers for enzyme-triggered doxorubicin release [192].

One example of nanoparticles in the 5–15 nm range is found in the controlled drug release system developed by Catarata et al. (2020) [193]. In their study, poly(acrylic acid) (PAA) was coupled with gemcitabine (GEM) to form PAA-GEM conjugate nanoparticles that were characterized in different environments. In the dry state, these nanoparticles exhibited a size of approximately 12 nm, which falls squarely within the 5–15 nm range (Catarata et al., 2020, p. 2887) [193]. Thus, the PAA-GEM nanoparticles, especially their dry state size of about 12 nm, exemplify how nanoparticles in the 5–15 nm range can be engineered for controlled drug release with efficient cell uptake and high stability. This case illustrates the potential of designing nanoparticles with precise size control to optimize therapeutic outcomes in targeted drug delivery applications.

Nanoparticles in the 5–15 nm range, as exemplified by the 6.4 nm nanoparticles described by Huang et al. (2019) [194], offer several advantages. Their small size, pH-sensitive behavior, surface charge modulation, and low cytotoxicity make them an effective platform for enhancing the delivery and efficacy of chemotherapeutic agents. Such design parameters contribute to improved cellular uptake in acidic tumor environments, potentially leading to better therapeutic outcomes with reduced side effects.

One example of a nanoparticle system falling in the 5–15 nm range is the CD44-targeted glutathione-sensitive hyaluronic acid-mercaptopurine (HA-GS-MP) prodrug described by Qiu et al. (2017) [195]. In this work, the authors report that:

- The prodrug is prepared by conjugating 6-mercaptopurine (6-MP) to 50 kDa hyaluronic acid via a carbonyl vinyl sulfide linker, resulting in a drug conjugate with a 6-MP content of 6.9 wt%.
- The HA-GS-MP nanoparticles exhibit excellent water solubility with a hydrodynamic size of ca. 15 nm, which places them at the upper end of the specified 5–15 nm range.

The stimuli-responsive drug release mechanism can help to ensure that the active therapeutic agent is delivered in a controlled manner within the target tissue.

In summary, the HA-GS-MP system provides a comprehensive example of how nanoparticles sized between 5 and 15 nm may be designed for targeted, stable, and controlled drug delivery applications in cancer therapy—combining favorable physicochemical properties with enhanced therapeutic efficacy.

#### 3.4.4. Combination Therapies and Theranostics

Sub-15 nm nanoparticles are ideally suited for combination therapies, where multiple agents (e.g., chemotherapy + siRNA, chemo + photothermal therapy) are co-delivered for synergistic effects. Additionally, their small size and surface modifiability make them ideal for theranostic applications, combining diagnostic imaging with therapeutic payloads in a single platform.

Examples:

- Sub-10 nm carbon dots delivering both paclitaxel and siRNA while enabling fluorescence imaging
- Gold nanoshells < 15 nm used for combining photothermal therapy and drug delivery under image guidance

Sub-15 nm nanoparticles represent a transformative tool in precision drug delivery and controlled release. Their small size enables superior tumor penetration, efficient passive and active targeting, and the ability to release therapeutic agents in response to the tumor microenvironment. Rational design of these carriers allows for highly effective cancer therapy with minimized systemic toxicity, laying the foundation for future smart and personalized nanomedicine platforms.

The combination of photothermal therapy and chemotherapy by Wang et al. [196] was shown to exhibit evidence of synergistic effects on killing 4T1 breast cancer cells in both in vitro cell-killing assays and in vivo tumor treatment studies. This synergy is a promising feature of theranostic agents, as it allows for multiple treatment modalities to work together to improve therapeutic outcomes. These nanoparticles in the 5–15 nm range, as exemplified by the PAA-Ni0.85Se nanoparticles, offer a blend of rapid body clearance, strong optical absorption in the NIR region, high photothermal conversion efficiency, and the ability to serve as platforms for responsive drug delivery and synergistic cancer therapy.

Magnetite nanoparticles (MNPs) have been demonstrated to offer multifunctional applications in both imaging and therapy. These MNPs exhibit enzyme-mimicking capabilities. Specifically, they can mimic the activity of horseradish peroxidase (HRP) by catalyzing the decomposition of hydrogen peroxide (H_2_O_2_) to generate reactive oxygen species (ROS). This catalytic activity is crucial for both antibacterial and anti-tumor applications. The study noted that (Zhang et al., 2013) [197] MNPs demonstrated to possess enzyme-mimicking activity in different pH values, and the activity was dependent on the size of the MNPs: the smaller the size, the higher the activity.

- In vivo studies in mice bearing subcutaneously implanted HeLa cells showed significant tumor inhibition. For example, an approximately 99% tumor inhibition ratio was shown by the combination of MNPs and H_2_O_2_ after treatment for 17 days. This highlights how nanoparticles in this size range can be optimized for effective cancer therapy.
- These nanoparticles also serve as efficient magnetic resonance (MR) imaging contrast agents. In vitro and in vivo studies indicated that 13 nm MNPs could be used as highly sensitive T_2_-weighted MR imaging agents. Their relaxivity was determined to be r_2_ = 104 s^−1^·mM^−1^, making them suitable for tracking and targeting tumors via MR imaging. The study also reports that the MR signal was much more negative, and the intensity was significantly diminished with the increase in the concentration of 13 nm MNPs in vitro.
- In vivo, tumors were clearly visualized with a 3-fold decrease in MR signal intensity at the tumor site after 24 h following treatment, indicating successful targeting and accumulation at the desired location.

Based on the provided information (Hao et al., 2013) [198], nanoparticles in the range of 5–15 nm—illustrated here by 12 nm Fe_3_O_4_ (magnetite) nanoparticles—exhibit several key features that make them attractive for biomedical applications. For the 12 nm nanoparticles, the ligand exchange resulted in a small hydrodynamic size of 14 nm. This precise size range is critical because it enables unique magnetic properties. For instance, a high T1 relaxivity of 17.8 mM^−1^·s^−1^ was achieved and, owing to a high saturation magnetization of 77.8 emu g^−1^, the particles demonstrated a high T2 relaxivity of 220 mM^−1^·s^−1^ in MRI phantom experiments. The dual capability to enhance both T1 and T2 contrasts suggests their efficacy as multimodal imaging agents.

In summary, nanoparticles in the range of 5–15 nm, such as the 12 nm Fe_3_O_4_ nanoparticles as described above, are engineered to maintain high magnetic performance, water colloidal stability, and excellent biocompatibility. Their advantageous small size allows for optimized relaxivity values for both T1 and T2 MRI, as well as effective use in magnetic hyperthermia, underscoring their potential for use in integrated diagnostic and therapeutic (theranostic) applications.

In the study by Starha et al. (2014) [199], a one-pot synthesis was used to prepare superparamagnetic maghemite-based nanoparticles that are coated with 4-aminobenzoic acid (PABA). These PABA@FeNPs serve as nanocomposites that can be exploited as magnetic carriers for drug delivery, a feature particularly promising for oncological applications. The authors describe that after synthesis, the nanoparticles were well-dispersed and had an average size of 13 nm. This size lies well within the 5–15 nm range, making them an ideal candidate for applications where both high surface area and effective magnetic response are required. The narrow size distribution is critical as it ensures uniform magnetic properties as well as consistent behavior in biological environments. Furthermore, the study details that the PABA@FeNPs were further functionalized with activated species of highly in vitro cytotoxic cis-[PtCl2(3Claza)2] or cis-[PtCl2(5Braza)2]—complexes that are analogous to cisplatin. The overall approach effectively integrates the magnetic carrier system with highly cytotoxic drugs, demonstrating the potential for targeted drug delivery.

### 3.5. Sub-15 nm Nanoparticles for Blood–Brain Barrier Penetration

**Figure 3:**
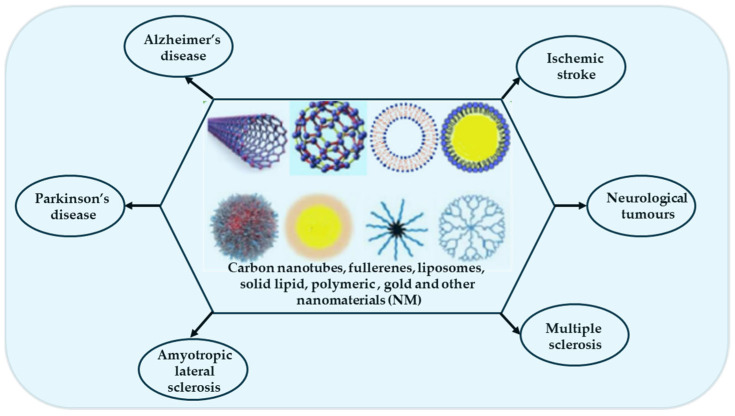
Different types of NPs and their application in neurological disorder treatment and management. Adapted from Khwaja Salahuddin Siddiqui (2018) [200], Copyright@2018, Elsevier.

Treating neurological disorders such as brain tumors, Alzheimer’s disease, Parkinson’s disease, and glioblastoma remains one of the greatest challenges in pharmaceutical sciences due to the presence of the blood–brain barrier (BBB)—a highly selective, tightly regulated endothelial interface that restricts most systemically administered therapeutics from entering the central nervous system (CNS). Nanoparticles under 15 nm in diameter have emerged as promising carriers capable of crossing the BBB, enabling both diagnostic imaging and therapeutic delivery to the brain. To overcome these issues, various formulations of NMs/NPs have shown extensive and promising applications in drug delivery against neurological disorder treatment and management (Figure 3).

#### 3.5.1. The Blood–Brain Barrier: A Key Challenge in Neuropharmacology

The BBB consists of:

- Tight junctions between endothelial cells
- Efflux pumps (e.g., P-glycoprotein)
- Astrocytic endfeet and pericytes

These features limit passive diffusion and actively exclude most small-molecule and macromolecular drugs. However, ultrasmall nanoparticles (<15 nm) are increasingly recognized for their potential to overcome these obstacles via multiple mechanisms.

#### 3.5.2. Size-Dependent BBB Penetration

Sub-15 nm nanoparticles possess characteristics that favor BBB traversal:

- Size within or below endothelial pore limits (~20 nm) enables paracellular or transcytotic transport.
- Low steric hindrance supports uptake via adsorptive- or receptor-mediated transcytosis.
- Increased diffusivity compared to larger particles enhances passage through dense brain parenchyma after BBB crossing.

Studies have demonstrated that AuNPs, polymeric micelles, and quantum dots in the 5–20 nm range successfully cross the BBB, depending on surface chemistry and formulation [201].

The nanoparticles described by Ashrafizadeh et al., 2020 [202] are extremely small carbon dots (CD) and have a small size, less than 10 nm, enabling penetration into the BBB. Their reduced dimensions not only enhance their ability to diffuse in biological environments but also allow them to penetrate challenging biological barriers such as the blood–brain barrier (BBB). The review underlines the use of CDs in the development of novel neurodrug delivery systems. Their small size (<10 nm) is specifically noted to be advantageous as it enables penetration into the BBB. This unique capability is critical for reaching the central nervous system (CNS), which is often difficult to access due to the protective nature of the BBB. This property allows nanoparticles in the 5–15 nm size range to be effective carriers for drugs targeting neurological disorders.

Overall, nanoparticles in the 5–15 nm range, exemplified here by carbon dots, possess a combination of small size, tunable optical properties, excellent photostability, and high biocompatibility. These characteristics, coupled with straightforward synthetic protocols, render them highly suitable for advanced biomedical applications, including neurodrug delivery, bioimaging, theranostics, and sensitive detection systems.

Nanoparticles with sizes in the range of 5–15 nm have attracted significant attention in biomedical research due to their unique physicochemical properties and enhanced biological interactions. One notable example is found in the development of extremely small gadolinium oxide-based nanoparticles designed specifically for theranostic applications in glioblastoma (GBM). The study described in the context synthesized poly(acrylic acid) (PAA)–stabilized gadolinium oxide nanoparticles that were further modified with reductive bovine serum albumin (rBSA). The resulting nanoparticles, denoted as ES-GON-rBSA, had a hydrodynamic diameter (dh) of approximately 13.4 nm, placing them squarely within this optimal size range (Shen et al., 2020) [203].

Enhanced Blood–Brain Barrier (BBB) Permeability: One of the major challenges in the treatment of brain tumors, such as GBM, is crossing the BBB. It is indicated in the study that “smaller particle size can lead to higher blood–brain barrier (BBB) permeability,” which is why many successful nanomaterials for GBM therapy are engineered to be less than 14 nm. This is particularly important because most previously reported BBB-crossable nanomaterials for GBM therapy are larger than 24 nm, potentially limiting their effective transport and accumulation in the brain, according to Shen et al. (2020) [203].

One example of nanoparticles falling in the 5–15 nm range is the engineered bacterial membrane biomimetic nanodrug delivery system described by Zhao et al. (2024) [204]. Although the study focused on developing a targeted system for glioma treatment, several of its findings help illustrate the features and advantages of nanoparticles in this size range.

Enhanced targeting and biological interactions:

- The engineered nanoparticles are designed for targeted drug delivery. The surface modification with Angiopep-2, in particular, improved the ability of the nanoparticles to cross the blood–brain barrier (BBB) and target the inflammatory microenvironment of glioma. The study reported a higher uptake by activated neutrophils (uptake efficiency increased from 24.9% for the uncoated system to 31.1% for ANG-2 EM@PPC), which is important for hitchhiking on these cells to deliver drugs effectively (Zhao et al., 2024) [204].
- Furthermore, the nanoparticles influenced cellular processes by altering the death pathway of neutrophils from neutrophil extracellular traps-osis (NETosis) to apoptosis. This modification was confirmed by both Western blot and flow cytometry (with apoptotic body production reaching as high as 77.7%), suggesting that nanoparticles in this size range can be finely tuned to affect cellular responses.
- In animal models of in situ glioma, all formulations of the engineered nanoparticles (including those with ANG-2 modification) demonstrated effective distribution to brain tissue with higher affinity and internalization by neutrophils at the tumor site, compared to the control (DiR group) (Zhao et al., 2024) [204].

#### 3.5.3. Strategies for Enhancing BBB Crossing

While size is a critical parameter, surface functionalization greatly improves the efficiency and specificity of BBB crossing:

(i) Passive mechanisms:
  - Nanoparticles with hydrophobic coatings, zwitterionic surfaces, or low protein adsorption may exploit transient BBB permeability or adsorptive transcytosis.
(ii) Active targeting mechanisms:
  - Receptor-mediated transcytosis (RMT):
    ▪ Transferrin receptor (TfR): Used by functionalizing NPs with transferrin, lactoferrin, or TfR-binding antibodies;
    ▪ Low-density lipoprotein receptor (LDLR): Targeted using apolipoprotein E (ApoE) or mimetic peptides;
    ▪ Insulin receptor targeting brain tumor and neurodegenerative drug delivery.

Example:

- 10–15 nm PEGylated AuNPs conjugated with transferrin showed efficient accumulation in glioma tissue in vivo and enabled imaging and drug delivery [205].

(iii) Carrier-mediated transport (CMT):
  - Exploits glucose, amino acid, or peptide transporters to carry drug-loaded ultrasmall NPs across the BBB.

#### 3.5.4. Imaging and Diagnostic Applications

Sub-15 nm nanoparticles offer excellent properties for neuroimaging:

- Iron oxide nanoparticles (<15 nm) act as MRI contrast agents (T1 or T2) for early detection of brain tumors or lesions.
- Graphene quantum dots and carbon dots (<10 nm) provide fluorescent and photoacoustic signals, enabling real-time imaging of BBB passage and brain accumulation.
- Radiolabeled gold nanoparticles have been explored for PET imaging in neuro-oncology.

McNelles et al. describe a fifth-generation aliphatic polyester dendrimer that was modified first by functionalizing the periphery with vinyl groups and by incorporating a dipicolylamine Tc(I) chelate into the core. The dendrimer was then PEGylated using three different molecular weight monomethoxy poly(ethylene glycol) (mPEG) reagents—mPEG160, mPEG350, and mPEG750—through thiol-ene click chemistry. Notably, when using mPEG750, the dendrimer was found to be molecularly dispersed in water with a hydrodynamic diameter of 9.2 ± 2.1 nm, thereby fitting within the 5–15 nm nanoparticle range (McNelles et al., 2015) [206].

This size is significant because nanoparticles within this range often exhibit advantageous in vivo properties. In the study, the 9.2 nm PEGylated dendrimer was further radiolabeled with [(99m)Tc(CO)_3_(H_2_O)_3_]^+^ purified to a radiochemical purity of over 99%, and then used in imaging studies. Imaging showed that these nanoparticles could circulate in the bloodstream for an extended period (up to 24 h) and selectively accumulate in H520 xenograft tumors, where they were successfully visualized by single-photon emission computed tomography (SPECT). This example underscores the potential of nanoparticles of this size to serve as platforms for tumor-targeted molecular imaging probes and potentially for therapeutic applications.

#### 3.5.5. Therapeutic Applications in Neurological Diseases

(i) Brain tumors (e.g., glioblastoma):
  - Sub-15 nm NPs loaded with doxorubicin, temozolomide, or siRNA have shown efficient accumulation in intracranial tumors, especially when actively targeted.
(ii) Neurodegenerative diseases:
  - NPs delivering siRNA, antioxidants, or neuroprotective peptides (e.g., nerve growth factor) can halt or slow the progression of Alzheimer’s or Parkinson’s disease.
  - Graphene quantum dots have shown ROS scavenging ability and Aβ aggregation inhibition, offering therapeutic benefit in Alzheimer’s models [207].
(iii) Stroke and neuroinflammation:
  - Anti-inflammatory drugs and neuroprotective agents delivered via sub-15 nm carriers reduce BBB breakdown and oxidative damage post-stroke.
  - Nanoparticles of this size have unique advantages for drug delivery, particularly when targeting central nervous system tumors. In the referenced study, investigators used polyamidoamine dendrimers— nanoparticles whose sizes are tightly controlled due to their multigenerational structure (with sizes increasing by only 1 to 2 nm per successive generation)—to explore how size affects transvascular delivery into malignant glioma cells (Sarin et al., 2008) [208]. Although the study specifically focused on the ability of these nanoparticles to traverse the blood-brain tumor barrier, the findings are informative for the broader 5–15 nm range.
  - The study demonstrated that nanoparticles must be below a certain critical size to effectively permeate the pore structures of the blood-brain tumor barrier. According to the authors, the intravenously administered functionalized dendrimers, less than approximately 11.7 to 11.9 nm in diameter, were able to traverse the pores of the blood-brain tumor barrier of RG-2 malignant gliomas, while larger ones could not.
  - Given that the 5–15 nm range spans both sub-threshold (for example, those around 5–11 nm) and suprathreshold (above 11.9 nm) sizes, nanoparticles at the lower end would be more likely to cross the barrier.
  - Furthermore, within the subset of permeable nanoparticles (those below roughly 11.7–11.9 nm), having long blood half-lives was crucial for effective accumulation within glioma cells. This means that even if a nanoparticle is small enough to cross the barrier, its ability to remain in circulation for sufficient periods is essential for it to localize within target tumor cells. In practical terms, nanoparticles in the 5–15 nm range that are engineered to have both an optimal size (ideally below the 11.7–11.9 nm threshold) and extended circulation times are more promising as vehicles for targeted drug delivery

#### 3.5.6. Safety and Clearance Considerations

The small size of sub-15 nm nanoparticles also facilitates:

- Renal clearance, reducing the risk of long-term accumulation in the CNS;
- Minimized immune activation, especially with stealth coatings (e.g., PEG, zwitterionic ligands);
- Lower cytotoxicity, especially for carbon-based or polymeric systems.

However, thorough safety profiling is essential, as surface reactivity and metal core composition (e.g., in quantum dots) may pose toxicity risks if not properly stabilized.

In summary, sub-15 nm nanoparticles represent a breakthrough in neuropharmaceutical design, offering the unique capability to cross the blood–brain barrier, distribute effectively in brain tissue, and deliver diagnostic or therapeutic agents with precision. Their small size, surface modifiability, and compatibility with biological transport mechanisms make them powerful tools for tackling challenging CNS diseases such as glioblastoma, Alzheimer’s, and Parkinson’s disease. Future development must continue to focus on balancing efficacy, targeting, and safety for clinical translation.

One example of nanoparticles at a similar scale is the sub-5 nm silver sulfide nanoparticles (Ag_2_S-NP) described by Hsu et al. (2023) [209]. Although the paper specifically reports sub-5 nm Ag_2_S-NP, many of the principles and benefits discussed also apply to nanoparticles in the 5–15 nm range. A key benefit noted in the study was that all syntheses were conducted using biocompatible reagents in the aqueous phase and under ambient conditions. Furthermore, after fulfilling their diagnostic and therapeutic roles, the nanoparticles “degrade into small components for excretion, and the authors observed a gradual decrease in AgPCPP retention in tissues over time with no signs of acute toxicity. Nanoparticles sized between 5 and 15 nm are often sought after because their relatively small dimensions can support more effective clearance from the body, potentially reducing long-term toxicity.

AgNPs are widely used in industrial and household applications, arousing concern regarding their safety in humans (Kim et al., 2019) [210]. Being in the nanometer range means they can easily interact with and penetrate biological tissues, which necessitates thorough safety and toxicity assessments. Their small dimensions are a double-edged sword as they lend to beneficial applications (like antimicrobial uses) but also potential health risks.

Kim et al. (2019) [210] investigated the long-term toxicity by conducting several toxicity tests and transcriptomic analysis. They reported that the structure and function of liver tissue were disrupted due to a single exposure to cAgNPs. One of the key findings was that an “in vivo comet assay showed unrepaired genotoxicity in liver tissue until 4 weeks after a single injection, suggesting a potential carcinogenic effect of cAgNPs. This outcome highlights the importance of understanding nanoparticle interactions at the molecular level, even after a single exposure.

In summary, the study by Kim et al. highlights that AgNPs in the 5–15 nm range can exhibit significant biological activity. They are widely integrated into everyday products but have the potential to disrupt tissue structure and induce long-lasting genotoxic effects, making the assessment of their safety paramount.

### 3.6. Applications of Sub-15 nm Nanoparticles in Vaccines and Immunotherapy

Sub-15 nm nanoparticles are emerging as versatile tools in modern vaccine design and immunotherapy, owing to their unique size, surface properties, and ability to modulate immune responses. Their ultrasmall size enables efficient lymphatic drainage, interaction with immune cells, and intracellular delivery of antigens, adjuvants, or immunomodulatory molecules. These properties make them particularly valuable in developing next-generation cancer vaccines, infectious disease vaccines, and immune-based therapies.

The study by Zhu et al. (2020) [211] demonstrates that nanoparticle size can determine the activation of innate immune signaling pathways. An interesting functional consequence of these size-dependent effects was demonstrated when the ultrasmall nanoparticles (Au4.5) were used as vaccine adjuvants. The study noted that these particles could function as vaccine adjuvants to markedly enhance ovalbumen (OVA)-specific antibody production in an NLRP3-dependent pattern, showcasing their potential utility in immunotherapy.

In summary, nanoparticles within the 5–15 nm size range can be expected to have dual immunomodulatory roles:

Nanoparticles toward the lower end (around 5–10 nm) are highly effective in directly penetrating cells, inducing ROS production, degrading LC3, and, thus, activating the NLRP3 inflammasome; those toward the higher end (approaching 15 nm) tend to activate the NF-κB pathway instead.

This size-dependent behavior is critical for tailoring nanoparticle design for efficient immunotherapy and other biomedical applications, as it underscores the importance of optimizing nanoparticle size to achieve the desired activation of specific innate immune responses.

Nanoparticles in the 5–15 nm range, specifically recombinant ferritin-based neoantigen nanoparticles (neoantigen-FNs), are described in the study by Zheng et al. (2024) [212]. The study reports that the neoantigen-FNs vaccine induced a greater quantity and quality of antigen-specific CD8+ T cells. This robust activation of T cells plays a key role not only in the significant growth control of multiple tumors but also in the dramatic inhibition of melanoma metastasis and even results in the regression of established tumors.

In summary, the recombinant ferritin nanoparticles described in the study are exemplary of how nanomaterials in the 5–15 nm range can be harnessed to achieve targeted delivery, robust immune activation, and strong therapeutic outcomes while maintaining a favorable safety profile.

Based on a study by Kang S. et al. (2017) [213], an example of nanoparticles in the range of 5–15 nm being used is the use of gold nanoparticles (GNPs) as vaccine carriers. In the referenced study, GNPs with nominal diameters of 7, 14, and 28 nm were synthesized, and when conjugated with recombinant ovalbumen (OVA) as a model antigen, they yielded particles with hydrodynamic diameters of approximately 10, 22, and 33 nm, respectively. For nanoparticles in the 5–15 nm range, the 7 nm GNPs—resulting in ~10 nm OVAGNPs—are most relevant.

According to the study, these ~10 nm (from 7 nm GNPs) particles were evaluated for several key parameters:

1. Cellular Uptake: The authors noted a size-dependent increase in cellular uptake by dendritic cells (DCs) and subsequent T-cell cross-priming and activation. This indicates that even within the smaller range, the nanoparticles are actively taken up by immune cells.
2. Lymph Node Delivery: Upon injection into a mouse footpad, it was observed that both 22 and 33 nm OVAGNPs showed much higher delivery efficiency to draining LNs than did 10 nm OVA-GNPs. Thus, although the 10 nm particles (derived from 7 nm GNPs) are capable of local cell uptake, their efficiency in reaching lymph nodes (and hence in orchestrating systemic immune responses) appears limited compared to somewhat larger sizes.
3. Immune Response Threshold: The study concludes that the size threshold for induction of potent cellular responses and T-cell poly-functionality by GNPs lies between 10 nm and 22 nm. This suggests that while nanoparticles in the 5–15 nm range (with the example particle having an effective size of ~10 nm) can induce immune responses, they may fall below the optimal threshold for inducing maximum CD8^+^ T-cell activation. Indeed, an ex vivo restimulation assay revealed that frequencies of OVA-specific CD8+ T cells were higher in mice immunized with 22 and 333 nm OVA-GNPs than in those immunized with 10 nm OVA-GNPs.

In summary, nanoparticles within the 5–15 nm range—exemplified by the 7 nm GNPs that yielded ~10 nm hydrodynamic particles—exhibit effective cellular uptake by dendritic cells but are less efficient in lymph node delivery and subsequent stimulation of robust CD8^+^ T-cell responses compared to slightly larger particles. This work underscores the importance of nanoparticle size as a critical design parameter in the development of nanoparticle-based vaccines, where sizes below a certain threshold (in this case, between 10 nm and 22 nm) might not maximize immunogenicity.

#### 3.6.1. Rational for Sub-15 nm Nanoparticles in Immunomodulation

The immune response to vaccines and immunotherapies is governed by how effectively antigens are presented to dendritic cells (DCs), macrophages, and T cells. Nanoparticles < 15 nm offer several critical advantages for these applications:

- Efficient lymph node targeting: Nanoparticles below 20–30 nm drain rapidly through lymphatic capillaries and accumulate in lymph nodes, where antigen-presenting cells (APCs) reside [214].
- Improved uptake by dendritic cells: Smaller particles are preferentially internalized by DCs via clathrin- and caveolae-mediated endocytosis, facilitating antigen processing and presentation.
- Surface engineering flexibility: High surface area allows for co-loading of antigens, adjuvants (e.g., CpG, MPLA), and targeting ligands (e.g., mannose) on the same particle.
- Enhanced antigen stability and cross-presentation: Sub-15 nm particles protect protein or peptide antigens from degradation and promote MHC class I cross-presentation for cytotoxic T cell activation.

#### 3.6.2. Vaccine Delivery Applications

Sub-15 nm nanoparticles have been applied in the design of nanovaccines for both infectious diseases and cancer. These nanovaccines are typically composed of:

- Antigen-loaded polymeric micelles, dendrimers, liposomes, or LNPs
- Surface-conjugated peptides, proteins, or mRNA
- Immunostimulatory adjuvants, such as toll-like receptor (TLR) agonists

Key features of sub-15 nm nanovaccines:

- Rapid lymph node trafficking, which enhances immune priming
- Controlled release of antigen, mimicking natural pathogen exposure
- Reduced systemic toxicity compared to soluble adjuvants

Example:

- LNPs ~15 nm in size were used in mRNA COVID-19 vaccines, demonstrating high efficiency in delivering nucleic acid vaccines and triggering robust humoral and cellular immunity [39].

One example of nanoparticles in the 5–15 nm range is the recombinant ferritin-based neoantigen nanoparticles (neoantigen-FNs) described in the study by Zheng et al. (2024) [212].

Nanoplatform-based formulations can strongly prolong immune stimulation as well as the controlled and sustained release of encapsulated cargos. Careful selection of the components used in constructing a vaccine’s nanoformulation can ensure the slow release of the encapsulated cargos over time in a controlled manner, which has been shown to prolong the elevation of antibody and results in the production of a high level of effector memory T cells [215,216]. The size range of nanoplatforms is another factor that can enable improved delivery of a vaccine-effective agent. The nanoscale ranges of nanocarriers allow for efficient lymphatic drainage into the lymphoid organs where antigen uptake and processing occur [214,217,218]. In one example application of this effect, iron oxide-zinc oxide nanostructures with a core–shell structure were used for the delivery of carcinoembryonic antigen [219]. These NPs had an average size of 15 nm, enabling them to effectively travel into lymph nodes. Once at their destination to the lymph nodes, the NPs could be taken up by DCs for processing to extract specific immunity against the antigen. When this nanoformulated vaccine was administered to mice, there was a 10-fold increase in splenic CD8+ T cells secreting IFN-γ (proinflammatory cytokine commonly correlated with the activation of cell-mediated immunity). Although the nanoformulated vaccine could not completely eradicate the tumor, vaccination with iron oxide-zinc oxide nanostructures delayed tumor growth and extended mean survival from 10.5 to 19.5 days. An added advantage of these nanoformulations was their inherent ability to be used for MRI.

#### 3.6.3. Cancer Immunotherapy

In cancer immunotherapy, sub-15 nm nanoparticles are being engineered to:

- Deliver tumor-associated antigens (TAAs) or neo-antigens to APCs;
- Stimulate cytotoxic CD8^+^ T-cell responses;
- Reprogramming the tumor microenvironment (TME) by delivering immunomodulators;
- Co-deliver checkpoint inhibitors or siRNA targeting immune suppressive genes (e.g., PD-L1, IDO).

(i) Tumor antigen delivery:
  - Dendritic cell-targeted nanoparticles < 15 nm carrying TAAs (e.g., gp100, TRP2 peptides) induced tumor-specific T-cell responses and delayed tumor progression in melanoma models [220].
(ii) mRNA-based cancer vaccines:
  - Sub-15 nm LNPs encapsulating mRNA coding for tumor neoantigens have shown promise in personalized cancer vaccines, enabling endogenous antigen expression and potent immune activation.
(iii) Immunogenic cell death (ICD):
  - Small nanoparticles (~10 nm) loaded with doxorubicin or oxaliplatin can induce ICD in tumor cells, releasing danger-associated molecular patterns (DAMPs) and enhancing antigen presentation [221].
(iv) Immunomodulator delivery:
  - Sub-15 nm dendrimers delivering IL-2, TGF-β inhibitors, or checkpoint-blocking peptides locally within tumors modulate the TME and restore T-cell function.

Induction of Immunogenic Cell Death (ICD):

One of the notable mechanistic insights reported is that the cytotoxicity from these nanoparticles resulted in a shift in the immunogenic cell death (ICD) marker calreticulin to the cell surface in vitro and in vivo (Sargsian et al., 2024) [222]. This externalization of calreticulin is significant because it marks dying tumor cells in a way that can enhance immune recognition, thereby potentially transforming otherwise immunologically cold tumors into hot tumors that respond better to immunotherapies.

Synergy with Cancer Immunotherapy:

The study by Sargsian et al. (2024) [222] further demonstrates an in vivo application where subcutaneous Renca tumors were treated with anti-PD1 either alone or in combination with Ag-citrate–5 nm nanoparticles. The combination therapy resulted in a significant reduction in tumor size, increased necrosis, and immune cell infiltration at the tumor site. This suggests that nanoparticles in the lower nanometer range can serve as adjuvants, altering the tumor microenvironment (TME) and enhancing the efficacy of immune checkpoint blockade (ICB) therapies. Table 7 summarizes the advantages of sub-15 nm nanoparticles over larger nanoparticles in immune applications.

Smaller nanoparticles can avoid rapid clearance by phagocytes, allowing more precise delivery to lymphoid tissues and avoiding systemic immunotoxicity.

#### 3.6.4. Challenges and Considerations

While sub-15 nm nanocarriers offer multiple advantages, they also pose challenges:

- Lower antigen loading capacity due to limited volume;
- Stability issues, requiring robust surface coating (e.g., PEG, zwitterionic polymers);
- Risk of rapid renal clearance if below renal filtration threshold (~5–6 nm);
- Potential immune tolerance if poorly immunogenic antigens are presented without sufficient adjuvanticity.

Overcoming these challenges requires rational particle design, co-delivery strategies, and thorough immunological characterization.

Sub-15 nm nanoparticles represent a cutting-edge approach to vaccine and immunotherapy platforms. Their ability to target lymph nodes, efficiently deliver antigens and adjuvants, and reprogram immune responses positions them as key enablers of personalized immuno-oncology and next-generation prophylactic vaccines. As formulation technologies and immunological insights evolve, sub-15 nm nanovaccines and immunomodulators are poised to play a pivotal role in precision medicine.

### 3.7. Sub-15 nm Nanoparticles in Bioimaging and Theranostics

Sub-15 nm nanoparticles have emerged as powerful platforms in bioimaging and theranostics, offering integrated diagnostic and therapeutic capabilities at the nanoscale. Their ultrasmall size, high surface-to-volume ratio, and tunable optical, magnetic, and electronic properties make them ideally suited for precise imaging and image-guided therapy in oncology. These nanoparticles enable multimodal imaging, deep tissue penetration, and co-delivery of imaging agents with therapeutic payloads, forming the cornerstone of theranostic nanomedicine.

#### 3.7.1. Advantages of Using Sub-15 nm Nanoparticles in Bioimaging

The effectiveness of bioimaging agents depends on tissue penetration, target specificity, clearance, and signal-to-background ratio. Sub-15 nm nanoparticles uniquely satisfy these criteria through:

- Efficient tumor penetration and distribution, particularly in solid tumors with dense stroma or poor vascularization;
- Improved renal clearance, reducing background signal and systemic toxicity for diagnostic agents;
- High surface area for conjugation of targeting ligands and imaging probes (e.g., fluorophores, radioisotopes, contrast agents);
- Size-dependent quantum effects, enabling tunable emission for fluorescence imaging.

#### 3.7.2. Fluorescence and Optical Imaging

Quantum dots (QDs) are semiconductor crystals, the sizes of which are between 1 and 10 nanometers [223,224]. There is an energy distance between the valence and conduction layers, termed the band gap. Different sizes of quantum dots determine different band gaps, and different band gaps need different energies to excite the quantum dots [225]. Quantum dots have broad absorption spectra and tunable fluorescence emission. Quantum dots operate via FRET, with a large Stokes shift producing long wavelengths to increase tissue penetration depth and reduce background autofluorescence [226,227]. They are photostable with a narrow and symmetric emission band, have an excellent molar extinction coefficient that is more than 10 times larger than that of organic dyes, last relatively long to create a better signal-to-noise ratio, and, therefore, typically demonstrate a very bright light [228].

Quantum dots (QDs) and carbon-based nanoparticles (e.g., carbon dots, graphene quantum dots) are among the most widely studied < 15 nm fluorescent nanoprobes.

Key features:

- Size-tunable photoluminescence due to quantum confinement (emission ranges from UV to NIR);
- High quantum yield and photostability;
- Surface functionalization for targeting tumors, specific receptors, or organelles.

Example:

- <10 nm graphene quantum dots functionalized with folate exhibited NIR fluorescence imaging and targeted detection of ovarian tumors in vivo [95].

In 2021, J. Smith and colleagues published a study in ACS Nano exploring the use of ultrasmall quantum dots for biomedical applications, highlighting their potential in areas like bioimaging, drug delivery, and diagnostics [229].

Carbon dots and dye-doped silica nanoparticles (<10–15 nm) have also been used for cell labeling, tracking, and tumor imaging, with advantages in biocompatibility and rapid clearance.

The nanoparticles in the 5–15 nm size range—exemplified by the 6–nm core–shell silica nanoparticles discussed in the context—offer several advantages:

- They are tailored for targeting specific molecular markers, as each batch is functionalized with distinct melanoma targeting ligands.
- Their ultrasmall size offers enhanced tissue penetration and rapid clearance, reducing non–specific background.
- Their near–infrared fluorescence facilitates deep tissue imaging with high contrast.
- When combined with PET imaging, they enable precise, image–guided, and multiplexed interrogation of cancer metastases.

These characteristics support the use of such nanoparticles for advanced intraoperative imaging and targeted surgical treatment, potentially leading to improved accuracy in detecting micrometastases and overall better outcomes in cancer treatment (Chen et al., 2019) [230].

The nanoparticles exemplified by the 15 nm TiO_2_ nanoparticles used by Wang et al. (2023) [231]—demonstrate several beneficial characteristics. Their small size and high surface area contribute to exceptional optical and mechanical properties, which can be harnessed to fabricate advanced optical components like high-performance solid immersion lenses. These components, in turn, enable significant enhancements in imaging resolution while maintaining practical features such as fast processing, real-time imaging, and cost-effectiveness.

Applications in Optical Imaging

- The assembled nanoparticle (solid immersion lens) SIL is used for achieving wide-field and real-time super-resolution optical imaging. This means that devices based on such nanoparticles are capable of breaking the optical diffraction limit, enabling visualization of nano-scale details that are not normally resolvable with standard optical microscopes.
- The technology also proves versatile, being applicable to the observation of nanomaterials, cancer cells, and living cells or bacteria. This broad applicability underscores how the unique properties of nanoparticles in this size range (here exemplified by 15 nm TiO_2_) can be harnessed for diverse biological and material science applications.
- The technique is highlighted as offering a fast, wide-field, real-time, non-destructive, and low-cost solution for improving the quality of optical microscopic observation, which is a significant benefit in both research and diagnostic settings.

#### 3.7.3. Magnetic Resonance Imaging (MRI)

Ultrasmall superparamagnetic iron oxide nanoparticles (USPIONs), typically < 20 nm, are widely used as T1 or T2 MRI contrast agents. Their small size:

(a) Increases blood half-life and tumor uptake;
(b) Enhances T1-weighted contrast at low concentrations;
(c) Reduces liver/spleen accumulation compared to larger SPIONs.

Example:

- Sub-5 nm Mn-doped iron oxide nanoparticles offered dual T1/T2 imaging of brain tumors with minimal off-target retention [232].

Surface coatings such as PEG, dextran, or zwitterionic polymers improve biocompatibility and colloidal stability for in vivo imaging.

#### 3.7.4. Computed Tomography (CT) and Photoacoustic Imaging

AuNPs < 15 nm provide excellent X-ray attenuation for CT imaging and strong optical absorption for photoacoustic imaging.

Applications:

- CT contrast agents: Sub-15 nm AuNPs accumulate in tumors and lymph nodes, enabling high-resolution, real-time anatomical imaging.
- Photoacoustic imaging: Small AuNPs and semiconducting polymer dots enable deep tissue visualization with ultrasound-coupled optical contrast [233].

In one study, titanium nitride (TiN) nanoparticles were produced by using a method based on femtosecond laser ablation in liquids. This process results in ultrapure, size-tunable nanoparticles, with the study reporting even particles with dimensions below 7 nm (Popov et al., 2019) [234].

An important characteristic of these TiN nanoparticles was their strong and broad plasmonic response. The authors noted that TiN NPs demonstrate a strong and broad plasmonic peak around 640–700 nm with a significant tail over 800 nm even for small NPs sizes (<7 nm) (Popov et al., 2019) [234]. This optical feature is particularly valuable for biomedical applications since the plasmonic absorption falls within the so-called relative tissue transparency window. In this wavelength region, light penetration in tissues is enhanced, making these nanoparticles promising for techniques such as absorption/scattering contrast imaging, photoacoustic imaging, and especially photothermal therapy.

In summary, the comprehensive study on plasmonic TiN nanoparticles synthesized with femtosecond laser ablation demonstrates that:

- The synthesis method can produce ultrapure, size-tunable nanoparticles, with reported sizes even below 7 nm, fitting well within the 5–15 nm range.
- These nanoparticles exhibit a strong and broad plasmonic peak in the 640–700 nm region, with an extended tail over 800 nm, favoring their use in biomedical applications that require tissue-penetrating optical properties.
- Biological testing revealed that these nanoparticles exhibit low cytotoxicity and excellent cell uptake.
- They have been successfully used in photothermal therapy, signifying their potential to advance modalities such as contrast imaging, photoacoustic imaging, and surface-enhanced Raman scattering (SERS).

#### 3.7.5. Nuclear Imaging (PET/SPECT)

Sub-15 nm nanoparticles functionalized with radiometals (e.g., ^64^Cu, ^99m^Tc,^ 68^Ga) allow sensitive and quantitative PET or SPECT imaging.

Advantages:

- Rapid clearance reduces radiation dose.
- Small size enables receptor-mediated targeting.
- Useful for whole-body imaging of cancer metastasis and therapeutic response

Example:

- ^64^Cu-labeled dendrimers (~5–8 nm) conjugated with RGD peptides demonstrated PET imaging of integrin-expressing tumors with an excellent signal-to-noise ratio [235].

#### 3.7.6. Therapeutic Applications: Integrated Diagnosis and Therapy

Theranostics refers to nanocarriers capable of simultaneous imaging and therapy. Sub-15 nm nanoparticles are ideal for this due to:

- Co-delivery of therapeutic agents (e.g., drugs, siRNA) and imaging labels.
- Real-time monitoring of biodistribution, tumor accumulation, and therapeutic response.
- Stimuli-responsive release triggered by pH, redox, enzymes, or light.

Theranostic examples:

- Gold nanoshells (~15 nm) for photoacoustic imaging and NIR-triggered photothermal therapy.
- GQDs delivering doxorubicin and enabling fluorescence imaging-guided chemotherapy.
- Iron oxide nanoparticles releasing immune checkpoint inhibitors with MRI tracking.

Such systems are advancing toward personalized medicine, where treatment regimens are adjusted based on real-time imaging feedback.

Sub-15 nm nanoparticles have significantly advanced the field of bioimaging and theranostics, offering integrated platforms for cancer diagnosis, monitoring, and therapy. Their favorable pharmacokinetics, enhanced tumor penetration, and multifunctionality enable precise, minimally invasive strategies for managing cancer and other diseases. Ongoing innovations in surface engineering.

## 4. Engineering Sub-15 nm Nanoparticles

Engineering nanoparticles in the sub-15 nm range requires meticulous control over material selection, synthesis techniques, and surface functionalization to achieve desired physicochemical properties while maintaining stability and biocompatibility.

### 4.1. Material Selection

The choice of material is a critical determinant of the size, morphology, and functionality of sub-15 nm nanoparticles. Common classes include:

- Polymeric systems: Amphiphilic block copolymers (e.g., PEG-PLA, PEG-PLGA) and dendrimers such as PAMAM and PPI can form sub-15 nm micellar structures via self-assembly [236,237]. These systems provide flexibility in tuning hydrophobic core sizes and surface hydrophilicity.
- Lipid-based systems: Lipid micelles and small unilamellar liposomes (≤15 nm) have been engineered using high-pressure extrusion or microfluidics [238].
- Inorganic nanoparticles: Quantum dots, gold nanoclusters, and ultrasmall silica nanoparticles are often synthesized below 15 nm and are widely studied for imaging and drug delivery [239,240].
- Biomolecular nanostructures: Protein-based and DNA-origami-based nanoparticles are emerging tools for engineering precise and uniform structures in the sub-15 nm range [241].

### 4.2. Synthesis and Size Control Techniques

Achieving uniform particle size below 15 nm demands highly controlled synthesis methodologies:

- Self-assembly: For polymeric micelles and dendrimers, critical micelle concentration (CMC), solvent polarity, temperature, and molecular weight ratios are optimized to yield sub-15 nm sizes [242].
- Microfluidics: Continuous-flow microfluidic systems allow fine control over mixing and nucleation kinetics, producing monodisperse sub-15 nm particles with high reproducibility [243].
- Reverse microemulsion: This technique enables synthesis of ultrasmall inorganic nanoparticles by confining nucleation and growth within nanoscopic water droplets in an oil phase [244].
- Ultrasonication and extrusion: Lipid-based systems are downsized using high-energy mechanical processes, often combined with surfactants to maintain structural integrity at nanoscale dimensions [238].

### 4.3. Surface Functionalization and Stabilization

Surface engineering is pivotal in preserving colloidal stability, enhancing circulation time, and enabling active targeting:

- PEGylation: Polyethylene glycol (PEG) chains confer steric stabilization and reduce protein adsorption, minimizing RES uptake [245]. However, dense PEG coronas may hinder cellular uptake; thus, length and density must be optimized [246].
- Zwitterionic and hydrophilic coatings: Zwitterionic polymers and hydrophilic moieties like polysaccharides offer alternatives to PEG for stealth behavior [247].
- Ligand conjugation: Antibodies, peptides, aptamers, and small molecules (e.g., folic acid, RGD peptides) can be attached to the nanoparticle surface for receptor-mediated targeting [248].
- Charge tuning: Slightly negative or near-neutral surface charges are generally favored to reduce nonspecific interactions with serum proteins and avoid rapid clearance [249].

### 4.4. Encapsulation and Drug Loading Strategies

Drug loading in sub-15 nm systems must balance loading efficiency with particle integrity:

- Core-loading: Hydrophobic drugs can be solubilized in the core of micelles or lipid-based carriers. However, the core volume is limited in sub-15 nm particles, often leading to lower drug loading [250].
- Surface adsorption or conjugation: Small molecule drugs or nucleic acids can be electrostatically bound or covalently conjugated to nanoparticle surfaces using cleavable linkers (e.g., pH-sensitive, redox-responsive) [251].
- Matrix entrapment: Inorganic particles may encapsulate drugs within porous frameworks or through coordination chemistry, especially in metal–organic hybrids or mesoporous silica [252].

### 4.5. Considerations for Scalability and Reproducibility

For clinical translation, engineering methods must ensure reproducibility, scalability, and regulatory compliance:

- Process scalability: Techniques like microfluidics, flash nanoprecipitation, and high-shear homogenization are being optimized for GMP-compatible production [253].
- Batch consistency: Real-time monitoring tools such as dynamic DLS, TEM, and field-flow fractionation (FFF) are employed to ensure narrow size distributions and reproducibility [254].
- Stability optimization: Lyophilization with cryoprotectants or formulation in aqueous buffers with stabilizers (e.g., trehalose, surfactants) is often necessary for long-term storage [255].

#### Sub-15 nm Nanoparticles in Industry

Quantum dot nanoparticles of the NNCrystal US Corporation [256], a subsidiary of Hangzhou Najing Technology, are a pioneer in the production and processing of high-quality semiconductor nanocrystals of sub-15 nm particles. They are an innovator focused on applications of these novel materials in imaging. Thermo Fisher Scientific offers ultrasmall quantum dots (uQDs) for high-sensitivity imaging applications [257], emphasizing enhanced photostability and reduced toxicity compared to larger quantum dots.

Ocean NanoTech’s [258] PEG Iron Oxide Nanoparticles are superparamagnetic particles with excellent colloidal stability and biocompatible coating for biomedical applications, including in vivo magnetic resonance imaging (MRI), magnetic particle imaging (MPI), magnetic sensing for in vitro diagnostics, small molecular drug delivery, immunotherapy, hyperthermia, adjuvant for vaccine, etc. PEG magnetic nanoparticles are nanosized (10–30 nm) iron oxide particles with polyethylene glycol groups. With excellent colloidal stability and unique surface coating, the PEG magnetic nanoparticles exhibit low non-specific binding of protein or nucleic acids.

Nanosys’^®^ [259] proprietary quantum dot and microLED technology offer the closest thing to a truly lifelike color experience. Quantum dots produce pure color, without the waste. Its quantum dots create a perfectly tuned spectrum of light with exactly the right red, green, and blue that the display needs for accurate, lifelike color reproduction with high brightness.

Nanoco [260] specializes in creating bespoke next-generation nanomaterials tailored to specific applications. Nanoco is uniquely placed to be able to scale up and manufacture at the high volumes required for consumer electronics applications.

**Figure 4:**
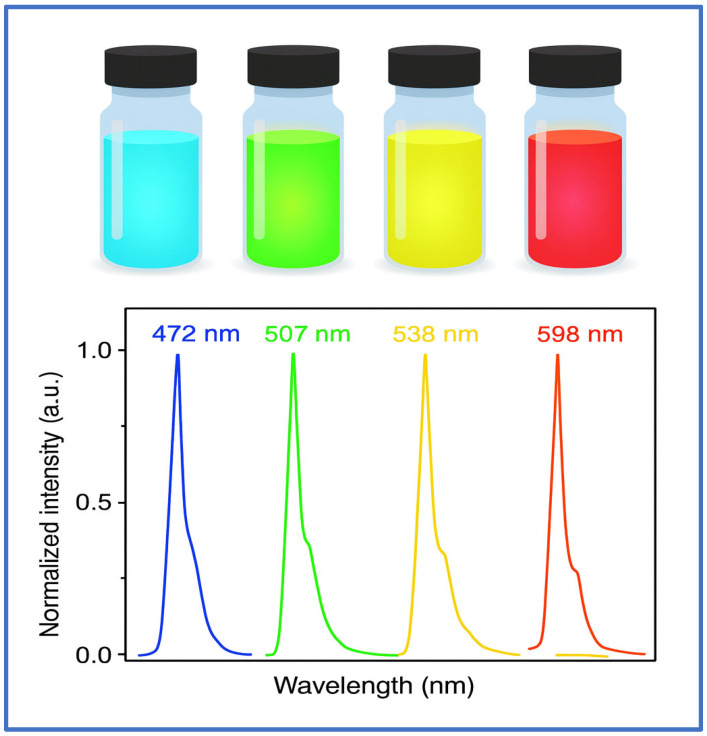
Photographs of fluorescence images under UV light and spectra of Carbon Dots (C-dots). Adapted from Fanglong Yuan et al., 2018 [263].

BOC Sciences offers a wide range of quantum dots products as well as customized products in different types to meet a variety of research and application needs [261]. CD Bioparticles provide various C-dots with uniform size and different functional groups on the surface. Our C-dots are generally less than 10 nm with different solubility and emission wavelengths ranging from 302 nm to 705 nm [262]. The carbon dots fluorescence images under UV light and spectra are shown in Figure 4 below.

## 5. Regulatory and Quality Considerations

The development and clinical translation of sub-15 nm nanoparticles raise unique regulatory and quality challenges due to their small size, complex behaviors in biological systems, and lack of standardized guidelines specific to the ultrasmall nanomaterial category. While general regulatory frameworks for nanomedicines exist, sub-15 nm nanoparticles often exhibit distinct pharmacokinetics, biodistribution, and toxicity profiles, requiring tailored considerations during product development and evaluation.

### 5.1. Regulatory Classification and Product Definition

Nanoparticles below 15 nm can fall under various regulatory categories depending on their composition and intended use—such as drug, biologic, device, or combination product. The FDA’s Nanotechnology Task Force recognizes the size range as a “continuum,” acknowledging that even particles as small as 1–10 nm may exhibit unique behavior due to increased surface area, altered biological interactions, and quantum effects [264]. Regulatory agencies such as the U.S. Food and Drug Administration (FDA), the European Medicines Agency (EMA), and other global bodies are progressively adapting guidelines to accommodate the distinctive nature of nanomedicines, though no specific pathway currently exists solely for sub-15 nm platforms [265,266]. The absence of a standardized regulatory definition for “nanomedicine” necessitates a case-by-case review, with emphasis on material characteristics, manufacturing controls, and clinical performance [267].

Given that nanomedicines are reviewed case-by-case and no single pathway exists specifically for sub-15 nm, we propose submitting an orthogonal characterization plan upfront (anchored to the CQAs below) and explicitly justifying acceptance criteria for the ultrasmall range.

### 5.2. Quality by Design (QbD) and Critical Quality Attributes (CQAs)

A Quality by Design (QbD) approach is pivotal for the development of sub-15 nm nanoparticles. Given the scale-dependent functionality of these systems, size distribution, surface charge (zeta potential), polydispersity index (PDI), drug loading efficiency, and surface ligand density must be carefully defined as Critical Quality Attributes (CQAs) [264,268]. Sub-15 nm NPs are especially sensitive to small changes in formulation or process parameters, necessitating stringent control strategies [269].

### 5.3. Analytical Challenges and Method Standardization

The characterization of sub-15 nm nanoparticles poses analytical challenges due to their small dimensions and dynamic behavior in biological matrices. Techniques such as DLS, TEM, nanoparticle tracking analysis (NTA), and asymmetrical flow field-flow fractionation (AF4) are commonly used [270,271], but each has limitations in resolution, sample compatibility, or quantification accuracy. Regulatory acceptance often requires orthogonal methods and robust method validation [264].

Furthermore, stability testing of ultrasmall nanoparticles requires specific consideration of agglomeration, degradation of functional ligands, and chemical transformations over time [272]. Stability-indicating methods should be designed to detect subtle physicochemical changes that may impact safety or efficacy.

Because sub-15 nm systems operate near the resolution limits of many techniques, orthogonal analytics are essential. We define a minimal panel (e.g., DLS for hydrodynamic size/PDI; TEM/cryo-TEM for morphology; NTA/AF4 for number-based distributions; method-specific ligand-density assays) and treat size distribution, surface charge, PDI, loading efficiency, and ligand density as CQAs under our QbD plan.

### 5.4. Toxicological Considerations and Non-Clinical Evaluation

Due to their ultrasmall size, sub-15 nm nanoparticles may:

- Cross biological barriers more readily (e.g., blood–brain barrier, placental barrier);
- Evade immune surveillance or, conversely, provoke unexpected immune responses;
- Exhibit nonlinear dose–response relationships.

Thus, traditional toxicology models may not fully predict human outcomes. Regulatory bodies emphasize the need for:

- Tailored nonclinical safety assessments, including immunotoxicity, genotoxicity, and reproductive toxicity;
- Evaluation of accumulation and clearance in organs such as the kidney, liver, and spleen;
- Long-term studies for chronic exposure when applicable.

Ultrasmall carriers can display distinct PK, biodistribution, and toxicity versus larger nanoparticles—e.g., faster tissue diffusion, different organ exposure, and size-sensitive clearance. We therefore emphasize case-by-case evaluation and specify dose metrics (mass and particle number) appropriate for this regime.

### 5.5. Sterility, Endotoxins, and Impurities

For parenteral formulations, sterility and endotoxin control are of paramount importance. The high surface-area-to-volume ratio of sub-15 nm NPs increases the risk of adsorbing pyrogens or leachable impurities from manufacturing equipment and excipients. Endotoxin detection using Limulus Amebocyte Lysate (LAL) assays may be interfered with by the particles themselves, necessitating validated mitigation strategies [273].

Impurity profiling—both process-related (e.g., residual solvents, unreacted monomers) and degradation-related—is critical, particularly since trace impurities may have disproportionate biological effects due to the nanoparticle’s ability to interact at the cellular or molecular level [11].

### 5.6. Regulatory Submissions and Clinical Translation

For investigational new drug (IND) applications or clinical trial approvals involving sub-15 nm nanoparticles, a comprehensive Chemistry, Manufacturing, and Controls (CMC) dossier is essential. This should include detailed information on:

- Raw material characterization and specifications.
- Manufacturing process flow and in-process controls.
- Validation of analytical methods.
- Stability data under ICH conditions.
- Non-clinical safety data relevant to nanoparticle-specific behavior.

The FDA encourages early scientific advice and pre-IND meetings to align on expectations [265,274], especially when novel nanoparticle technologies are involved. Similarly, the EMA’s Innovation Task Force (ITF) offers early dialog for advanced therapies, including nanomedicines [266].

## 6. The Future Prospects of Sub-15 nm Nanoparticles

Sub-15 nm nanoparticles in biological applications are poised to be transformative, driven by advancements in materials science, synthetic methodologies, and systems biology. The understanding of the nanoscale interface between engineered materials and biological systems is deepening and several future directions emerging for exploiting sub-15 nm nanoparticles in a more precise and translational manner.

### 6.1. Smart Nanoparticles and Precision Engineering

Future research will focus on the rational design of sub-15 nm nanoparticles with enhanced control over physicochemical properties such as shape, surface chemistry, and dynamic behavior in biological environments. Advancements in molecular self-assembly, atomically precise clusters, and ligand exchange chemistry will allow for the fabrication of “smart” nanoparticles that can respond to biological stimuli—such as pH, redox conditions, or enzyme concentrations—to trigger drug release or switch biological activity [275,276]. Incorporation of biosensors and responsive elements could transform these ultrasmall systems into diagnostic–therapeutic hybrid agents (theranostics).

### 6.2. Crossing Biological Barriers and Enhancing Penetration

One of the major advantages of sub-15 nm nanoparticles is their ability to penetrate dense biological barriers such as the tumor extracellular matrix, mucosal layers, and even intracellular compartments like the nucleus. Future efforts are likely to emphasize strategies that further exploit these features, potentially by tuning surface softness, shape flexibility, or leveraging cell-penetrating peptides and nuclear localization signals [9,277,278]. This may open avenues for efficient intracellular gene editing, antisense therapies, and targeted chemotherapy.

### 6.3. Clinical Translation and Regulatory Science

Despite promising preclinical outcomes, clinical translation of sub-15 nm nanoparticles remains limited. Future perspectives should consider not only the design of safe and effective nanoparticles but also the establishment of standardized regulatory frameworks and evaluation metrics tailored to this unique size class. The development of Good Manufacturing Practice (GMP)-compliant synthesis protocols, validated analytical characterization tools, and robust pharmacokinetic models is essential for clinical adoption [269,279].

#### Clinical Landscape of Sub-15 nm Nanoparticles

Clinically advanced examples for sub-15 nm nanoparticles remain concentrated in three families: ultrasmall silica platforms (C’ dots), gadolinium–polysiloxane theranostics (AGuIX), and gold-core spherical nucleic acids (SNAs)—with additional clinical imaging activity in reconstituted high-density lipoprotein (HDL) mimetics (≈9–12 nm). In general, they show a pattern of deep tumor penetration, renal-compatible clearance, and multimodal imaging/RT synergy that aligns with the size-dependent arguments developed earlier in the review. Representative clinical programs of sub-15 nm nanoparticles are shown below in Table 8.

First-in-human ^124^I-cRGDY-PEG–Cʹ dots demonstrated tolerability, renal clearance, and molecularly targeted accumulation in melanoma patients (optical/PET). This program established a clinical precedent for renally excreted, sub-10 nm inorganic nanoparticles [280].

NU-0129 (Au-core SNA carrying siRNA against BCL2L12) completed a first-in-human Phase 0 study in recurrent GBM, demonstrating intratumoral delivery and target engagement after IV dosing prior to resection (NCT03020017). Note the Au core is ~13 nm; the hydrodynamic diameter can exceed 15 nm depending on the nucleic-acid corona, but the core sits in the sub-15 nm regime. SNAs illustrate a size-enabled nucleic-acid transport architecture with early human evidence of brain delivery—positioned at the boundary of this review’s size window once the oligonucleotide shell is considered [281].

### 6.4. Biodegradable and Clearance Optimized Nanomaterials

A growing trend is the shift toward biodegradable sub-15 nm nanoparticles composed of materials such as ultrasmall polymeric micelles, peptides, and metal–organic clusters that can be excreted via renal or hepatobiliary pathways without long-term tissue accumulation [8,283,284]. This strategy mitigates concerns related to chronic toxicity and enhances clinical acceptance. Future work should focus on the creation of bioresponsive degradation mechanisms and excretion profiles suitable for repeated dosing.

### 6.5. Personalized Nanomedicine and Immune Modulation

Emerging studies suggest that sub-15 nm nanoparticles may interact with the immune system in unique ways, potentially enabling personalized immune modulation. Tailoring surface ligands, protein corona profiles, or cargo release in response to patient-specific immune status could revolutionize cancer immunotherapy and vaccine delivery [285,286]. Systems biology approaches integrating patient omics data with nanoparticle design will play a crucial role in this vision.

## 7. Conclusions

Sub-15 nm nanoparticles represent a significant milestone in the evolution of nanomedicine and drug delivery systems. These ultrasmall nanocarriers exhibit distinct physicochemical and biological behaviors compared to their larger counterparts, primarily due to their high surface-area-to-volume ratios, unique biodistribution profiles, and improved tissue penetration capabilities [236,242,249]. Their ability to navigate biological barriers more effectively, including enhanced tumor penetration and renal clearance, has made them particularly attractive for cancer therapy, imaging, and precision medicine applications [269].

Despite these promising attributes, the successful design and clinical translation of sub-15 nm nanoparticles present considerable challenges. Engineering such nanoparticles requires precise control over material selection, synthesis conditions, and surface functionalization to ensure biocompatibility, stability, and reproducibility [240,243,244]. Polymeric micelles, dendrimers, lipid-based carriers, and inorganic nanostructures have been successfully tailored into the sub-20 nm domain, with various encapsulation strategies allowing for the delivery of both hydrophobic and hydrophilic drugs [237,238,239,241].

Surface engineering remains a cornerstone in optimizing pharmacokinetics and minimizing immune recognition. Strategies such as PEGylation, zwitterionic coatings, and targeted ligand conjugation have shown efficacy in prolonging circulation time and enhancing tumor targeting, though their performance in the sub-15 nm size regime continues to be an area of active investigation [246,247,248]. The role of opsonization, blood clearance, and protein corona formation becomes even more critical at these small sizes [245,249].

From a translational perspective, significant work is still required to overcome hurdles associated with large-scale manufacturing, long-term stability, and regulatory approval. The limited drug loading capacity due to the reduced core volume and the potential for rapid renal elimination must also be addressed through innovative formulation approaches and adaptive design [250,287]. Smart nanocarriers that respond to stimuli (e.g., pH, temperature, enzymes) offer potential solutions in this regard [251].

Looking forward, the integration of advanced fabrication techniques (e.g., microfluidics, self-assembly), real-time analytical characterization, and artificial intelligence-driven design holds promise for optimizing sub-15 nm nanoparticle platforms [252,253,288]. Additionally, expanding the understanding of protein corona dynamics, immune system interactions, and long-term toxicity profiles will be critical for their widespread clinical acceptance [254,289,290]. Stabilization techniques such as lyophilization using appropriate cryo-/lyo-protectants also require further refinement to ensure formulation robustness [255].

To accelerate sub-15 nm nanomedicine from concept to clinic, the following priorities were identified:

(a) There is a need to establish quantitative design rules for ultrasmall formats (size–corona–charge windows that maximize target engagement) while preserving stability/clearance using standardized in vitro–in vivo correlation (IVIVC) panels and barrier models relevant to tumors and CNS.
(b) Barrier-aware engineering systematically tests how softness, aspect ratio, and ligand topology govern penetration across the ECM, mucosa, and nuclear envelopes; we need to couple these studies with responsive (smart) chemistries that activate cargo release only after barrier transit.
(c) There is also a need to build assay batteries for the toxicology of sub-15 nm nanoparticles that capture ultrasmall-specific hazards (immune modulation, nonlinear dose–response, off-target barrier crossing) and agree on minimal datasets for repeat-dose studies and long-term clearance.
(d) Lastly, we need to develop metrology and reference materials for validated methods (size, polydispersity, protein corona, ligand density) and community reference particles in the 5–15 nm regime to ensure cross-lab reproducibility and support regulatory review.

In summary, sub-15 nm nanoparticles stand at the frontier of nanomedicine, offering transformative potential in targeted drug delivery and diagnostics. Their future success hinges on continued interdisciplinary collaboration between materials scientists, pharmaceutical researchers, clinicians, and regulatory bodies to bridge the gap between bench and bedside.

## Figures and Tables

**Table 1:** Different types of PNPs based on structure: (a) nanocapsules, (b) nanospheres, (c) micelles, (d) dendrimers, (e) polymersomes, and (f) polyplexes.

Images	Type	Typical Size Range (nm)	Development Status	Unique Properties	Example Drugs/Applications	Key References
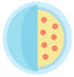	Nanocapsules (a)	5–20 (can be <15)	Preclinical, Some clinical	Reservoir structure with drug in core and polymer shell; tunable release kinetics	Curcumin, Dexamethasone, Insulin	[2,3]
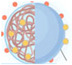	Nanospheres (b)	10–50 (some <15 possible)	Preclinical	Matrix system where drug is uniformly dispersed in polymer matrix	Paclitaxel, 5-FU, Doxorubicin	[4,5]
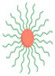	Micelles (c)	5–15	Clinical and preclinical	Amphiphilic self-assembly; core–shell; good for hydrophobic drugs; often PEGylated	Paclitaxel (Genexol^®^-PM), Doxorubicin, siRNA	[6,7,8]
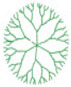	Dendrimers (d)	1–10	Clinical (few), preclinical	Hyperbranched, monodisperse, multivalent surface; ideal for targeted drug delivery	Cisplatin, siRNA, Methotrexate, NSAIDs	[9,10]
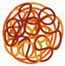	Polymersomes (e)	10–50 (rarely <15)	Emerging/preclinical	Bilayer vesicles; stable; carry both hydrophilic and hydrophobic drugs	Proteins, siRNA, Anticancer agents	[11,12]
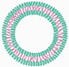	Polyplexes (f)	5–15	Preclinical, some clinical	Complex of cationic polymer with DNA/RNA; gene therapy focus	DNA/RNA vaccines, CRISPR-Cas9 delivery	[13,14]

**Table 2:** Comparative Considerations of Lipid-Based Nanoparticles.

Parameter	LNPs	Liposomes	Nanoemulsions	SLNs
Structure	Amorphous lipid core	Lipid bilayer	Oil droplets	Solid lipid matrix
Drug types	RNA, small molecules	Hydrophilic and hydrophobic	Hydrophobic	Mostly hydrophobic
Typical size (<15 nm achievable or not)	Yes	Yes	Yes	Yes (rarely)
Stability	Moderate to high	Moderate	High (kinetic)	High (physical)
Challenges	PEG-shedding, stability	Low loading, rapid clearance	Surfactant toxicity	Low drug loading at ultrasmall sizes

**Table 3:** Comparison Between Metallic Nanoparticles.

Property	Gold NPs	Silver NPs	Iron Oxide NPs
Typical size range (sub-15 nm)	1–15 nm	2–15 nm	5–15 nm
Key applications	Photothermal, drug delivery, imaging	Cytotoxicity, antimicrobial, chemo	MRI, hyperthermia, CDT
Surface modification	Thiols, PEG, ligands	Polymers, bioreduction agents	Dextran, PEG, citrate
Advantages	Biocompatible, stable	Potent cytotoxicity	Magnetic targeting, imaging
Limitations	Expensive, limited biodegradation	Potential toxicity	Agglomeration, synthesis complexity

**Table 4:** Comparative characteristics of sub-15 nm and 15–30 nm nanoparticles for drug delivery.

Parameter	Sub-15 nm Nanoparticles	15–30 nm Nanoparticles	Implications/Trade-Off
Typical size regime	5–15 nm (often near 10 nm)	15–30 nm (EPR-optimized range)	Defines distinct physicochemical and biological behaviors
Tumor/tissue penetration	Excellent diffusion and deep stromal penetration; cross BBB and dense ECM	Moderate; relies mainly on EPR effect and leaky vasculature	Smaller systems favored for desmoplastic or poorly vascularized tumors
Drug/payload capacity	Limited core volume; lower loading; suited for potent small molecules or conjugates	Larger internal volume; higher encapsulation of macromolecules (siRNA, mRNA, proteins)	Larger systems preferred when payload size dominates
Colloidal and storage stability	Higher surface energy; prone to aggregation; requires PEGylation, zwitterionic, or lyophilized stabilization	Generally stable under physiological ionic strength; less surface-energy stress	Sub-15 nm systems need careful formulation control
Circulation and clearance	Rapid renal elimination (<5.5 nm full clearance); minimal RES uptake; short systemic half-life	Slower renal filtration; longer circulation; possible hepatic/splenic accumulation	Balance between safety (fast clearance) and retention (long exposure)
Biodistribution pattern	Diffuse distribution; low organ retention; favorable for repeat dosing	Prolonged tumor residence; potential off-target organ accumulation	Depends on therapy duration and dosing frequency
Biological interactions	High surface-area-to-volume ratio; quantum and charge-dependent behavior	Classical colloidal interactions; surface-driven uptake via endocytosis	Unique quantum/charge effects emerge only below ~15 nm
Formulation complexity	Demanding size control; requires advanced synthetic precision (SCNPs, dendrimers, ultrafine emulsions)	Easier scale-up using standard micelle/liposome/nanoemulsion techniques	Manufacturability favors mid-size range
Therapeutic applications	Imaging agents, photothermal/photodynamic nanodots, small-molecule chemotherapeutics, renal-clearable probes	High-payload formulations for nucleic acids, proteins, vaccines, long-acting depots	Use sub-15 nm when diffusion and clearance dominate; 15–30 nm when payload and persistence dominate
Regulatory/translational outlook	Emerging class; limited guidelines; requires new PK/Tox paradigms	Better-defined; more precedent from approved nanomedicines	Sub-15 nm systems are frontier candidates for next-generation precision nanomedicine

**Table 5:** Comparative Insight and Outlook Between GQDs and CNTs.

Feature	Graphene Quantum Dots (GQDs)	Carbon Nanotubes (CNTs)
Typical size	<10 nm	Diameter: 0.8–20 nm
Primary strengths	Bright/stable PL; metal-free; easy bioconjugation; PDT	Very high loading; strong PTT; PA imaging
Best-fit applications	Fluorescence imaging, PDT, light-guided chemo	PTT, gene/drug delivery at high dose density, PA/sensing
Drug loading (qualitative)	Moderate (π-π, H-bonding, conjugation)	High (adsorption, covalent, endohedral)
Renal clearance potential	Higher (≤10 nm, sheet-like)	Lower (needs oxidation for clearance)
Inflammation/bioperpersistence risk	Low-moderate	Moderate-high unless heavily functionalized
Typical functionalization	Carboxyl/amine/PEG/targeting ligands	Oxidation/PEGylation/amide/ester; targeting ligands
Safety focus	Oxidative stress at high dose	Lung/RES persistence

**Table 6:** Comparison of Synthesis Methods of Particles Less than 15 nm.

Intended Use	Preferential Platforms	Why Synthesis Fit	Watch-Outs for Production
Deep tumor imaging/PDT/PTT	GQDs, QDs (Cd-free), Au (<15 nm)	High PL or photothermal efficiency; straightforward top-down/bottom-up or Brust/seed routes	PL drop on aqueous transfer; aggregation in salts (add stealth)
Ligand-dense drug conjugates	Dendrimers	Precise size (G3–G5), controlled multivalency	Iterative steps, ligand-density QC, avoid inter-particle crosslinks
Solubilizing hydrophobes; fast penetration	Micelles; nanoemulsions (≤15 nm)	Self-assembly/emulsification is scalable; small hydrodynamic size	Dilution stability, Ostwald ripening; surfactant biocompatibility
Membrane protein display/receptor uptake	NLPs/HDL mimetics (~10 nm)	Belt-stabilized disks via cholate dialysis/microfluidics	Protein quality; batch reproducibility; stoichiometry control
PTT/hybrid ROS	CNTs (ultrashort) ± Ag/Au	CVD → shortening; metal decoration for ROS/SERS	Catalyst removal; biopersistence; robust dispersion/stealth needed

**Table 7:** Advantages of Sub-15 nm Nanoparticles Over Larger Nanoparticles in Immune Applications.

Parameter	Sub-15 nm Nanoparticles	Larger Nanoparticles (>50 nm)
Lymph node access	Efficient via lymphatic drainage	Limited, often require active transport
Cellular uptake by APCs	Rapid and efficient	Slower, may remain extracellular
Tumor penetration	High	Limited to perivascular regions
Immune activation	Favorable for T cell priming	May lead to immune evasion or sequestration

**Table 8:** Representative Clinical Programs of Sub-15 nm Nanoparticles.

P (Size)	Modality	Indication(s)	Stage/ID	Key Human Findings
C’ dots (silica ~5–7 nm)	Optical-PET imaging	Melanoma, SLN mapping	First-in-human (2014)	Well tolerated; renal clearance; targeted tumor uptake [280].
ELU001 (CDC) (~6–7 nm)	Drug-conjugate (exatecan), FRα-targeted	FRα+ solid tumors	Phase 1/2, NCT05001282	Early safety reported; clinical activity under evaluation.
AGuIX (~3 nm)	MRI contrast + radiosensitizer	Brain metastases, GBM	Phase II, e.g., NCT04899908; Fast Track (2024)	MRI-visible tumor uptake; randomized studies in progress.
NU-0129 (SNA) (Au core ~13 nm; HD may be > 15 nm)	siRNA gene regulation	Recurrent GBM	Phase 0, NCT03020017	Intratumoral delivery/target engagement after IV dosing [281].
rHDL/CER-001 (~9–12 nm)	Imaging/CV therapy; exploratory oncology imaging	Atherosclerosis; pilot oncology imaging	Multiple clinical studies (CV); pilot ^89^Zr-HDL PET in cancer	Human-scale safety; size compatible with tumor imaging/drug delivery concepts [282].

## Data Availability

No new data were created or analyzed in this study. Data sharing is not applicable to this article.

## Supplementary Materials

The following supporting information can be downloaded at: https://www.mdpi.com/article/10.3390/ijms262210842/s1. References [291,292,293,294,295,296,297,298,299,300,301,302,303,304,305,306,307,308,309,310,311,312,313,314,315,316,317,318,319,320,321,322,323,324,325,326,327,328,329,330,331,332,333,334,335,336,337,338,339,340,341,342,343,344,345,346,347,348,349,350,351,352,353,354,355,356,357,358,359,360,361,362,363,364,365,366,367,368,369,370,371,372,373,374,375,376,377,378,379,380,381,382,383,384,385,386,387,388,389,390,391,392,393,394,395,396,397,398,399,400,401,402,403,404,405,406,407,408,409,410,411,412,413,414,415,416,417,418,419,420,421,422,423,424,425,426,427,428,429,430,431,432,433,434,435,436,437,438,439,440,441,442,443,444,445,446,447,448,449,450,451,452,453,454,455,456,457,458,459,460,461,462,463,464,465,466,467,468,469,470,471,472,473,474,475,476,477,478,479,480,481,482,483,484,485,486,487,488,489,490,491,492,493,494,495,496,497,498,499,500,501,502,503,504,505,506,507,508,509,510,511,512,513,514,515,516,517,518,519,520,521,522,523,524,525,526,527,528,529,530,531,532,533,534,535,536,537,538,539,540,541,542,543,544,545,546,547,548,549,550,551,552,553,554,555,556,557,558,559,560,561,562,563,564,565,566,567,568,569,570,571,572,573,574,575,576,577,578,579,580,581,582,583,584,585,586,587,588,589,590,591,592,593,594,595,596,597,598,599,600,601,602,603,604,605,606,607,608,609,610,611,612,613,614,615] are cited in Supplementary Materials.

## Abbreviations

AF4/FFF	Asymmetric flow field-flow fractionation/field-flow fractionation
AuNP/AgNP	Gold/silver nanoparticle
BBB	Blood–brain barrier
CAGR	Compound annual growth rate
CMC	Critical micellar concentration
CNT	Carbon nanotube
COVID-19	Coronavirus disease 2019
CQA	Critical quality attributes
DHLA	Dihydrolipoic acid
DLS	Dynamic light scattering
CVD	Chemical vapor deposition
DOX	Doxorubicin
EPR	Enhanced permeability and retention
GBM	Glioblastoma
GQD	Graphene quantum dot
LNP	Lipid nanoparticle
LDL	Low-density lipoprotein
LSPR	Localized surface plasmon resonance
MOF	Metal–organic frameworks
MUA	11-mercaptoundecanoic acid
MWCNT	Multi-walled carbon nanotube
NIR	Near infra-red
NLP	Nanolipoprotein particle
NTA	Nanoparticle tracking analysis
PAMAM	Poly(amidoamine)
PEG	Polyethylene glycol
PD-L1	Programmed death-ligand 1
PTX	Paclitaxel
RGD	Arg-Gly-Asp peptide
SWCNT	Single-walled carbon nanotube
^5^TT	Quintet multiexciton state
TEM/SEM	Transmission/scanning electron microscopy
PTT/PDT	Photothermal/photodynamic therapy
SLN	Solid lipid nanoparticle
SPR	Surface plasmon resonance
SPION	superparamagnetic iron oxide nanoparticle
