# Supplementary material for: Sub-15 nm Nanoparticles for Drug Delivery: Emerging Frontiers and Therapeutic Potential
Source: Int J Mol Sci. 2025 Nov 8;26(22):10842. doi: 10.3390/ijms262210842 (PMC12652079; doi:10.3390/ijms262210842)
Supplement: Supplementary file 1 [file ijms-26-10842-s001.zip › ijms-3909287-supplementary.pdf]

International Journal of
Molecular Sciences

MDPI

This supplementary file provides a comprehensive tabulated data supporting the main manuscript entitled: "Sub-15 nm Nanoparticles for Drug Delivery: Emerging Frontiers and Therapeutic Potential" and presents Appendices tables S1-S3 listing nanoparticles in the 5-15 nm range investigated as pharmaceuticals, diagnostics, and theranostics. Each table summarizes their size range, development status, unique properties, applications, and literature references [291-615], serving as an extended data resource for readers.

Table S1: List of sub-15 nm ultrasmall nanoparticles investigated as pharmaceuticals

Typical Size Range	Developme Status	Unique Properties	Example Drugs Applications	References
5–10 nm (Germanium NPs)	Preclinical	Quantum confinement, red shifted PL, photothermal potential	Cancer theranostics	Fronya et al., 2022 [291]
8 nm TEM / 15 nm (CuS@BSA)	Preclinical	NIR LSPR, photothermal therapy	HeLa cancer PTT	Wan et al., 2019 [154]
10 nm (DOX-LGs)	Preclinical	High uptake, tumor accumulation, enhanced cytotoxicity	Doxorubicin micelles	Li et al., 2020 [155]
5.6–11 nm (AuNPs)	Preclinical	FA-targeted, high cytotoxicity in FR+ cells	Methotrexate	Yücel et al., 2020 [156]
15 ± 3 nm (AgNPs)	Preclinical	βCD-Mel stabilized, enhanced drug permeability	Melphalan delivery	Sierpe et al., 2023 [168]
11 ± 2 nm (ZnO-NPs)	Preclinical	Synergy with essential oil, biodegradable composites	Tissue engineering	Grande-Tovar et al., 2022 [292]
~9–15 nm subset of 9–41 nm (AuNPs)	Preclinical	Seed-coat phytosynthesis, anticancer	HepG2 cancer	Ashokkumar et al., 2014 [293]
12 ± 2 nm (AgNPs)	Preclinical	Green synthesis, anti-angiogenic	Anti-cancer Angiogenesis inhibition	Baharara et al., 2014 [294]
7–15 nm subset (AgNPs)	Preclinical	Microbial synthesis, antimicrobial, anticancer textiles	Medical textiles	Darwesh et al., 2024 [295]
6.40 ± 1.89 nm ($Ni_{0.85}Se$ NPs)	Preclinical	Fast clearance, NIR PTT, pH-responsive DOX release	Breast cancer therapy	Wang et al., 2017 [197]
~10 nm (released QDs)	In vitro & in vivo (mice), multistage system	Protease-triggered "shrink" to 10 nm for deep tumor penetration	Tumor delivery/ penetration model	Wong et al., 2011 [164]
~10 nm AuNPs	In vitro	pH-triggered intracellular aggregation; NIR photothermal conversion	Photothermal cancer therapy	Nam et al., 2009 [296]

5–7 nm (CuO-doped Ca-silicate)	In vitro	Size down-tuning by CuO; strong cytotoxicity	Anticancer; altered doxy-cycline release	Mabrouk et al., 2019 [297]
9.6 ± 0.5 nm AgNPs (in CChG)	In vitro	Polymer embedding; improved thermal stability	Antimicrobial composite; reduced cytotoxicity	Salama et al., 2016 [298]
~11 nm Ga_2S_3-BSA	In vitro & in vivo (ovarian models)	Acid-triggered Ga^{3+} + H_2S release; ferroptosis + apoptosis	Drug-resistant ovarian cancer therapy	Tang et al., 2025 [299]
13.2 ± 4.72 nm Ag[illegible]	In vitro	High negative zeta; potent [illegible] mor cytotoxicity	Antitumor (HeLa, U937)	Kaba et al., 2015 [300]
5.44 nm SPION–fo[illegible] paclitaxel	Preclinical	Radiosensitization in proton therapy	Brain cancer treatment	Kang et al., 2018 [301]
12.8 nm (mixed micelles)	In vitro & in vivo (H460/TaxR mice)	DSPE-PEG/TPGS micelles; [illegible] inhibition; high DOX EE (98.2%)	Overcoming MDR; enhanced tumor apoptosis	Jin et al., 2015 [302]
~9.3 nm (ZnO NPs)	Materials Characteriza-tion	Core properties preserved (Eg≈3.44 eV) while surface chemistry tuned	Platform for bio-interfaces & sensor[illegible]	Punnoose et al., 2014 [303]
~12–15 nm (SPION cores)	In vivo (xeno-grafts)	Very high SAR/ILP; efficient tumor heating with moderate dosimetry	Magnetic hyper-thermia (MDA-MB-231; BxPC-3)	Kossatz et al., 2014 [304]
Zn0.4Fe2.6O4 (sub-15 nm)	In vitro	Very high magnetization (≈142 emu g^{-1}); reduced lattice strain; superior photothermia	Photothermal & magnetothermal cancer therapy	Kasparis et al., 2023 [305]
4 nm (MnO core, hydrodynamic <20 nm)	In vitro (multiple lines)	PEG-phospholipid shell; low cytotoxicity; ROS gene pression profiling	Diagnostic probes; nanocarriers	Choi et al., 2015 [306]
6–12 nm ($CaWO_4$ nanocrystals; sub-15 nm)	In vitro (photo catalytic)	Block-copolymer encapsula tion; light-triggered ROS generation	Radiosensitization [illegible] photodynamic-like therapy	Lee et al., 2016 [307]
— (AgNP-MTX; ~10–15 nm typical)	In vitro & zebrafish	Methotrexate conjugation; controlled release; enhanced apoptosis	Chemotherapy (co-lon/lung models); zebrafish toxicity profiling	Rozalen et al., 2020 [308]
5–15 nm (γ-Fe_2O_3), optimum ~15 nm	Physical chara ization (magnetic losses)	Size-dependent heating; balance of Néel/Brownian losses; optimal SAR near ~15 nm	Magnetic hyperthe[illegible] mediators	Lévy et al., 2008 [309]
~10 nm Fe_3O_4 (in thermo/pH-sensitive hydrogel[illegible]	In vitro	Magnetically responsive; pH/temperature-triggered Dox release (↑ at 40 °C, acid[illegible]	Controlled doxorubicin delivery	Davaran et al., 2014 [310]
10.5 ± 5.6 nm Au–[illegible] (TRF-functionaliz[illegible]	In vitro	Water-dispersible; +35 mV [illegible] transferrin targeting; efficient uptake; Dox loading	Targeted doxorubicin delivery	Hameed et al., 2025 [311]
~11.66 nm	Preclinical	pH-sensitive drug release; high loading efficiency	DOX-loaded carbon-based NPs	Dechsri et al., 2023 [312]
15 nm	Preclinical	Induces oxidative stress;	SiO_2 NPs for	Ahamed et al., 2013 [313]

		apoptosis in epithelial cells	biomedical applications	
~10 nm	Preclinical	Enhanced siRNA delivery; toxicity	GO–polymer nanoc jugates	Yadav et al., 2018 [314]
5 nm core	Preclinical	BBB penetration; theranostic	TAT–Au NPs for glioma therapy	Cheng et al., 2014 [315]
10 nm	Preclinical	pH-triggered drug release; magnetic guidance	Fe_3O_4 nanocarriers	Gawali et al., 2019 [316]
8–20 nm	Preclinical	Anticancer; radiosensitizing	Gallium NPs + γ-radiation	Kandil et al., 2018 [317]
5–10 nm	Preclinical	Antioxidant; antimicrobial; ticancer	IH–AgNPs	Netala et al., 2018 [318]
10 nm	Preclinical	Synergistic with cisplatin; apoptosis/autophagy	rGO–AgNP nanocomposites	Yuan & Gurunathan, 2017 [319]
Sub-20 nm (5–15 nm relevant)	Preclinical research	Tunable surface charge; optimized charge density; enhanced cytosolic localization	Cancer drug delivery	Vo et al., 2024 [13]
6 nm	Preclinical research	Dose-dependent cytotoxicity; oxidative stress; apoptosis gene upregulation	HepG2 cytotoxicity model	Ahmad et al., 2020 [92]
5–7 nm	Preclinical research	Magnetic–plasmonic; light-gered release; >98% encapsu tion	Chemo–phototherr therapy	Rio et al., 2021 [320]
10 nm	Preclinical research	Green biosynthesis; antimicrobial; cytotoxic vs A549	Antimicrobial & lung cancer	Dobrucka et al., 2017 [321]
6.6 nm	Preclinical research	$\alpha v\beta 3$-targeted PDT; deep tumor penetration	Photodynamic cancer therapy	Zhao et al., 2018 [322]
12.6 nm	Preclinical research	Pullulan–Au hybrid; folate geting; pH-responsive DOX release	Targeted cancer delivery	Laksee et al., 2020 [323]
10 nm	Preclinical research	Size-dependent tissue uptake; exposure-dependent toxicity	Toxicology of AuNPs (rats)	Abdelhalim et al., 2015 [324]
13 nm	Preclinical research	Green AgNPs; antioxidant/ anti-inflammatory; NOTCH2 targeting (in silico)	Brain cancer (GBM/LGG) concept	Naveed et al., 2024 [325]
10 ± 2 nm	Preclinical research	Green synthesis; stable ζ-potential; anti-proliferative vs prostate cancer	Prostate cancer therapy	Vodnik et al., 2021 [326]
15 nm	Preclinical research	Radiosensitization; non-toxic intracerebral dose	Glioma radiotherapy	Bobyk et al., 2013 [327]
~15 nm	Preclinical research	Scintillation; X-PDT; dosim	Cancer therapy	Isikawa & Guidelli, 2022 [328]
~10 nm	Preclinical research	Chitosan-coated SPIONs; MTX loading	Targeted chemotherapy	Mohammadi-Samani et al., 2013 [329]

≤10 nm	Preclinical research	Size-dependent tumor penetration (dendrimers)	Drug delivery into spheroids	Bugno et al., 2019 [163]
8–12 nm	Preclinical research	CytC/CL nanospheres; molten-globule protein stat	Apoptosis mechani studies	Vladimirov et al., 2019 [330]
5–6 nm	Preclinical	Low polydispersity; negative surface charge; trastuzumab targeting	Targeted HER2+ cancer therapy	Miyano et al., 2010 [331]
5–15 nm	Preclinical	Aloe vera synthesis; anisotropic shapes; antimicrobial	Antimicrobial coatings	Logaranjan et al., 2016 [332]
<10 nm	Preclinical	Dendrimer stabilized; pure Au; NIR absorption	Photothermal cancer therapy	Wang et al., 2016 [333]
~20 nm AuNPs	In vitro	pH-triggered intracellular aggregation; NIR photothermal conversion	Photothermal cancer therapy	Cheng et al., 2021 [334]
10 nm mesoporous silica NPs	Preclinical	High uptake via caveolae-mediated endocytosis; cytoplasmic relocation; minimal surface coating favors uptake	Cancer theranostics	Ekkapongpisit et al., 2012 [335]
13.45 ± 1.42 nm	Preclinical	Surface-modified ZnO; reduced cytotoxicity to normal cells	Triton-X-100 modified ZnO NPs	Kc et al., 2016 [336]
~10 nm (Gold nanoparticles)	Research	Surface plasmon resonance high photothermal convers efficiency	Photothermal therapy for cancer	Huang et al., 2006 [58]
10–15 nm subset (Lipid nanoparticles)	Research	Biodegradable, efficient nucleic acid delivery	mRNA vaccines	Hou et al., 2021 [39]
~12 nm (Gold–silica nanoshells)	Research	Near-infrared absorption, tunable core–shell ratio	Photothermal ablation therapy	O'Neal et al., 2004 [337]
~7 nm (Polymeric micelles)	Research	Self-assembly, hydrophobic core	Solubilization of hydrophobic drugs	Kataoka et al., 2001 [338]
10 nm	Preclinical	SiNPs show low cytotoxicity, no genotoxicity or hemolysis.	Vehicles for drugs or gene delivery to liver cells.	Tüncel et al. 2021 [339]
5–15 nm subset (PEGylated gold nanoparticles)	Research	Size & PEG length-tuned BBB permeation	Brain tumor targeting	Etame et al., 2011 [340]
<14 nm (Cationic nanohydrogel particles)	Research	Efficient siRNA complexation & delivery, non-toxic	Gene therapy (CD8+ T cells)	Tabujew et al., 2019 [341]
~10 nm grains (multi-core)	Preclinical	Multi-core iron oxide nanoflowers; high SAR (~1 kW/g)	Magnetic hyperthermia for cancer	Bejko et al., 2024, [342]
5 nm	In vivo	pH-responsive; high drug loading; enhanced cellular uptake	5-FU, Sorafenib, PDT/photothermal, Drug delivery	Mezher et al., 2024 [343]
11 nm	In vivo	green/biogenic synthesis; size-switchable; cell-	ICG, PDT/ photothermal,	He et al., 2022 [344]

		penetrating peptide	Drug delivery	
5– 15 nm	In vivo	green/biogenic synthesis; enhanced cellular uptake; antioxidant	Anticancer	Govindaraju et al., 2015 [345]
5–15 nm	In vivo	green/biogenic synthesis; PEGylated/stealth; PDT/photothermal	PDT/photothermal, Antimicrobial, Anticancer	Amina et al., 2021 [346]
5– 15 nm	In vivo	deep tumor penetration; enhanced cellular uptake	platinum, Drug delivery	Liu et al., 2018 [347]
5–15 nm	In vivo	green/biogenic synthesis; enhanced cellular uptake; PDT/photothermal	PDT/photothermal, Antimicrobial, Anticancer	Alam et al., 2021 [348]
5–15 nm	In vivo	green/biogenic synthesis; high drug loading; enhanced cellular uptake	Doxorubicin, Anti-cancer, Drug delivery	Laksee et al., 2018 [63]
5–15 nm	In vivo	pH-responsive; high drug loading; enhanced cellular uptake	Doxorubicin, PDT/ photothermal, Anticancer, Drug delivery	Fazilati et al., 2014 [349]
5–15 nm	Clinical	enhanced cellular uptake; PDT/photothermal; antimicrobial	PDT/photothermal, Antimicrobial, Anticancer	Mohandoss et al., 2022 [350]
5–15 nm	In vivo	green/biogenic synthesis; PDT/photothermal; antimicrobial	PDT/photothermal, Antimicrobial, Anticancer	Bishoyi et al., 2025 [351]
5–15 nm	In vivo	PDT/photothermal; colloidal stability	PDT/ photothermal	Vasilakaki et al., 2018 [352]
5–15 nm	In vivo	PDT/photothermal; antimicrobial; anticancer/ cytotoxic	PDT/photothermal, Antimicrobial, Anticancer	Abdelrahman et al., 2024 [353]
9.39 nm	In vivo	green/biogenic synthesis; PDT/photothermal; antimicrobial	PDT/photothermal, Antimicrobial, Anticancer	El-Naggar et al., 2017 [354]
15 nm	Preclinical	High DNA loading (>1000 drugs/NP); controllable drug release	Daunomycin delivery for cancer therapy	Whitener et al., 2021 [355]
6.8 ± 1.2 nm	Preclinical	Tb–Rose Bengal coordination; high ROS yield under X-ray	X-ray induced Photodynamic therapy	Maiti et al., 2023 [356]
9.4 nm	Preclinical	Water-soluble; stable to heat & freeze–thaw; anti-inflammatory	Anti-inflammatory anti-proliferative (bladder cancer)	Chang et al., 2019 [357]
~10 nm (AuNP + coating)	Preclinical	Multivalent hydrocarbon chains; high ROS; resistance-free	Cancer nanodrug therapy	Jana et al., 2021 [66]
~14 nm	Preclinical + in vivo	Nuclear localization; enhances RT & ICD; macrophage infiltration	Radiotherapy + immunomodulatio[illegible] TNBC	Janic et al., 2021 [67]
11.9 ± 3.4 nm	Preclinical	Green synthesis (plant leav[illegible] stable; anticancer	Breast cancer inhibition (MCF-7)	Mardina et al., 2024 [69]

5–15 nm (albumin spheres)	Preclinical	Salt-precipitation synthesis; stable; non-cytotoxic	Drug nanocarrier; cytostatic delivery	Kudłacik-Kramarczyk et al., 2021 [358]
~10 nm (dendrimer fragments)	Preclinical	MMP-2 cleavable; size shrinkage from 200→10 nm; enhanced penetration	Solid tumor penetration	Han et al., 2017 [359]
~14 nm (AgNPs)	Preclinical	Green synthesis (With ania coagulans); antioxidant & antimicrobial	Anticancer (SiHa cervical cells)	Tripathi et al., 2019 [73]
~15 nm (poly amide–amine–DOX complex)	Preclinical	pH-triggered release; improved deep tumor delivery	DOX + fruquintinib synergistic therapy	Zhang et al., 2022 [360]
~9 nm	Preclinical	MoS_2 nanodots; photothermal + ROS synergy	Synergistic photothermal–photodynamic therapy	Liu et al., 2016 [361]
6–12 nm	Preclinical	Copper sulfide nanodots; strong NIR absorption	Photothermal therapy	Mou et al., 2015 [362]
~4-8 nm (Bi_2Se_3) nanorods	Preclinical	Bi_2Se_3 nanodots; radio-sensitizers; high X-ray attenuation	Tumor radio-photothermal therapy	Mao et al., 2016 [363]
~10 nm	Preclinical	Pd nanosheets; photothermal conversion; rapid clearance	In vivo cancer phototherapy	Tang et al., 2014 [364]
~10 nm	Preclinical	CoFe2O4 NPs coated with γ-CD; tunable hyper-thermia	Magnetic hyper-thermia for cancer	Caizer et al., 2022 [365]
7–10 nm	Preclinical	MoO3 nanodots; pH-responsive degradation; ROS	Chemo–chemo-dynamic therapy	Wang et al., 2018 [366]
~2 nm Pt nnanorods	Preclinical	Pt nanodots; ultrasmall size; renal clearance; ROS generation	Cancer catalytic therapy	Liao et al., 2021 [367]
5–12 nm	Preclinical	Co nanodots; high magnetic moment; catalytic properties	Synergistic chemo–magnetic therapy	Zhao et al., 2023 [368]
5–10 nm (polymeri nanoparticles)	Preclinical	Controlled release; biodegradable polymers; stable dispersions	Doxorubicin; cisplatin delivery	Sahoo et al., 2007 [369]
~8–12 nm (mesoporous silica nanoparticles	Preclinical	Large surface; tunable pores; controlled release	Camptothecin; doxorubicin delivery	Lu et al., 2007 [370]
10–12 nm (gold nanoshells)	Clinical (Phase I)	Tunable plasmon resonance; photothermal heating	Thermal ablation (prostate cancer)	Hirsch et al., 2003 [371]
7–9 nm (chitosan	Preclinical	Mucoadhesive;	Insulin oral	Sarmento et al., 2007 [372]

nanoparticles)		biodegradable; ionic gelation	delivery; peptides	
10–15 nm (ceramic nanoparticles)	Preclinical	Stable; slow degradation; surface modification	Controlled anticancer release	Vallet-Regí et al., 2001 [373]
6–10 nm (calcium phosphate nanoparticles)	Preclinical	Biodegradable; pH-sensitiv dissolution	Gene/DNA delivery	Sokolova & Epple, 2010 [374]
~12 nm (PLGA nanoparticles)	Preclinical	Biodegradable polymer; controlled release	Anticancer agent delivery	Hans & Lowman, 2002 [375]
10–15 nm (protein nanoparticles)	Preclinical	Self-assembly; uniform size multivalent surface	Vaccine carriers; enzyme encapsulation	Douglas & Young, 2006 [376]
5–8 nm (poly(ami-doamine) dendrimers)	Preclinical	Highly branched; multivalent; size control	Anticancer/nucleic delivery	Duncan & Izzo, 2005 [377]
10–15 nm (hollow nanocages)	Preclinical	Tunable NIR absorption; ho low interior	Photothermal ablation; drug loading	Chen et al., 2005 [378]
7–10 nm (fullerene-based nanoparticles)	Preclinical	Radical scavenging; photod namic	Antioxidant therap cancer PDT	Jensen et al., 1996 [379]
12–15 nm (polymeric nanogels)	Preclinical	Swelling response; high drug loading	Protein/hydrophili drug delivery	Vinogradov et al., 2002 [380]
5–8 nm (nickel ferrite nanoparticles)	Preclinical	Magnetic; high coercivity; tunable Ms	Hyperthermia; magnetic targeting	Gupta & Gupta, 2005 [381]
10–15 nm (PEG-modified nanoparticles)	Clinical (approved excipient)	Stealth; prolonged circulati reduced opsonization	Doxil®; PEGylated osomes	Harris & Chess, 2003 [382]
6–10 nm (chitosan nanopart cles)	Preclinical	Mucoadhesive; biodegrada controlled release	Insulin oral delivery; anticancer	Agnihotri et al., 2004 [383]
8–11 nm (poly-caprolactone nanoparticles)	Preclinical	Biodegradable polyester; slow release	Anticancer/ antifungal delivery	Sinha et al., 2004 [384]
5–8 nm (graphene ide nanosheets)	Preclinical	High surface; photo-thermal; versatile chemistry	Gene delivery; PTT	Zhang et al., 2010 [385]
7–9 nm (poly (β-amino ester) nanoparticles)	Preclinical	Cationic; biodegradable; efficient nucleic acid delivery	siRNA/DNA delivery	Lynn & Langer, 2000 [386]
6–9 nm (dendritic polyglycerol nanoparticles)	Preclinical	Highly branched; hydrophilic; multivalent	Anti-inflammatory delivery; diagnostics	Calderón et al., 2010 [387]
9–11 nm (siRNA-loaded cyclo-dextrin nanoparticles)	Clinical (Phase I)	Biodegradable; cationic; gene delivery	CALAA-01 (solid tumors)	Davis et al., 2010 [388]

12–15 nm (tannic acid–based nanoparticles)	Preclinical	Natural polyphenol; antiox dant; protein binding	Anticancer delivery antimicrobial	Fan et al., 2025 [389]
7–9 nm (selenium ı noparticles)	Preclinical	Antioxidant; ROS modulation; immunomodulatory	Cancer prevention; timicrobial	Zhang et al., 2005 [390]

Table S2: List of sub-15 nm ultrasmall nanoparticles investigated as diagnostics

Typical Size Range	Development Status	Unique Properties	Example Drugs / Applications	References
10 nm (s-SPIONs)	Preclinical	Steric stabilization, superparamagnetism, tumor accumulation	Ovarian tumor model, MRI	Pham et al., 2018 [391]
5 nm core (Fe_3O_4 SPIO)	Clinical-stage agent (Endorem)	Dual-modality MRI/SPECT, bisphosphonate linker	Imaging agent	Torres Martin de Rosales et al., 2011 [392]
11.3 nm (AuNPs)	Preclinical	Green synthesis, photoluminescence, pH-dependent release	Resveratrol conjugate	Kumar et al., 2014 [393]
11.61 nm (AuNP dendrimers)	Preclinical	Dual CT/MRI imaging, RGD targeting	Breast cancer imaging	Liu et al., 2021 [33]
~10 nm (Silica NPs)	Preclinical	Dye-doped, high-sensitivity, robust biosensor	Biosensing/imaging	Riccò et al., 2018 [394]
~15 nm AuNPs	In vitro (multiple cell types)	Biogenic (Fusarium); −35.8 mV zeta; selective uptake in 4T1	Tumor-targeted carriers	Pourali et al., 2024 [395]
4–6 nm CdTe QDs	In vitro	Thymine-conjugated; tunable fluorescence	Biosensing (adenine markers)	Rodzik et al., 2017 [89]
"<20 nm" dye-loaded polymeric NPs (ultrasmall)	In vitro	2–100× brighter than QDs; fast AmpliFISH	Rapid multiplex RNA imaging	Egloff et al., 2022 [90]
15 nm TiO_2 NPs	Preclinical	High refractive index; optical imaging	Solid immersion lens for super-resolution	Wang et al., 2023 [234]
11.8 nm EGF–ferritin NPs	Preclinical	EGFR-targeted; high biosafety	Breast cancer imaging & targeting	Li et al., 2012 [396]
9–13 nm quantum dots	Preclinical	Optimized for receptor imaging	Neuronal synapse labeling	Le et al., 2020 [397]
9.35 nm ESIONs (micelle-encapsulated)	Preclinical	T_1 MRI contrast; hepatobiliary clearance	MRI contrast agent	Suh et al., 2024 [398]
~12 nm (USPIO)	Imaging study (MR liver cancer)	Arabinogalactan-coated USPIO; ASG-receptor targeting; marked T2 reduction	MRI liver tumor contrast enhancement	Reimer et al., 1990 [399]

— (Review, QDs/IONPs within 5–15 nm)	Review	Multifunctional QDs/NPs; tumor-targeted imaging; multi-agent conjugation	Noninvasive in vivo tumor imaging/targeting	Rhyner et al., 2006 [400]
~5-10 nm (ultrasmall silica)	In vivo (sentinel lymph node mapping)	Stable fluorescence labelling; rapid lymphatic uptake and renal clearance	Sentinel lymph node mapping	Quan et al., 2017 [401]
5–6 nm (N-CQDs)	In vitro	Amphiphilic; pH-responsive fluorescence; high quantum yield	Bioimaging; sensing	Alzahrani et al., 2025 [402]
8–12 nm (luminescent IONPs)	In vitro	Dual-modal (magnetic + fluorescence); rare-earth-doped IONPs	Cellular imaging; potential theranostics	Tufani et al., 2021 [403]
6 nm (TiO_2 primary), ~200 nm agglomerates	In vitro (PC-3M)	Multiple uptake pathways; intracellular fate assessed	Uptake & safety profiling	Thurn et al., 2011 [404]
Ultrasmall Si nanoparticles (5–15 nm context)	In vitro (optical imaging)	Linear/non-linear optical imaging (HR-SIM, micro-Raman, TPEF, CARS); rapid imaging; low overheating risk	Cancer cell optical diagnostics	Tolstik et al., 2016 [405]
~5 nm (at tumor site)	Preclinical	Tumor-specific size reduction; improved LN drainage	iCluster system for metastatic cancer	Liu et al., 2019 [406]
~5 nm	Preclinical	Ligand exchange without size change; renal clearance	Functionalized TiO_2 NPs	Cheyne et al., 2011 [407]
15 × 4.5 nm; 10 × 2.5 nm	Preclinical	Tunable plasmon resonance; absorption-dominated extinction	Small Au nanorods	Ali et al., 2012 [408]
10 nm; 12 × 15 nm	Preclinical	Integrin-targeted imaging	RGD-modified protein NPs	Ahn et al., 2014 [409]
<10 nm	Preclinical	High MRI relaxivity; safe	CaF_2 core–shell NPs	Liu et al., 2017 [410]
10 nm	Preclinical research	MRI + fluorescence; pH-triggered intracellular release	Cancer theranostics	Zou et al., 2010 [411]
5–15 nm	Preclinical research	Superparamagnetic; intrinsic fluorescence; multimodal MRI/MPF imaging	Biomedical imaging & diagnostics	Reynders et al., 2021 [412]
6 nm	Preclinical research	NIR fluorescence; multiplex ligands; PET + optical	Melanoma node imaging & surgery	Chen et al., 2019 [233]
~5 nm (CeOx:Gd NPs)	Preclinical	High T1 relaxivity (r1 ≈ 12.0 $mM^{-1}s^{-1}$ per Gd); ROS-scavenging due to Ce^{3+}/oxygen vacancies;	MRI contrast + antioxidant activity (ROS suppression)	Eriksson et al., 2022 [413]
<20 nm	Preclinical research	PVP/buffer size control; biodistribution shift	Sentinel node imaging	Kim et al., 2015 [414]

5 nm (core)	Preclinical research	PASP-IO; RGD targeting; dual PET/MRI	Tumor imaging	Lee et al., 2008 [415]
8.3 nm	Preclinical	Anti-CEA functionalized; stable magnetic fluid	Theragnostic for colorectal cancer	da Paz et al., 2012 [416]
15–16 nm	Preclinical	PEI-stabilized iron oxide; HA targeted	Targeted MR imaging	Li et al., 2014 [417]
9.5 nm (Alternaria sp. AuNPs); 13.6 nm (G. sessile AuNPs)	Preclinical	Green fungal-mediated synthesis; narrow size distribution; strong plasmonic absorption; enhanced SERS effect	Biosensors; surface-enhanced Raman spectroscopy (SERS)	Olvera-Aripez et al., 2024 [418]
11 nm	Preclinical	Gold nanoparticles disturb adhesomes; alter MMP/TIMP balance.	Theranostics with caution for cell migration/invasion effects.	Mulens-Arias et al. 2018 [419]
1.5–9 nm	Preclinical	Ultrasmall Ag2S QDs with tunable 500–1200 nm emission.	Integrin-targeted cancer imaging with high tumor-to-liver ratio.	Tang et al. 2015 [420]
12 nm	Preclinical	Highly doped UCNPs emit brightly under mild excitation.	In vivo tumor imaging with low phototoxicity.	Li et al. 2020 [421]
10 nm	Preclinical	Protein-rich media drive AuNP aggregation to ~100 nm.	Implications for nanoparticle dosing and uptake studies.	Sabuncu et al. 2012 [422]
~10 nm (Gold nanoparticles)		High reactivity, size-dependent toxicity, tissue interaction	Toxicology studies (cardiac tissue impact)	Abdelhalim, 2011 [423]
~10 nm (Gold nanoparticles)		Spherical, high surface reactivity, fluorescence bioaccumulation tracking	Organ bioaccumulation, diagnostic imaging	Abdelhalim, 2013 [159]
8.9 ± 2.1 nm (Iron oxide NPs)		Magnetic, stable with PEI coating, high MR relaxivity	MR imaging of tumors	Zhu et al., 2015 [424]
10–15 nm subset (Silver nanoparticles)		Green synthesis from sandalwood, crystalline, dual plant & cancer bioactivity	Plant growth promotion, cancer inhibition	Gowda et al., 2024 [425]
~6 nm	Preclinical	Monodispersed; spherical; stable; eco-friendly α-amylase synthesis	Imaging; catalysis; biomedical uses	Arunkumar et al., 2013 [426]
~12 nm	Preclinical	Protein-stabilized gold nanoclusters; antibody-conjugated; NIR fluorescence; long-term stability	Targeted fluorescent nano-bioprobes for AML detection	Retnakumari et al., 2011 [427]

6.41 ± 0.73 nm (hydrodynamic)	Preclinical	Thin layer-protected gold NPs; EGFR/ErbB2 targeting; dual imaging (photoacoustic, CT)	Multimodal imaging for esophageal cancer	Chen et al., 2021, [428]
15 nm	Preclinical	DNA-functionalized gold NPs; FRET-based hydroxyl radical probe	Cellular imaging; ROS detection	Tang et al., 2008 [429]
<10 nm (subset 5–15 nm)	Preclinical	Quantum dots; compact; antibody-conjugated; bright; photostable	Quantitative cytology; targeted cellular imaging	Le et al., 2020 [430]
5–15 nm	In vivo	biosensing	Biosensor	Kumar et al., 2016 [431]
5–15 nm	Clinical	renal-clearable	Imaging	Chen et al., 2017 [432]
5 nm	In vivo	MRI contrast	MRI contrast, Imaging	Ma et al., 2022 [433]
5–15 nm	In vivo	enhanced cellular uptake; low toxicity/biocompatible; colloidal stability	Imaging	Wang et al., 2013 [434]
<10 nm	Preclinical	Strong blue fluorescence; enhanced PL after reduction; low toxicity	Bio-imaging of nuclei in cells	Li et al., 2022 [435]
10–15 nm (Au NPs)	Preclinical	Induce endothelial leakiness (NanoEL); size-dependent effects	Enhance nanomedicine access to tumors	Setyawati et al., 2017 [68]
~8.6 nm	Preclinical	Triptorelin-targeted; high CT contrast; safe to 200 μg/mL	Cancer molecular CT imaging	Mohammadi et al., 2022 [436]
5–10 nm	Preclinical	Ultrasmall Gd_2O_3 nanoparticles; high r1 relaxivity	MRI contrast agent	Yue et al., 2020 [437]
~11 nm	Preclinical	Silica nanodots; fluorescent labeling	Bio-imaging probes	Fu et al., 2024 [438]
5–10 nm	Preclinical	Fe_3O_4 nanodots; high r2 relaxivity; biocompatible	MRI tumor contrast	Xu et al., 2015 [439]
6–9 nm	Preclinical	Cu nanoclusters; strong fluorescence; low toxicity	Bio-imaging probes	An et al., 2020 [440]
~13 nm	Preclinical	Au nanoclusters; size-tunable luminescence; biocompatible	Bioimaging and sensing	Yuan et al., 2020 [441]
~8 nm	Preclinical	Gd nanodots; excellent r1 contrast	MRI contrast enhancement	Geng et al., 2021 [442]
~12 nm	Preclinical	Ag_2S nanoclusters; strong NIR-II fluorescence	Deep tissue imaging	Hong et al., 2012 [443]
9–13 nm	Preclinical	MnO nanodots; T1-weighted MRI agents	MRI tumor imaging	Na et al., 2007 [444]
~10 nm	Preclinical	Si nanodots; strong luminescence; biocompatible	Cellular imaging	Erogbogbo et al., 2011 [445]

5–15 nm	Preclinical	Au nanoclusters; tunable luminescence; ultrasmall size	Biosensing and bioimaging	Xie et al., 2009 [446]
~12 nm	Preclinical	Ag2Se nanodots; NIR-II emission; high penetration depth	Deep tissue fluorescence imaging	Ma et al., 2018 [447]
~9 nm	Preclinical	Mn nanodots; high r1 relaxivity; safe at low dose	MRI imaging of tumors	Stan et al., 2025 [448]
5–10 nm	Preclinical	ZrO_2 nanoclusters; strong biocompatibility; fluorescent	Cellular bioimaging	Jain et al., 2024 [449]
~7 nm (silica nanoparticle)	Clinical (first-in-human trial approved)	Multimodal (optical + nuclear imaging); cRGD targeting; clearance	Melanoma staging; lymphatic imaging; metastasis detection	Benezra et al., 2011 [450]
5–10 nm (quantum dot nanoprobes)	Preclinical (in vitro, in vivo)	Bright, stable fluorescence; tunable emission	Real-time cellular imaging; receptor tracking	Michalet et al., 2005 [451]
6–8 nm (iron oxide nanoparticle)	Preclinical	Superparamagnetic; high r2; biocompatible coating	MRI contrast; tumor imaging	Jun et al., 2008 [452]
6–8 nm (peptide-functionalized quantum dots)	Preclinical	Specific targeting; multiplex imaging	HER2 receptor tracking (breast cancer)	Gao et al., 2004 [453]
10–15 nm (iron oxide nanocrystals)	Clinical (approved contrast)	High relaxivity; T2 contrast; bio-coatings	Liver/spleen MRI	Weissleder et al., 1995 [454]
7–10 nm (magnetic iron oxide nanoparticles)	Clinical (Phase I/II)	Magnetic guidance; cell tracking; MRI	Stem cell tracking	Bulte & Kraitchman, 2004 [455]
10–14 nm (cerium oxide nanoparticles)	Preclinical	Antioxidant; ROS scavenging; radioprotective	Neuroprotection; anti-inflammatory	Das et al., 2007 [456]

Table S3: List of sub-15 nm ultrasmall nanoparticles investigated as theranostics

Typical Size Range	Development Status	Unique Properties	Example Drugs / Applications	References
~5–15 nm (Fe_3O_4@Au core-shell)	Preclinical	Magnetic + optical tunability, folic acid targeting	Targeted drug delivery	Karamipour et al., 2015 [457]
Pore size 13.52 nm (mesoporous silica)	Preclinical	High enzyme loading, pH-sensitive hybrid membrane, deep tumor penetration	Doxorubicin, Hyaluronidase for pancreatic cancer	Song et al., 2023 [458]
9 ± 1 nm (MFe_2O_4 ferrites)	Preclinical	Single spinel phase, superparamagnetic,	Magnetic biomedical applications	Ovejero et al., 2019 [459]

		composition-dependent cytotoxicity		
8–10 nm (Fe_3O_4, various coatings)	Preclinical	Superparamagnetism, tunable SAR, surface coating effects	Magnetic hyperthermia	Rajan et al., 2020 [460]
10 nm (Fe_3O_4 MNPs)	Preclinical	Surface charge-dependent toxicity, functional group tuning	Imaging, drug delivery	Yang et al., 2013 [461]
8.5–10 nm (Maghemite NPs)	Preclinical	Superparamagnetism, $Rh_2(suc)_4$ loading, BSA coating	Antitumor agent	Silva et al., 2018 [162]
<10 nm (carbon dots)	Review of preclinical studies	Multicolor fluorescence; easy surface functionalization; low toxicity	Tumor imaging; PDT/PTT; drug/gene delivery	Wang et al., 2018 [462]
15.7 ± 0.1 nm AgNPs	In vitro	Green synthesis (Mentha pulegium); monodisperse; high stability	Anticancer & antibacterial activity	Wang et al., 2021 [463]
~7 nm Mn_3O_4; ~15 nm Mn_3O_4–HfO_2	In vitro	Dual-modal MRI/CT contrast; ROS under low-power UV	Photodynamic therapy + imaging	Cui et al., 2024 [464]
10.2 ± 1.5 nm ZnO (in HA/ZnO)	In vitro	Green "seaweed" synthesis; hexagonal crystal	Anticancer (G2/M arrest; caspase 3/7)	Namvar et al., 2016 [465]
7–23 nm AgNPs (subset 7–15 nm)	In vitro	Plant-derived reducer; strong antibacterial & antioxidant	Antibacterial; antitumor (MCF-7)	AlSalhi et al., 2019 [466]
7.2 ± 3.3 nm AuNPs	In vitro	Curcumin-capped; stable in PBS/serum; RF/laser heating	Hyperthermia; apoptosis induction	Rezaeian et al., 2021 [467]
5–15 nm C-doped TiO_2	In vitro	Visible-light activation; ROS generation	Blue-light photodynamic killing (HeLa)	Matijević et al., 2021 [468]
8 nm & 15 nm Fe_3O_4	In vitro (HCT-116)	Superparamagnetic; AC-field heating	Magnetic hyperthermia	Attar et al., 2016 [469]
10 nm PEG-IONPs	In vitro & in vivo	Highest tumor uptake vs 30 nm; low toxicity (PEG)	MRI / drug delivery carrier	Feng et al., 2018 [470]
~13.5 nm Zn@Au	Preclinical concept	PET-imageable radiosensitizer (^{66}Ga); secondary electrons	PET-tracked radiotherapy	Cho et al., 2016 [471]
~13.4 nm (hydrodynamic) Gd_2O_3-based	In vivo (GBM model)	Very high r_1; BBB penetration; radiosensitization	MRI theranostics + radiotherapy	Shen et al., 2020 [204]
5.8–13.4 nm AuNPs	In vitro	1-minute microwave green synthesis (fucoidan); stable	Antitumor activity; dark-field imaging	Pinto et al., 2020 [169]
~10 nm Fe–Pt–Yb_2O_3 core–shell	Preclinical concept	Switchable superparamag→ferromag; β-emitters after activation	MRI contrast; magnetic targeting; radiotherapy	Seemann et al., 2023 [472]

<12 nm lipid–oxasmaragdyrin NPs	Preclinical	NIR fluorescence; photothermal therapy	Theranostic cancer imaging & treatment	Laxman et al., 2020 [473]
5–15 nm (design principles from PEG–SPIONs)	Preclinical	Dense PEG shell; low nonspecific binding	Targeted drug delivery & imaging	Gal et al., 2017 [474]
9 nm curcumin-loaded MNPs	Preclinical	High drug loading; MRI capability	Breast cancer therapy & imaging	Yallapu et al., 2012 [475]
12.5 nm Fe_3O_4 NPs (Pluronic-coated)	Preclinical	Superparamagnetic; dual targeting	Paclitaxel & curcumin co-delivery	Hiremath et al., 2019 [476]
8 nm Fe_3O_4@oleic acid	Preclinical	Superparamagnetic; hyperthermia & MRI	Magnetic nanocomposite capsules	Grillo et al., 2016 [477]
— (AuNP clustering inside cells)	In vitro (cellular)	Endocytosis-driven fractal rearrangement → NIR plasmon band; strong PTT	Photothermal therapy (808 nm)	Mulens-Arias et al., 2020 [478]
— (Review; 5–15 nm context)	Review	Comprehensive overview of nanoparticle roles in cancer Dx/Tx	Cancer diagnosis & therapy landscape	Lan et al., 2023 [479]
~10 nm (magnetite core → 17 nm after coating)	In vitro & in vivo (ovarian models)	APTES-PEG functionalization; ~70% DOX loading; ~90% release; tumor shrinkage	Targeted chemotherapy (ovarian)	Javid et al., 2014 [480]
— (Hollow carbon nanospheres; pores <10 nm)	In vitro	High payload of hydrophobics (e.g., erlotinib); tunable pores; reduced off-target	Chemotherapy (esophageal SCC)	Chen et al., 2021 [481]
~5 nm (Gd@graphene carbon NPs)	In vitro & rodent (PK/safety noted)	Dual imaging (strong fluorescence + high T1); high $^1O_2$ yield; renal clearance; low toxicity	Imaging-guided photodynamic therapy	Chen et al., 2018 [482]
— (Cu_2S quantum dots; size not stated in excerpt)	In vitro & in vivo (breast cancer models)	N-embedded Cu_2S QDs; photoluminescence; significant tumor growth inhibition; safety validated	Breast cancer theranostics (imaging + therapy)	Islam et al., 2025 [483]
~12 nm $CoFe_2O_4$	In vitro (induction heating)	OA surface functionalization; superparamagnetic; efficient magnetic fluid hyperthermia	Non-invasive cancer hyperthermia	Kharat et al., 2020 [484]
Subset 5–15 nm (NA-GNPs)	In vitro	Green one-step synthesis (Nigella arvensis); antibacterial; cytotoxic vs H1299/MCF-7	Antibacterial; anticancer nanoagents	Chahardoli et al., 2018 [485]
~10 nm	Preclinical	Plasmonic effect; NIR-triggered drug release	DOX-loaded Au NCs for theranostics	Lv et al., 2014 [486]

<10 nm	Preclinical	Strong fluorescence; biocompatibility	Carbon dots for imaging/drug delivery	Xu et al., 2024 [487]
2–10 nm	Preclinical	Unique optoelectronic properties	Quantum dots for diagnosis/therapy	Moetasam Zorab et al., 2023 [488]
12 nm	Preclinical	p53-independent apoptosis	SiO_2 NPs in HCT116	Fritsch-Decker et al., 2019 [489]
10 nm	Preclinical	Apoptosis induction; toxicity	PAA-coated AgNPs	Jakic et al., 2023 [490]
9 nm	Preclinical research	High drug loading; sustained release; improved stability	Cancer drug delivery	Dorniani et al., 2013 [491]
~10 nm	Preclinical research	Superparamagnetism; hyperthermia; luminescent hybrid	Cancer hyperthermia & imaging	Prasad et al., 2013 [492]
10 nm	Preclinical	Superparamagnetic; folic acid targeted; polymer coated	Targeted drug delivery, cancer therapy	Licciardi et al., 2013 [493]
~15 nm	Preclinical	Bio-fabricated; high antibacterial & anticancer activity	Antimicrobial; anticancer (HepG2)	Saratale et al., 2018 [494]
10–15 nm	Preclinical	Superparamagnetic; amino-functionalized; radiolabeled	Targeted radiotherapy for liver cancer	Liang et al., 2007 [495]
8 nm	Preclinical	Green synthesis; antibacterial; anticancer	Bacterial infections; gastric cancer	Van Hao et al., 2023 [496]
14.87 nm	Preclinical	Transferrin targeted; dual-drug (paclitaxel + β-elemene)	Non-small-cell lung cancer	Chen et al., 2024 [50]
15 ± 2.5 nm iron oxide NPs (nanocage, nanosphere)	Preclinical	Shape-dependent drug delivery; biocompatible; tunable surface; improved tumor penetration	Riluzole delivery for osteosarcoma	Raghubir et al., 2020 [497]
14.7 nm BisBAL NP (bismuth lipophilic nanoparticles)	Preclinical	High homogeneity; potent antitumor effect; no alopecia; favorable safety profile	Melanoma treatment	García-Cuellar et al., 2022 [498]
< 10 nm carbon quantum dots (CQDs)	Preclinical	High fluorescence; excellent biocompatibility; functionalizable for gene/drug delivery	Bioimaging; gene/drug delivery	Singh et al., 2018 [499]
9–15 nm PVP–palladium NPs	Preclinical	Polymer functionalization with PVP; monodisperse; spherical; face-centered cubic structure; ROS-mediated apoptosis	Anticancer (breast cancer MCF7 cells)	Ramalingam et al., 2020 [500]

~10 nm	Preclinical	High surface reactivity; cytotoxicity profile dependent on shape	Gold nanospheres	Woźniak et al., 2017 [501]
10 nm	Preclinical	High SPR; spherical; cytotoxic to MCF-7	AuNPs from plant extracts	Jannathul & Lalitha, 2017 [502]
<10 nm (Carbon dots)		Strong fluorescence, low toxicity, facile functionalization	Bioimaging, drug delivery	Li et al., 2012 [503]
~8 nm (Silver nanoparticles)		Broad-spectrum antimicrobial activity, high surface reactivity	Antibacterial coatings, wound dressings	Rai et al., 2009 [504]
~6 nm (Silica nanoparticles)		Tunable porosity, large surface area	Drug loading and sustained release	Slowing et al., 2008 [505]
5–12 nm (Dendrimer nanoparticles)		Precisely defined structure, multivalency	Gene delivery, drug conjugation	Patri et al., 2002 [506]
<15 nm (Upconversion nanoparticles)		Anti-Stokes emission, deep tissue penetration	Bioimaging, photodynamic therapy	Wang et al., 2011 [507]
5 nm	Preclinical	Rapid cellular entry; clathrin and caveolin-mediated uptake pathways.	Targeted drug delivery and diagnostic imaging in vitro.	Wu et al. 2019 [508]
<10 nm cores	Preclinical	Superparamagnetism at <10 nm; clustering increases relaxivity and SAR.	MRI contrast enhancement and magnetic hyperthermia in mice.	Hayashi et al. 2013 [509]
13.5 nm	Preclinical	Riboflavin-targeted AuNPs probed by AFM show size shifts.	Targeted imaging and delivery via riboflavin receptor binding.	Witte et al. 2014 [510]
12 nm	Preclinical	Spherical AgNPs; UV–vis at 430 nm; pro-apoptotic signaling.	In vitro anticancer activity in MDA-MB-231 cells.	Ghandehari et al. 2019 [511]
5.85 ± 0.84 nm (3.69–16.11 nm)	Preclinical	Crystalline AgNPs with 2.22 eV bandgap and strong plasmon.	Antimicrobial, antioxidative, and anticancer activities in vitro.	Mistry et al. 2022 [512]
~15 nm	Preclinical	Biogenic AgNPs induce ROS and apoptosis-related gene changes.	Anticancer efficacy in MCF-7 and HCT-116 cells.	Khan et al. 2021 [513]
1–20 nm (5–15 nm subset)	Preclinical	Intramolecularly collapsed SCNPs mimic protein-sized carriers.	Controlled drug delivery and targeted imaging platforms.	Kröger et al. 2018 [14]
<20 nm (subset 5–15 nm)	Preclinical	Carbon nanocrystals exhibit strong multicolor photoluminescence.	Cancer therapy and bioimaging in vitro.	Kajani et al. 2018 [514]

5–15 nm (Quantum dots)		Luminescent, size/composition-tunable optical properties, selective tissue targeting with peptides/PEG	Multiplexed imaging, targeted drug delivery	Akerman et al., 2002 [515]
~5 nm (Gold nanoparticles)		PEG + peptide functionalized for nuclear targeting, radiosensitization potential	Radiosensitization in osteosarcoma	Bures et al., 2020 [516]
5–15 nm subset (Silver nanoparticles)		Bio-capped with mace extract, antimicrobial, cytotoxic	Antibacterial, anticancer	Rizwana et al., 2021 [517]
5–15 nm	Preclinical	Spherical; high surface-to-volume ratio; stable with phytochemical capping	Antibacterial (E. coli, P. aeruginosa, S. aureus, B. subtilis, C. albicans); anticancer (HDF, U118, CaCo2, Skov-3)	Xu et al., 2023, [518]
10.8 ± 0.3 nm	Preclinical	Uniform quasi-spherical; green algal synthesis; high colloidal stability	Anticancer (colon); antibacterial (Gram-negative, Gram-positive)	Hamida et al., 2023, [519]
0.1–10 nm (subset 5–10 nm)	Preclinical	Spherical; protein-capped; SPR peak at 450 nm	Anticancer (Hep-2C); antiviral (rotavirus); immunomodulatory (↑IgA, IgM)	Adebayo-Tayo et al., 2019, [520]
~8 ± 2 nm	Preclinical	Uniform spherical; high negative zeta potential; green seed extract synthesis	Anticancer (HCT-116, PANC-1, MDA-MB-231)	Balkrishna et al., 2020, [521]
4–20 nm (subset 5–15 nm)	Preclinical	Spherical; UV-vis peak at 391 nm; antimicrobial & antibiofilm activity	Anticancer (MCF-7, HepG2); antifungal (C. albicans); antibacterial (E. faecalis, S. aureus, P. aeruginosa)	Alabssawy et al., 2024, [522]
7.1–26.68 nm (subset 7–15 nm)	Preclinical	Protein-capped; SPR peak at 430 nm; stable	Anticancer (Ehrlich ascites carcinoma); antibacterial	El-Naggar et al., 2018, [523]
10–50 nm (subset 10–15 nm)	Preclinical	Spherical/oval; green synthesis; high reactivity	Antibacterial; anticancer	Oves et al., 2023 [524]
<10 nm	Preclinical	ZnS quantum dots; autofluorescence; UV-activated ROS generation	Theranostic agent for imaging & photodynamic cancer therapy	Essawy et al., 2023 [525]
14 ± 5 nm (subset ~9–15 nm)	Preclinical	Ferrite NPs; polymer-coated (PEG, dextran,	Theranostic MRI contrast & hyperthermia for cancer	Zahraei et al., 2016, [526]

		chitosan); tunable relaxivity		
~5 nm	Preclinical	Ultrasmall gold nanoclusters; multi-stage-responsive; deep tumor penetration; dual imaging	Photothermal & enhanced radiation synergistic cancer therapy	Hua et al., 2021 [527]
9.2 ± 2.5 nm	Preclinical	Rhodamine & PAA-functionalized magnetite; glucose-modified; EV-coated	Cancer imaging & targeted drug delivery	Quiñonero et al., 2023 [528]
4.6 ± 1.7 nm	Preclinical	Curcumin-derived nanodots; bright photoluminescence; ROS regulation	Anticancer (A549); cellular bioimaging	Arvapalli et al., 2021 [529]
8.5 nm	Preclinical	Myco-fabricated Al_2O_3; spherical; crystalline; antimicrobial; anticancer	Antibacterial; anticancer therapy	Hassan et al., 2024 [530]
<10 nm (subset 5–15 nm)	Preclinical	Renal-clearable inorganic NPs; rapid clearance; limited drug loading	Imaging; potential surface-coupled prodrug delivery	Heneweer et al., 2012 [531]
5–15 nm	Preclinical research	radiotherapy dose enhancement; enhanced stability; high biocompatibility; targeted delivery	cancer therapy; advanced biomedical targeted therapy	Sinha et al., 2015 [532]
5–15 nm	Preclinical research	enhanced stability; high biocompatibility; targeted delivery	cancer therapy; advanced biomedical targeted therapy	Asadi et al., 2023 [533]
5 nm	Preclinical research	targeted; dual-functional; theranostic; enhanced stability; high biocompatibility; targeted delivery	cancer therapy; advanced biomedical targeted therapy	Kumar et al., 2023 [534]
5–15 nm	Preclinical research	targeted; dual-functional; plasmonic; enhanced stability; high biocompatibility; targeted delivery	cancer therapy; imaging; advanced biomedical targeted therapy	Khademi et al., 2019 [535]
5–15 nm	Preclinical research	superparamagnetic; targeted; biocompatible; enhanced stability; high biocompatibility; targeted delivery	cancer therapy; advanced biomedical targeted therapy	Affatigato et al., 2023 [536]
15 nm	Preclinical research	photothermal; photodynamic; chemodynamic; enhanced stability; high	cancer therapy; advanced biomedical targeted therapy	Feng et al., 2021 [537]

		biocompatibility; targeted delivery		
5–15 nm	Preclinical research	oxidase-like; enhanced stability; high biocompatibility; targeted delivery	diagnostics; biosensing; advanced biomedical application;	Ma et al., 2022 [538]
5–15 nm	Modeling study	targeted; radiotherapy dose enhancement; contrast agent; enhanced stability; high biocompatibility; targeted delivery	cancer therapy; advanced biomedical targeted therapy	Kirkby et al., 2015 [539]
5–15 nm	Preclinical research	biocompatible; enhanced stability; high biocompatibility; targeted delivery	cancer therapy; advanced biomedical targeted therapy	Liu et al. et al., 2018 [540]
15 nm	Preclinical research	dual-functional; quantum dots; green synthesis; enhanced stability; high biocompatibility; targeted delivery	cancer therapy; imaging; advanced biomedical targeted therapy	Shi et al., 2019 [541]
5–15 nm	Preclinical research	targeted; plasmonic; enhanced stability; high biocompatibility; targeted delivery	cancer therapy; detection; advanced biomedical targeted therapy	Nallathamby et al., 2010 [542]
5–15 nm	Preclinical research	dual-functional; green synthesis; biosynthesized; enhanced stability; high biocompatibility; targeted delivery	cancer therapy; advanced biomedical application; targeted therapy	Mittal et al., 2015 [543]
5–15 nm	Preclinical research	targeted; green synthesis; enhanced stability; high biocompatibility; targeted delivery	cancer therapy; advanced biomedical targeted therapy	El-Sonbaty et al., 2013 [544]
5–15 nm	Preclinical research	quantum dots; green synthesis; enhanced stability; high biocompatibility; targeted delivery	cancer therapy; advanced biomedical application;	Nakhaeepour et al., 2019 [545]
5–15 nm	Preclinical research	superparamagnetic; targeted; folate-targeted; enhanced stability; high biocompatibility; targeted delivery	cancer therapy; imaging; advanced biomedical targeted therapy	Chen et al., 2012 [546]
5–15 nm	Preclinical research	targeted; quantum dots; enhanced stability; high	cancer therapy; therapy; advanced	Alves et al., 2022 [547]

		biocompatibility; targeted delivery	biomedical targeted therapy	
5–15 nm	Preclinical research	targeted; enhanced stability; high biocompatibility; targeted delivery	cancer therapy; advanced biomedical targeted therapy	Devi et al., 2013 [548]
5–15 nm	Preclinical research	targeted; plasmonic; biocompatible; enhanced stability; high biocompatibility; targeted delivery	cancer therapy; advanced biomedical targeted therapy	Saravanakumar et al., 2019 [549]
5–15 nm	Preclinical research	superparamagnetic; photothermal; targeted; enhanced stability; high biocompatibility; targeted delivery	cancer therapy; advanced biomedical targeted therapy	Song et al., 2020 [550]
15 nm	Preclinical research	superparamagnetic; dual-functional; theranostic; enhanced stability; high biocompatibility; targeted delivery	cancer therapy; imaging; advanced biomedical targeted therapy	Castillo et al., 2014 [551]
5–15 nm	Preclinical research	targeted; dual-functional; plasmonic; enhanced stability; high biocompatibility; targeted delivery	cancer therapy; breast cancer; advanced targeted therapy	Majeed et al., 2019 [552]
5–15 nm	Preclinical research	targeted; dual-functional; theranostic; enhanced stability; high biocompatibility; targeted delivery	cancer therapy; imaging; advanced biomedical targeted therapy	Zhan et al., 2023 [553]
5–15 nm	Preclinical research	targeted; contrast agent; enhanced stability; high biocompatibility; targeted delivery	cancer therapy; imaging; advanced biomedical targeted therapy	Huang et al., 2013 [554]
5–15 nm	Preclinical research	targeted; dual-functional; theranostic; enhanced stability; high biocompatibility; targeted delivery	cancer therapy; therapy; advanced biomedical targeted therapy	Fantechi et al., 2014 [555]
7 nm	Preclinical research	targeted; dual-functional; quantum dots; enhanced stability; high biocompatibility; targeted delivery	cancer therapy; therapy; advanced biomedical targeted therapy	Tok et al., 2025 [556]
5–15 nm	Preclinical research	targeted; hyperthermia; enhanced stability; high	cancer therapy; therapy; advanced	Alphandéry et al., 2019 [557]

		biocompatibility; targeted delivery	biomedical targeted therapy	
5.3 ± 1 nm	Preclinical research	; enhanced stability; high biocompatibility; targeted delivery	cancer therapy; therapy; advanced biomedical targeted therapy	Tedesco et al., 2010 [558]
5–15 nm	Preclinical research	targeted; enhanced stability; high biocompatibility; targeted delivery	cancer therapy; therapy; advanced biomedical targeted therapy	Vyas et al., 2019 [559]
5–15 nm	In vivo	green/biogenic synthesis; PDT/photothermal; antimicrobial	PDT/photothermal, Antimicrobial, Anticancer, Drug delivery, Imaging	Lee et al., 2019 [560]
5–15 nm	In vivo	aptamer-targeted; PEGylated/stealth; high drug loading	Paclitaxel, Anticancer, Drug delivery, Imaging	Engelberg et al., 2019 [561]
15 nm	In vivo	PEGylated/stealth; enhanced cellular uptake; MRI contrast	MRI contrast, Drug delivery, Imaging	Lazarro et al., 2020 [562]
5–15 nm	In vivo	green/biogenic synthesis; albumin/BSA-stabilized; PDT/photothermal	PDT/photothermal, Imaging	Poderys et al., 2020 [563]
5–15 nm	In vivo	enhanced cellular uptake; PDT/photothermal; anticancer/cytotoxic	PDT/photothermal, Anticancer, Imaging	Steckiewicz et al., 2019 [61]
5–15 nm	In vivo	renal-clearable; low toxicity/biocompatible; colloidal stability	Drug delivery	Selim et al., 2025 [62]
5–15 nm	In vivo	key biomedical functions	Drug delivery, Imaging	Yu et al., 2014 [564]
5–15 nm	In vivo	PEGylated/stealth; enhanced cellular uptake; PDT/photothermal	PDT/photothermal, Drug delivery, Imaging	Ma et al., 2023 [565]
5–15 nm	In vivo	MRI contrast; PDT/photothermal	MRI contrast, PDT/photothermal, Drug delivery, Imaging	Glickson et al., 2008 [566]
5–15 nm	Clinical	CT imaging; PDT/photothermal; renal-clearable	CT imaging, PDT/photothermal, Imaging	Wang et al., 2019 [567]
5–15 nm	Clinical	PDT/photothermal; anticancer/cytotoxic; renal-clearable	PDT/photothermal, Anticancer, Drug delivery, Imaging	Hameed et al., 2022 [568]
5– 15 nm	In vivo	pH-responsive; enhanced cellular uptake; PDT/photothermal	Doxorubicin, PDT/photothermal, Anticancer, Drug delivery, Imaging	Venkatpurwar et al., 2011 [64]

5–15 nm	In vivo	PEGylated/stealth; anticancer/cytotoxic; colloidal stability	Anticancer, Imaging	Song et al., 2020 [569]
5–15 nm	In vivo	key biomedical functions	Therapeutic/diagnostic uses	Wang et al., 2017 [570]
5–15 nm	In vivo	high drug loading; enhanced cellular uptake; PDT/photothermal	PDT/photothermal, Drug delivery, Imaging	Vahedi et al., 2022 [99]
~10 nm Au core	In vitro study	Highly uniform core-shell with polymer	Plasmonic/Photothermal interface	Xing et al., 2009 [571]
5–15 nm	In vivo	green/biogenic synthesis; MRI contrast; CT imaging	Doxorubicin, MRI contrast, CT imaging, PDT/photothermal, Drug delivery, Imaging	Mishra et al., 2016 [572]
5–15 nm	In vivo	high drug loading; MRI contrast; PDT/photothermal	MRI contrast, PDT/photothermal, Drug delivery, Imaging	Kim et al., 2021 [573]
5–15 nm	Clinical	PDT/photothermal; antioxidant; renal-clearable	PDT/photothermal, Drug delivery, Imaging	Abdelhalim et al., 2011 [574]
10 nm	Clinical	MRI contrast; PDT/photothermal; anticancer/cytotoxic	MRI contrast, PDT/photothermal, Anticancer, Imaging	Wang et al., 2018 [575]
5–15 nm	In vivo	PDT/photothermal; antioxidant; low toxicity/biocompatible	PDT/photothermal	El-Fiqi et al., 2021 [576]
5–15 nm	Clinical	low toxicity/biocompatible	Therapeutic/diagnostic uses	Muzquiz-Ramos et al., 2013 [577]
5–15 nm	In vivo	high drug loading	Drug delivery, Imaging	Moharil et al., 2022 [578]
~10 nm	Preclinical (in vitro + in vivo)	LHRH functionalization; strong MRI T2 contrast; low cytotoxicity	Breast tumor MRI imaging, targeted cancer therapy	Nian et al., 2019 [579]
6.95 ± 2.25 nm	Preclinical	Green synthesis (ginseng extract); uniform size; functionalizable	Docetaxel chemo–photothermal therapy	Lee et al., 2023 [580]
~5 nm (MnO core)	Preclinical	High r1 relaxivity; dual MRI & fluorescence	Theranostic docetaxel delivery for breast cancer	Abbasi et al., 2015 [581]
9–14 nm	Preclinical	Superparamagnetism; citrate/malate coating; ROS under X-rays	X-ray enhancer for low-dose radiotherapy	Klein et al., 2014 [582]
5–6 nm	Preclinical	Plant extract synthesis; Ni doping increases cytotoxicity & SPF	Colon cancer cytotoxicity; UV protection	Miri et al., 2020 [583]

~5 nm (micelles, post-shrinkage)	Preclinical	NIR-triggered size shrinkage; theranostic (CT/MRI + therapy)	DOX delivery; tumor regression	Wu et al., 2024 [584]
6–10 nm (Bi(OH)3 & α-Bi2O3 NPs)	Preclinical	Selectively toxic to cancer cells; minimal effect on normal cells	Gliosarcoma 9L & MCF-7 therapy	Bogusz et al., 2018 [585]
~10 nm	Preclinical	Pegylated micelles with iron oxide; dual MRI/fluorescence	Apoptosis detection in vivo	van Tilborg et al., 2006 [586]
5–15 nm	Preclinical	Plant-mediated biosynthesis; crystalline; catalytic & antimicrobial	Biomedical and catalytic uses	Huo et al., 2018 [587]
~12 nm	Preclinical	Polyoxometalate nanoclusters; redox activity; ultrasmall size	Radiosensitization in cancer	Zhou et al., 2019 [588]
8–12 nm	Preclinical	Palladium nanoclusters; high photothermal efficiency	Photothermal cancer therapy	Thangudu et al., 2020 [589]
~13 nm	Preclinical	Ag nanoclusters; antimicrobial; ROS generation	Antibacterial and anticancer	Prateeksha et al., 2021 [590]
5–15 nm	Preclinical	Se nanoclusters; induce apoptosis; biocompatible	Cancer cell apoptosis	Pei et al., 2023 [591]
~7 nm	Preclinical	Ru nanoclusters; photothermal + photodynamic; apoptosis	Cancer phototherapy	Wederich et al., 2023 [592]
8–12 nm	Preclinical	Ir nanoclusters; photothermal stability; near-IR absorbance	Tumor ablation therapy	Xu et al., 2020 [593]
6–10 nm	Preclinical	TiO2 nanoclusters; photocatalytic ROS generation	Photodynamic therapy	Li et al., 2021 [594]
~9 nm	Preclinical	Carbon nanodots; strong fluorescence; low cytotoxicity	Bioimaging and cell labeling	Hola et al., 2014 [595]
5–15 nm	Preclinical	Ag nanoclusters; strong antimicrobial; biosynthesized	Antibacterial agents	Singh et al., 2016 [596]

<5 nm metal nanoclusters	Preclinical	Renal clearable inorganic nanoparticles	Bioimaging, biosensing and theranostics	Liu et al., 2013 [597]
5–15 nm	Preclinical	Copper oxide nanoparticles; strong photothermal; good biocompatibility	Photothermal ablation of tumors	Gao et al., 2021 [598]
5–12 nm (dendrimer-based nanoparticle)	Preclinical	High drug loading; precise architecture; multivalency	Methotrexate delivery; anticancer therapy	Patri et al., 2009 [599]
7–9 nm (carbon dots)	Preclinical	Strong photoluminescence; water soluble; low toxicity	Bioimaging; drug delivery	Sun et al., 2006 [600]
~12 nm (silver nanoparticles)	Preclinical	Antimicrobial; ROS generation; size-dependent cytotoxicity	Antibacterial coatings; anticancer testing	Morones et al., 2005 [601]
10–15 nm (protein-based nanoparticles)	Preclinical	Biocompatible; site-specific conjugation; enzymatic stability	Albumin NPs for paclitaxel	Kratz, 2008 [602]
5–10 nm (PEG-coated nanoparticles)	Preclinical	Stealth; prolonged circulation; reduced RES uptake	Paclitaxel; doxorubicin formulations	Owens & Peppas, 2006 [248]
10–15 nm (superparamagnetic nanoparticles)	Clinical (under evaluation)	Magnetic targeting; MRI visibility; external guidance	Hyperthermia; tumor imaging	Jordan et al., 1999 [603]
5–10 nm (silicon nanoparticles)	Preclinical	Photoluminescent; biodegradable; biocompatible	In vivo imaging; drug delivery	Erogbogbo et al., 2008 [604]
8–15 nm (virus-like nanoparticles)	Preclinical	Uniform; repetitive surface; high immunogenicity	Vaccines; gene therapy	Chatterji et al., 2002 [605]
5–8 nm (gold nanorods)	Preclinical	Anisotropic optics; tunable absorption	Photothermal therapy; biosensing	Murphy et al., 2008 [606]
8–10 nm (silica–gold nanoshells)	Clinical (Phase I)	NIR photothermal; biocompatible	Head/neck cancer ablation	Loo et al., 2004 [607]
~7 nm (DNA origami nanoparticles)	Preclinical	Programmable structure; precise targeting	Nucleic acid delivery; biosensing	Rothemund, 2006 [608]
8–12 nm (lipid–polymer hybrid nanoparticles)	Preclinical	Core–shell; stability; biocompatibility	siRNA/chemo delivery	Zhang et al., 2008 [609]
8–10 nm (zinc oxide nanoparticles)	Preclinical	UV absorption; ROS; antimicrobial	Antibacterial coatings; cancer therapy	Zhang et al., 2007 [610]
10–12 nm (carbon nanotube–based NPs)	Preclinical	High aspect ratio; functionalizable; conductive	Gene delivery; biosensing	Bianco et al., 2005 [611]
~9 nm (gelatin nanoparticles)	Preclinical	Biodegradable; low toxicity; functionalization	Anticancer delivery; vaccines	Coester et al., 2000 [612]

7–10 nm (gold nanoparticles)	Preclinical	Strong catalytic activity; radiosensitizer	Cancer radiotherapy; catalysis	Hainfeld et al., 2004 [613]
5–8 nm (iron–platinum alloy nanoparticles)	Preclinical	High magnetic moment; chemical stability	MRI contrast; magnetic hyperthermia	Sun et al., 2000 [614]
8–10 nm (polyoxometalate nanoparticles)	Preclinical	High negative charge; antiviral activity	HIV/cancer therapy	Rhule et al., 1998 [615]

Disclaimer/Publisher's Note: The statements, opinions and data contained in all publications are solely those of the individual author(s) and contributor(s) and not of MDPI and/or the editor(s). MDPI and/or the editor(s) disclaim responsibility for any injury to people or property resulting from any ideas, methods, instructions or products referred to in the content.
